# Activity-Based Dicyanoisophorone Derivatives: Fluorogenic Toolbox Enables Direct Visualization and Monitoring of Esterase Activity in Tumor Models

**DOI:** 10.1021/acs.analchem.4c04721

**Published:** 2024-11-01

**Authors:** Kavyashree P., Atri Bhattacharya, Lidong Du, Akshay Silswal, Moxin Li, Jiayue Cao, Qingqing Zhou, Weiming Zheng, Tzu-Ming Liu, Apurba Lal Koner

**Affiliations:** † Bionanotechnology Lab, Department of Chemistry, Indian Institute of Science Education and Research Bhopal, Bhopal Bypass Road, Bhauri, Bhopal 462066, Madhya Pradesh, India; ‡ Department of Chemistry, University of Texas at Austin, Austin, Texas 78712-1224, United States of America; § Institute of Translational Medicine, Faculty of Health Sciences & Ministry of Education Frontiers Science Center for Precision Oncology, University of Macau, Taipa, Macau 999078, China; ∥ Translational Medicine R&D Center, Zhuhai UM Science and Technology Research Institute, Zhuhai 519000, China

## Abstract

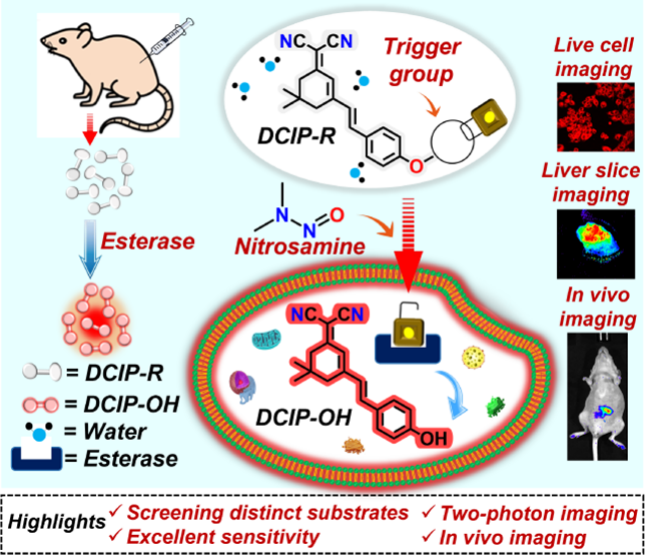

The visualization and spatiotemporal monitoring of endogenous esterase activity are crucial for clinical diagnostics and treatment of liver diseases. Our research adopts a novel substrate hydrolysis-enzymatic activity (SHEA) approach using dicyanoisophorone-based fluorogenic ester substrates **DCIP-R (R = R1–R6**) to evaluate esterase preferences on diverse substrate libraries. Esterase-mediated hydrolysis yielded fluorescent **DCIP–OH** with a nanomolar detection limit *in vitro*. These probes effectively monitor ester hydrolysis kinetics with a turnover number of 4.73 s^–1^ and catalytic efficiency (*k*_cat_/*K*_m_) of 10^6^ M^–1^ s^–1^ (**DCIP-R1**). Comparative studies utilizing two-photon imaging have indicated that substrates containing alkyl groups (**DCIP-R1**) as recognition elements exhibit enhanced enzymatic cleavage compared to those containing phenyl substitution on alkyl chains (**DCIP-R4**). Time-dependent variations in endogenous esterase levels were tracked in healthy and liver tumor models, especially in diethylnitrosamine (DEN)–induced tumors and HepG2-transplanted liver tumors. Overall, fluorescence signal quantifications demonstrated the excellent proficiency of **DCIP-R1** in detecting esterase activity both *in vitro* and *in vivo*, showing promising potential for biomedical applications.

## Introduction

The global incidence of liver cancer has steadily increased over the past two decades and is expected to surpass one million cases by 2025.^1^ Hepatocellular carcinoma (HCC) is responsible for more than 80% of all cases of primary liver cancer, with a diminished 5-year survival rate of 5%.^2,3^ Its occurrence is typically associated with long-term liver damage and cirrhosis. Key causes and risk factors include chronic viral hepatitis, alcohol abuse, metabolic disorders, environmental carcinogens such as tobacco and nitrosamines, and genetic disorders.^4−7^ Early diagnosis of HCC is vital for clinical evaluation and treatment, improving patient survival.^8−10^ Despite the widespread use of imaging techniques such as computed tomography and magnetic resonance imaging for the diagnosis of HCC, they continue to have major drawbacks such as radiation exposure and minimal soft-tissue contrast. An alternative approach to enhance diagnostic accuracy is to identify the biomarkers associated with HCC. One of the promising clinical biomarkers is the enzyme esterase, whose overexpression is closely related to hepatocellular tumorigenesis.^11−13^

Esterase is a member of the serine hydrolase superfamily and is typically found in the endoplasmic reticulum (ER) and cytoplasm of several organs, including the liver, gut, lungs, and kidneys.^8,14,15^ Functionally, they regulate the endogenous activation of ester-based prodrugs, such as clopidogrel, irinotecan, and oseltamivir, and facilitate the hydrolysis and elimination of xenobiotic compounds such as pesticides and environmental toxins.^16−19^ They also play a role in lipid regulation by metabolizing endogenous esters such as cholesteryl ester, chylomicron, and triacylglycerol.^20^ In clinical practice, dysfunctional esterases are intimately related to conditions including hepatic steatosis, atherosclerosis, cholesterol-induced liver injury, and type 2 diabetes.^21,22^ Most importantly, accumulating evidence revealed that elevated levels of esterase production are firmly linked to hepatocellular carcinoma.^9,23,24^ Therefore, developing a real-time detection technique to monitor esterase levels with high spatiotemporal resolution at the cellular, tissue, and organ levels holds promise for diagnosing HCC.

From this perspective, far-red emissive fluorescent probes offer a noninvasive detection method, rapid response times, and minimal background interference, making them an attractive prospect for identifying the bioanalytes of interest.^25−43^ Moreover, some probes with two-photon excitable characteristics provide additional advantages by reducing photodamage to cells and enabling deeper tissue penetration with high spatiotemporal resolution.^44−46^ Therefore, these probes are widely used for real-time monitoring of enzymatic activity, disease diagnosis, and evaluation of cellular response.^47−49^ Thus, due to the upregulation of esterase during HCC, an esterase-activable fluorogenic probe is required for image-guided diagnosis and therapy assessment, particularly in the liver.^46,50^ Such probes usually contain a fluorogenic core along with a functional group that gets cleaved by the enzyme, thereby releasing the fluorescent component. The effective unmasking of the fluorogenic probe and its strong biological performance primarily depend on the enzyme-cleavable group. Therefore, optimizing the structural properties of the activatable group is essential. In this direction, Guo et al. developed NIR fluorescent probes with varying cycloalkane esters as esterase-cleavable constituents; similarly, Yang et al. proposed a “probe-cavity matching strategy” for selective detection of esterase enzyme.^31,51^ Also, Yoon et al. reported the NIR-emissive, two-photon–active fluorogenic probe DCM-Cl-CE for imaging orthotopic HCC during chemotherapy.^46^ The earlier report (CYOH-R) required acetonitrile as a reaction medium along with HEPES buffer solution, whereas subsequent probes (HBT-CE) emit in the blue region, which is detrimental for *in vivo* imaging. To circumvent these limitations, we developed far-red–emitting, two-photon–active, biocompatible fluorescent probes for *in vitro* and *in vivo* detection of esterase under physiological conditions. Although esterase is known to hydrolyze ester, thioester, and amide bonds, we restricted our investigation to the esters of unbranched aliphatic carboxylic acids, considering their wide applicability as prodrugs.^52,53^

Consequently, enzyme detection, early disease diagnosis, and treatment innovations can tremendously benefit from scanning a vast array of substrates to identify the optimal “esterase-ester” combination.

Herein, we synthesized six ester derivatives of dicyanoisophorone (**DCIP-R1** to **DCIP-R6**). The esterase-mediated hydrolysis of the probes results in far-red “turn-on” emission *via* intramolecular charge transfer (ICT) with a detection limit in the nanomolar range. Steady-state UV–visible absorption, fluorescence, and kinetic experiments confirmed the esterase-mediated fluorogenic response, which is further validated by HRMS and HPLC analyses. Fluorescence kinetic experiments revealed a rapid response of probes toward porcine liver esterase (PLE) and human carboxylesterase 2 (hCEs2) with a very high enzymatic turnover number, indicating a strong affinity for the esterase. Ultimately, it was revealed that alkyl-trigger substrates performed better than substrates having a phenyl moiety on alkyl chains.

**Scheme 1:**
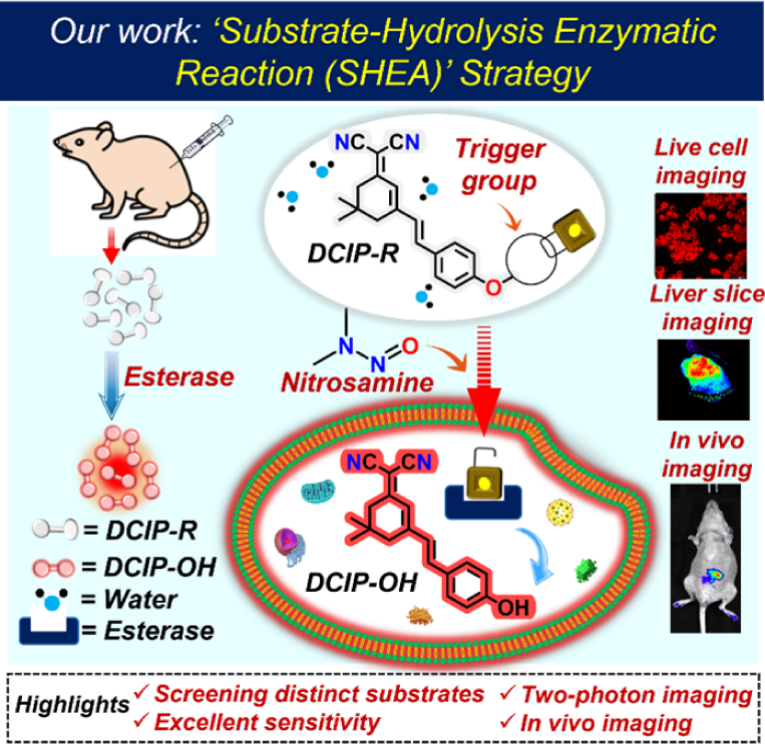
Schematic Representation of Endogenous Esterase Detection Using Dicyanoisophorone-Based Probes

Moreover, the outstanding reactivity and sensitivity of **DCIP-R1** were further exploited for *in vivo* imaging. The real-time application of these biocompatible probes was demonstrated through (a) one- and two-photon imaging of liver cancer HepG2 cells treated with **DCIP-R1** and **-R4**, (b) time-dependent monitoring of esterase levels in DEN-induced and HepG2-transplanted rat liver tumor models, and (c) *in vivo* tracking and imaging of endogenous esterase by intraperitoneally injecting probes into normal, healthy nude mice and xenograft HepG2 mouse models (Scheme 1). A comparison of representative fluorescence responses at different time points indicated that **DCIP-R1** is more effective than **-R4** for imaging esterase activity in mouse models.

## Experimental Section

### Materials and Synthesis

The materials used in this study are listed in the Supporting Information. The detailed synthesis procedure and characterization of **DCIP–OH** and **DCIP-R** (**R1–R6**) are provided in the Supporting Information.

### Steady-State Spectroscopic Measurements

All steady-state absorption measurements were performed using a SPECORD 210 Plus UV–Vis spectrophotometer from Analytik Jena, operated with ASpect UV 1.2.0 software. All steady-state and kinetic emission measurements were conducted with a HORIBA Jobin Yvon Fluorolog fluorimeter using FluorEssence V3.9 software. The temperature of the measurements was adjusted to 37 °C using the Quantum Northwest TC 1 temperature controller and T-App program. To simulate physiological conditions, measurements were performed at 37 °C using a JUMO dTRON 308 temperature controller.

### Concentration-Dependent Kinetics

10 μM of the probes (**DCIP-R1** to **-R6**) in HEPES buffer (100 mM, pH = 8.0) was used for the kinetic studies. Initially, the probes were excited at 480 nm, and the emission kinetics was monitored at 657 nm at 37 °C for 5 min; this was followed by the addition of the respective concentration of the enzyme (PLE or hCEs2), and the kinetics was monitored for 30 min.

### Enzyme Kinetics Assays

The Michaelis constant, *K*_m_, was obtained from measurements performed using a specific enzyme concentration (0.008 U/mL PLE) and varying substrate (**DCIP-R1**) concentrations between 2 and 50 μM in HEPES buffer (100 mM, pH = 8.0). The systems were excited at 480 nm and the emission kinetics was monitored at 37 °C for 30 min. A specific amount of the enzymes was chosen to allow an accurate measurement of the initial rate.

Under the abovementioned conditions, the substrate concentration, [S], is significantly higher than the enzyme concentration. As long as the substrate consumption is below 20%, the initial enzymatic velocity (v) can be approximated by the Henri–Michaelis–Menten equation v = (*v*_max_ × [S])/ ([S] + *K*_m_), where *v*_max_ is the maximal velocity at saturating substrate concentrations. This equation can be used to obtain the Michaelis–Menten curve by plotting the measured initial enzyme velocities against the corresponding substrate concentrations. The values for *K*_m_ and *v*_max_ were obtained by a nonlinear regression fit using OriginPro 2018. The initial velocity was calculated from the slope of the linear portion of each progress curve. The enzyme catalytic turnover number (*k*_cat_) was also obtained from the Michaelis–Menten plot. The *k*_cat_ value is defined as *k*_cat_ = *v*_max_ × [E_o_], where [E_o_] refers to the concentration of the enzyme used. The overall catalytic efficiency of the enzymatic reaction was calculated as follows:
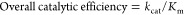


### LOD Calculation

The limit of detection (LOD) was calculated according to the formula LOD = 3σ*/k*, where σ is the standard deviation of the fluorescence intensity measurements for the blank sample and *k* refers to the slope of the linear curve between fluorescence enhancement versus the concentration of the enzymes (PLE and hCEs2).

### Inhibition Experiments

10 mM stock solution of 4-(2-aminoethyl) benzenesulfonyl fluoride (AEBSF) was prepared in Milli-Q water. For the spectroscopic studies, 0.5, 1, 1.5, and 2 mM concentrations of AEBSF were used in 1 mL of HEPES buffer (100 mM, pH = 8.0) with 10 μM of the probe (**DCIP-R1**) and 0.01 U/mL of PLE.

### Cell Culture and Imaging

The cell culture and cell experimental details including the cytotoxicity, colocalization, and inhibition assay are listed in the Supporting Information. For imaging, the **DCIP-R1** and **-R4** probes were dissolved in DMSO to reach a concentration of 1 mM. The cells were then incubated with the **DCIP-R1** and **-R4** probes at a final concentration of 1 μM for 20 min at 37 °C and washed with PBS twice before imaging. For continuous imaging from 0 to 30 min, HepG2 cells were imaged immediately post the addition of probes, without subsequent PBS washing. Cell images were acquired using a Nikon inverted multiphoton microscope (A1MP + Eclipse Ti-2E, Nikon Instrument Inc., Japan) with a water-immersed 40×, 1.15 NA objective.

### Animal Imaging

All animal experiments were conducted following protocols (UMARE0312021) approved by the Animal Ethics Committees, University of Macau. Six- to eight-week-old nude mice and Wistar rats were bred in the Animal Facility at the Faculty of Health Sciences. The detailed establishment of the liver tumor model is listed in the Supporting Information.

For *in vivo* imaging, 150 μL of 1 mM **DCIP-R1** and **-R4** probes dissolved in DMSO were injected into nude mice (BALB/c-nu, 6–8 weeks) *via* intraperitoneal or intratumoral injection. For tissue imaging, mice or rats were sacrificed for liver tissue/cancer collection. Fresh liver tissue/cancer samples were sectioned into 100-μm slices for the imaging of **DCIP-R1** and **-R4** probes. Fluorescence imaging was acquired at various time points using the AniView animal imaging system (BLT Photon Technology, China) with an excitation laser at 465 nm and an emission filter of 650–680 nm.

## Results and Discussion

### Design, Synthesis, and Characterization of **DCIP–OH** and **DCIP-R** Probes

We have proposed a substrate hydrolysis enzymatic activity (SHEA) design strategy for the detection of esterase enzymes under physiological conditions. To achieve this, a far-red–emitting intramolecular charge transfer (**ICT**) (E)-2-(3-(4-hydroxystyryl)-5,5-dimethylcyclohex-2-en-1-ylidene)malononitrile (**DCIP–OH**) was selected as the fluorophore. Furthermore, **DCIP–OH** was esterified using six different alkanoyl groups as the esterase recognition moieties for constructing the target esterase substrates **DCIP-R** (**R1–R6**). A methyl group was used for **-R1**, an ethyl group for **-R2**, a propyl group for **-R3**, a phenyl group for **-R4**, a 1-phenyl methyl group for **-R5**, and a 3-phenyl propyl group for **-R6**, with the detailed synthesis provided in Scheme S1. Distinct trigger groups were introduced to screen the probe with greater analytical performance such as high selectivity, sensitivity, and reactivity toward the esterases (PLE and hCEs2) *via* a fluorogenic response. All synthesized probes were purified using column chromatography and characterized using ^1^H and ^13^C[^1^H] NMR spectroscopy, as well as high-resolution mass spectrometry (HRMS) (Figures S1–S21).

### Investigating the Photophysical Properties of the Probes

Prior to the esterase detection studies, we inspected the preliminary photophysical properties of the chemosensors. The solvent-dependent emission spectra showed solvatochromic behavior, confirming the intramolecular charge transfer (ICT) property of **DCIP–OH** (Figure S22). The pH-dependent absorption and emission studies for **DCIP–OH** exhibited an equilibrium between the phenol and phenoxide species at pH 8.0 (Figure S23a–d). However, the more intense emission signal at 657 nm suggests phenoxide to be the dominant form in comparison to the phenol form at 582 nm (Figure S23e,f). Thus, we performed all the *in vitro* studies in a 100 mM HEPES buffer at pH 8.0. Moreover, this value is close to the physiological pH (7.4), and the esterase enzymes are known to be stable at this pH as their optimal activity lies between pH 7.5 and 8.5.^54,55^ The absorption and emission maxima of the esterase substrates are in the range of 392–404 and 552–572 nm, respectively (Figure S24). All the probes are found to be photostable upon excitation at 480 nm using a 450 W xenon lamp with a lamp intensity of approximately 100 lx (Figure S25). This crucial characteristic feature of the probes demonstrates their potential utility for prolonged (log-time) monitoring of bioanalytes under *in vivo* conditions.

### Screening the Best Recognition Group *via* Ester-Mediated Unmasking of **DCIP-R** Probes

**Figure 1:**
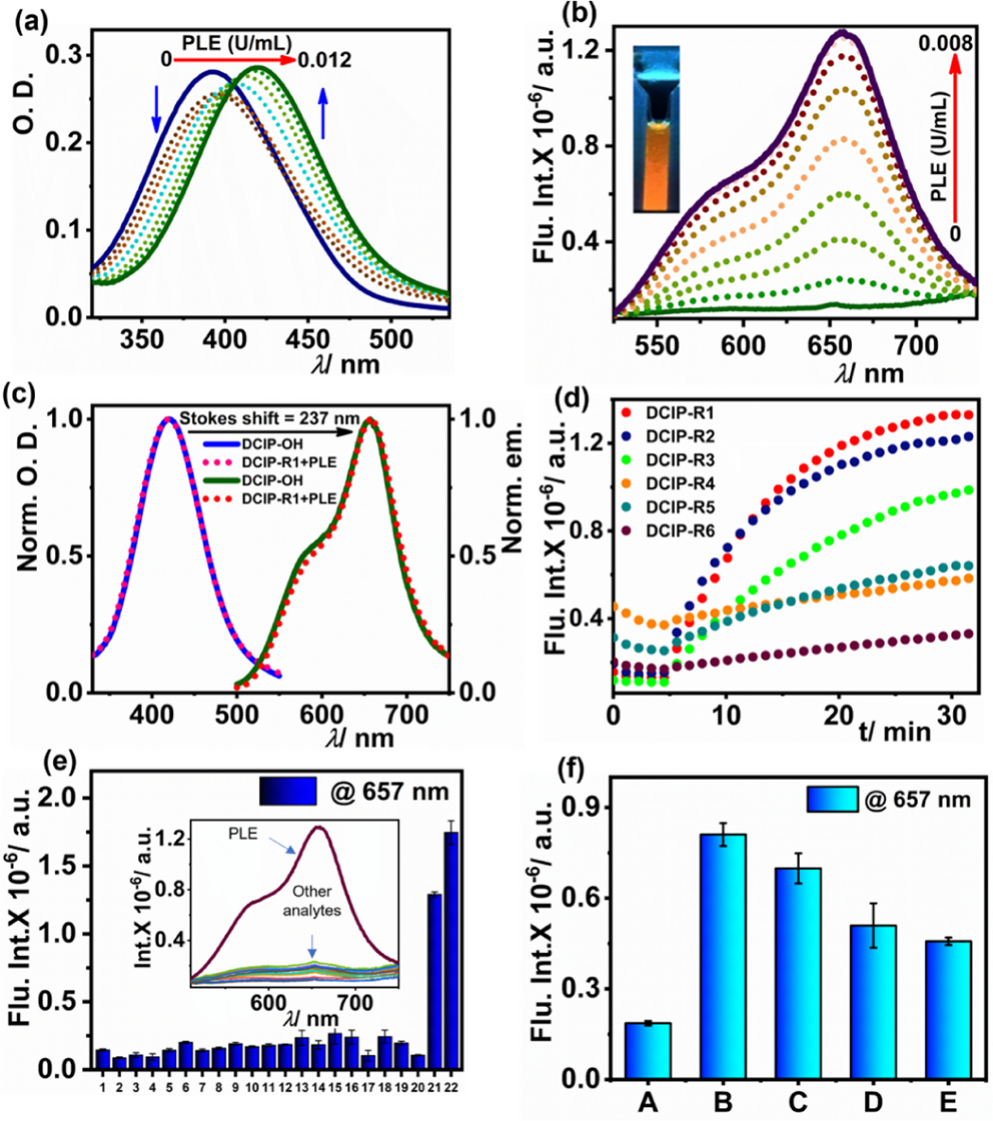
Characterization of DCIP probes in response to esterase activity. (a,b) Absorption and emission spectra of **DCIP-R1** with increasing concentration of PLE, respectively; (c) normalized absorption and emission spectra of **DCIP-R1 + PLE** and **DCIP–OH**; (d) kinetic studies of all substrates with 0.01 U/mL of PLE; (e) selectivity assay: 1. Blank, 2. NaCl (100 μM), 3. KCl (100 μM), 4. FeCl_3_ (100 μM,; 5. Glucose (100 μM), 6. Aspartic acid (100 μM), 7. Glycine (100 μM), 8. Lysine (100 μM), 9. Serine (100 μM), 10. Monosodium glutamate (100 μM), 11. Isoleucine (100 μM), 12. Urea (100 μM), 13. Trypsin (10 μg/mL), 14. Chymotrypsin (10 μg/mL), 15. RNase (10 μg/mL), 16. DNase (10 μg/mL), 17. Catalase (10 μg/mL), 18. HSA (10 μg/mL), 19. Glutathione (100 μM), 20. AChE (10 U/mL), 21. PLE (0.01 U/mL or 2.975 nM), and 22. hCEs2 (10 U/mL or 0.33 μM), and the inset shows the corresponding emission spectra; and (f) esterase activity inhibition assay with AEBSF, including (A) **DCIP-R1** only; (B) System A + 0.01 U/mL PLE; (C) System B + 0.5 mM AEBSF; (D) System B + 1.0 mM AEBSF; (E) System B + 1.5 mM AEBSF. All experiments were performed in HEPES buffer (100 mM; pH = 8.0) at 37 °C, λ_ex/_λ_em_ = 480 nm/657 nm, [**DCIP-R1**] = 10 μM.

Due to the strong substrate similarity between pig liver esterase (PLE) and human carboxylesterases (hCEs), PLE was initially used to assess the efficiency of **DCIP-R** probes for *in vitro* esterase detection and to determine the optimal “esterase-ester” combination.^56,57^ To begin with, we executed concentration-dependent steady-state absorption and emission titration experiments to comprehend the steric effects of the enzyme-cleavable groups on the catalytic proficiency of the esterase. The progressive addition of PLE gradually red shifted the absorption maxima of the **DCIP-R** probes to 420 nm, and emission studies revealed a fluorogenic response at 657 nm. Notably, the probes with alkyl chains as enzyme recognition moieties **DCIP-R1**, **-R2**, and **-R3** depicted a similar spectral modulation and attained their maximal value of emission at lower PLE concentrations (Figures S26 and S28). While substrates bearing phenyl substitution on the alkyl chains (**DCIP-R4**, **-R5**, and **-R6)** required higher units of enzymes to exhibit prominent enhancement in the emission spectra and to attain saturation (Figures S29 and S31). Although substrates with distinct trigger groups underwent selective hydrolysis with PLE, the acetyl moiety demonstrated the highest reactivity, making **DCIP-R1** an excellent substrate to detect esterase. Even with a few units of PLE (0–0.012 U/mL or 0–3.571 nM), **DCIP-R1** unveiled a ratiometric modulation *via* a gradual bathochromic shift in the absorption maxima from 392 to 420 nm with an isosbestic point at 405 nm (Figures 1a and S26a). Likewise, the reaction with PLE (0–0.008 U/mL or 0–2.380 nM) rendered a significant enhancement in fluorescence intensity with an emission peak majored at 657 nm and a shoulder peak at 582 nm (Figure 1b). Consequently, the emission color of the solution changed from colorless to orange under UV light owing to the formation of the hydrolysis product **DCIP–OH** (Figure 1b inset). Additionally, Figure 1c shows that the characteristic spectral modulation in **DCIP-R1** roughly corresponds with the absorbance and emission maxima of the fluorescence product **DCIP–OH**, indicating that **DCIP-R1** has undergone ester bond breaking mediated by PLE. A large Stokes shift of 237 nm has been observed, which can be exploited to detect and monitor endogenous esterase in live cells and animal models. In contrast, the probe **DCIP-R4** lacks a discernible ratiometric response in absorption spectra and requires higher concentrations of PLE to accomplish a turn-on response in emission spectra (Figure S29). The distinct enhancement in the emission intensity of **DCIP-R** probes at 657 nm is shown in Figure S26b. Moreover, gauging the suitable recognition group for activating hCEs is extremely imperative as their abnormalities indicate the status of liver conditions. Therefore, we used human carboxylesterase 2 (hCEs2) as a representative of human esterase enzymes to perform steady-state absorption and emission titration studies with **DCIP-R1**. hCEs2-aided hydrolysis of **DCIP-R1** displayed a remarkable reduction and concomitant increment in the absorbance at 392 and 426 nm, respectively, using 0–18 U/mL (0–0.599 μM) of hCEs2 (Figure S26c,d). As envisaged, the appearance of amplified emission at 657 nm further supported that hCEs2 (0–11 U/mL or 0–0.366 μM) triggered cleavage of the ester bond (Figure S26e,f). Thus, scanning a wide variety of esterase substrates identified **DCIP-R1** as a promising esterase probe, contributing to the formation of an ideal “esterase-ester” combination.

### Time-Dependent Spectroscopic Analysis

Promising results obtained from the steady-state measurements encouraged us to perform time-dependent fluorescence studies with 0.01 U/mL of PLE and 10 U/mL of hCEs2. The kinetic profile of substrates **DCIP-R1**, **-R2**, and **-R3** showed gradual hydrolysis, reaching maximum fluorescence intensity at 15–25 min. However, probes **-R4**, **-R5,** and **-R6** undergo slow hydrolysis, resulting in weak emission at 657 nm when excited at 480 nm (Figures 1d and S26g). Thus, kinetic studies revealed the ability of the probe **DCIP-R1** to exhibit maximum response, with enzymatic activity becoming saturated within the shortest time frame. Therefore, we have chosen **DCIP-R1** to perform PLE (0.001–0.008 U/mL) and hCEs2 (0–15 U/mL) concentration-dependent emission kinetics under physiological conditions. The rate of ester hydrolysis increased with an increase in the amount of esterase added, with the entire enzymatic reaction completing within 20 min for PLE (0.008 U/mL) and 15 min for hCEs2 (15 U/mL) (Figures S26h,i). Additionally, enzyme kinetic assays were performed to quantify the catalytic efficiency of PLE against **DCIP-R1**. This was performed by monitoring the change in enzyme kinetics using different concentrations of the substrate (**DCIP-R1**). Figure S26j displays the Michaelis–Menten curve for PLE. Accordingly, *K*_m_, *k*_cat_, and *k*_cat_/*K*_m_ values were determined and found to be 2.95 μM, 4.73 s^–1^, and 1.6 × 10^6^ M^–1^ s^–1^, respectively. This indicates a fast enzymatic reaction with high catalytic efficiency of PLE to convert **DCIP-R1** into **–OH**. Consequently, the findings from the time-dependent spectroscopy revealed good agreement with the steady-state investigations. Notably, the hydrolysis efficiency of the probes **DCIP-R4**, **-R5**, and **-R6** was found to be diminished because of the phenyl ring’s enhanced steric effect. Accordingly, we hypothesized that substrate-induced conformational changes at the enzyme’s catalytic triad might account for the varying degrees of substrate hydrolysis.^58^

### LOD and Selectivity

The linear dependence of fluorescence intensity with a lower concentration of esterase (PLE or hCEs2) was utilized to estimate the limit of detection (LOD). LOD was calculated using the formula 3σ/*k* and was found to be 47 pM (0.63 mU/mL) and 6 nM (0.17 U/mL) for **DCIP-R1** with PLE and hCEs2, respectively (Figure S26k,l). The remaining alkyl- and phenyl-substituted probes exhibited LOD in the pM and nM range, respectively, with PLE (Figures S27e–S31e).

To examine the interference from biologically significant analytes, reactions of **DCIP-R1** with common inorganic salts such as NaCl, KCl, and FeCl_3_; hydrolyzing enzymes such as trypsin, chymotrypsin, DNase, RNase, catalase, and acetylcholine esterase; amino acids such as glycine, lysine, serine, aspartic acid, glutamic acid (monosodium glutamate), and isoleucine; and metabolites such as glucose, urea, glutathione, and HSA were performed. As depicted in Figure 1e, the turn-on response of **DCIP-R1** was observed only with esterases (PLE, hCEs2), highlighting the selectivity of the probe for the esterase enzymes and its potential application for *in vivo* esterase detection in the complex biological milieu. Furthermore, to validate that the turn-on response was initiated *via* esterase-mediated cleavage of the ester bond, an esterase inhibitor AEBSF was introduced into the analysis system.^59^ On increasing the concentration of the inhibitor, there was a remarkable reduction in the emission intensity at 657 nm. This observation confirms that the fluorogenic response is indeed a result of esterase-mediated hydrolysis of the substrate at the catalytic triad (Figure 1f).

### Plausible Detection Mechanism and Confirmation of Ester Hydrolysis

Hydrolysis of the ester substrate is usually conducted using a classic base-catalyzed two-step mechanism that is conserved in all serine hydrolases (Scheme S2).^54^ This process depends on an essential catalytic triad that is situated at the active site in mammalian carboxylesterases and is generally composed of three amino acid residues (serine, histidine, and glutamate). The catalytic mechanism involves a combined nucleophilic attack by the triad on electrophilic ester substrates that results in the generation of carboxylic acid and alcohol generation. Based on the spectral analysis, it was speculated that the probes undergo esterase-mediated hydrolysis to produce the fluorescent product **DCIP–OH**. To confirm this inference, HRMS and HPLC studies were conducted. From Figure S32a, it can be observed that only **DCIP-R1** shows a peak at *m*/*z* 355.1417 [M + Na] ^+^ while after reaction with PLE, the mixture shows a peak at *m*/*z* 313.2762 [M + Na] ^+^ (Figure S32b), corresponding to the peak shown by the compound **DCIP–OH** (Figure S32c). In HPLC studies, **DCIP-R1**, and **–OH** exhibited chromatographic peaks with retention time at 2.58 and 2.01 min, respectively. After this, a mixture of **DCIP-R1** and PLE was injected 5 times at an interval of 4 min each. As time progressed, the chromatographic peak at 2.58 min gradually reduced, while the one at 2.01 min continued to increase (Figure S33a). This suggested the depletion of **DCIP-R1** with time as it got converted into **DCIP–OH** in the presence of PLE. On plotting the peak area for the two different chromatographic peaks against time, we obtained consistent growth and decay curves for **DCIP–OH** and **-R1**, respectively (Figure S33b). Complete hydrolysis of ester moiety occurred at 16 min, which is comparable with the fluorescence kinetic studies. Similar results were obtained for probes **DCIP-R2** and **-R3** with slightly slower kinetic profiles by completing the ester hydrolysis process at 18 and 28 min, respectively (Figures S34 and S35). On the contrary, **DCIP-R4**, **-R5**, and **-R6** took more than 40 min to release the product **DCIP–OH** (Figures S36–S38). Therefore, a systematic *in vitro* study suggested that substrates with alkyl-trigger groups outperformed phenyl-substituted trigger groups, allowing an *in vivo* comparison study using **DCIP-R1** and **-R4**.

### Detection of Esterase Using **DCIP-R1** and **-R4** in Living Cells

**Figure 2:**
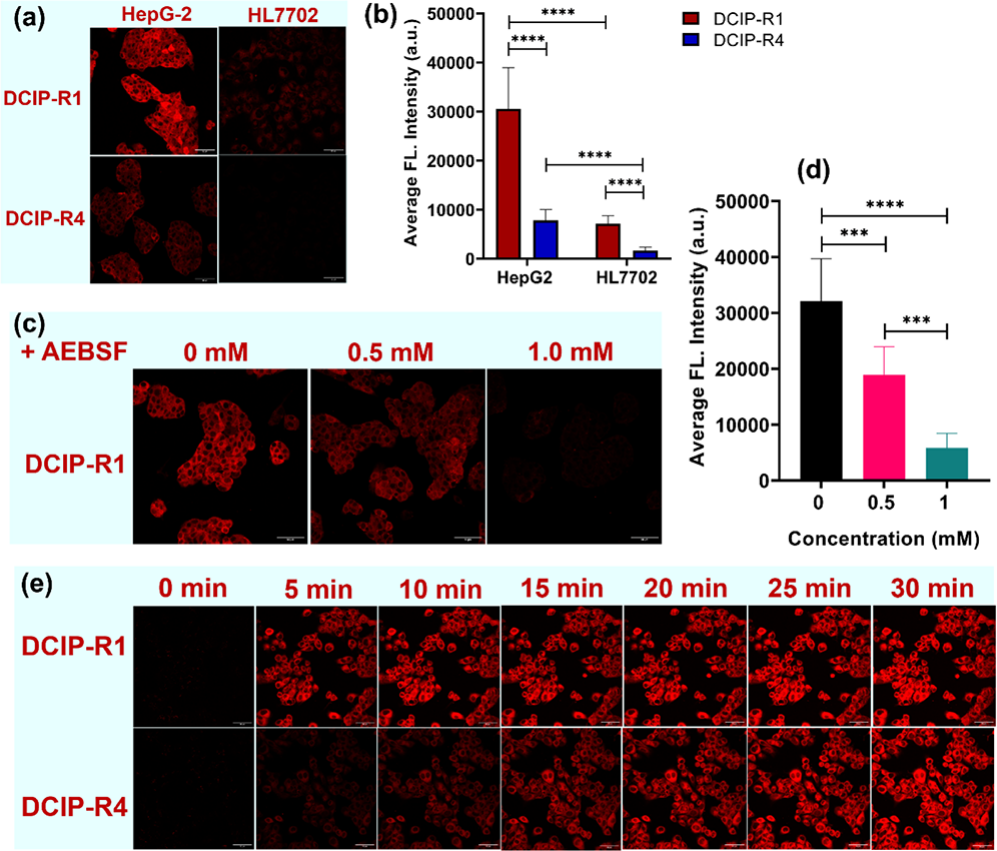
Two-photon fluorescence imaging of esterase in liver cancer cells by **DCIP-R** probes. (a) Representative fluorescence images of HepG2 or HL7702 cells, which were incubated with **DCIP-R1** or **-R4** for 10 min, respectively; (b) quantification of the average fluorescence intensity obtained from (a); (c) representative fluorescence images of HepG2 pretreated with AEBSF at various concentrations (0, 0.5, and 1 mM) for 30 min before staining with **DCIP-R1**; (d) quantification of average fluorescence intensity obtained from (c); (e) fluorescence images of HepG2 cells stained with 1 μM of **DCIP-R1** (upper panel) and **-R4** (lower panel) from 0 to 30 min at 37 °C. Cell imaging was performed by two-photon microscopy: λ_ex_ = 960 nm, λ_em_ = 604–678 nm for the red channel. Scale bars: 50 μm. Data are presented as mean ± SD. Significant differences were analyzed using the Kruskal–Wallis test with the Dunn’s multiple comparison test. ****p < 0.0001.

Having demonstrated excellent *in vitro* detection of esterase under physiological conditions, we explored cellular tracking and imaging of endogenous esterase using probes **DCIP-R1** and **-R4** as representative of the alkyl- and phenyl-bearing recognition moieties. Live cell imaging studies were performed with human liver cancer cell line HepG2, which is well-known for the overexpression of esterase.^57^ The human normal liver cell line HL7702, which expresses low levels of esterase, was used as a control.^46^ To start with, the cytotoxicity of the probes was evaluated in HepG2 cells using a cell counting kit-8 (CCK-8) assay (Figure S39). Cell viability remained at approximately 80% with **DCIP-R1** at concentrations up to 50 μM. Therefore, we employed 1 μM of the probes to perform live cell imaging. Using single-photon fluorescence microscopy with an excitation wavelength of 488 nm, strong fluorescence was observed in the green (500–550 nm) and red (570–620 nm) channels (Figure S40). Both **DCIP-R** probes were found to locate mostly in the cytoplasm, with no nuclear uptake observed (Figure S41). As dicyanoisophorone derivatives exhibit two-photon activity, we performed two-photon fluorescence microscopy using an excitation wavelength of 960 nm.^45^ Red fluorescence was observed in HepG2 cells following a 10-min incubation period with either the **DCIP-R1** or **-R4** probe (Figure 2a). Notably, the fluorescence intensity of **DCIP-R1** was significantly higher than that of **-R4**.

This observation underscores the potential application of these probes in multiphoton fluorescence microscopy, with **DCIP-R1** exhibiting superior performance in cell imaging. A similar pattern, albeit with a weaker fluorescence signal, was observed in HL7702 cells (Figure 2a,b). The comparison between HepG2 and HL7702 cells implies that **DCIP-R** probes are capable of revealing the actual expression level of esterase within the cells. An inhibitory assay using AEBSF was also employed. We can see that the addition of AEBSF led to a significant reduction in fluorescence intensity (Figure 2c,d). These findings suggest that the fluorescence activity is dependent on endogenous esterase-mediated hydrolysis of the **DCIP-R** probes. Moreover, the fluorescence signal of **DCIP-R1** and **-R4** probes within HepG2 cells exhibited a time-dependent increase (Figure 2e), indicating the potential use of these probes for real-time monitoring of the changes in esterase levels.

### Monitoring and Imaging of Esterase in Nude Mice

Encouraged by the performance of the probes in liver cancer cells, we investigated the capability of **DCIP-R1** and **-R4** to detect and monitor enzyme activity under complex biological models (Figure S42a). The fluorescence intensity in mice receiving **DCIP-R1** showed an increasing trend, reaching maximum fluorescence at 20 min post injection (Figure S42b). The change in fluorescence signal of **DCIP-R4** showed a similar pattern, although the intensity was weaker in comparison with that of **-R1** (Figure S42c). Quantification of *in vivo* fluorescence intensity indicates that **DCIP-R1** can be efficiently hydrolyzed to the fluorescence product **DCIP–OH**, in contrast to **-R4** (Figure S42d). The faster *in vivo* kinetic response of **DCIP-R1** shows good agreement with *in vitro* investigations. Thus, increasing the complexity of biological milieu does not alter the reactivity of the probe toward endogenous esterase. We subsequently dissected and imaged mouse organs, including the liver, lung, spleen, kidney, and heart, 50 min post IP injection of the **DCIP-R1** probe. Only the liver exhibited fluorescence, indicating that esterase expression is primarily localized in the liver (Figure S43).

### Imaging of Endogenous Esterase Activity in DEN-Induced Rat Liver Tumor

**Figure 3:**
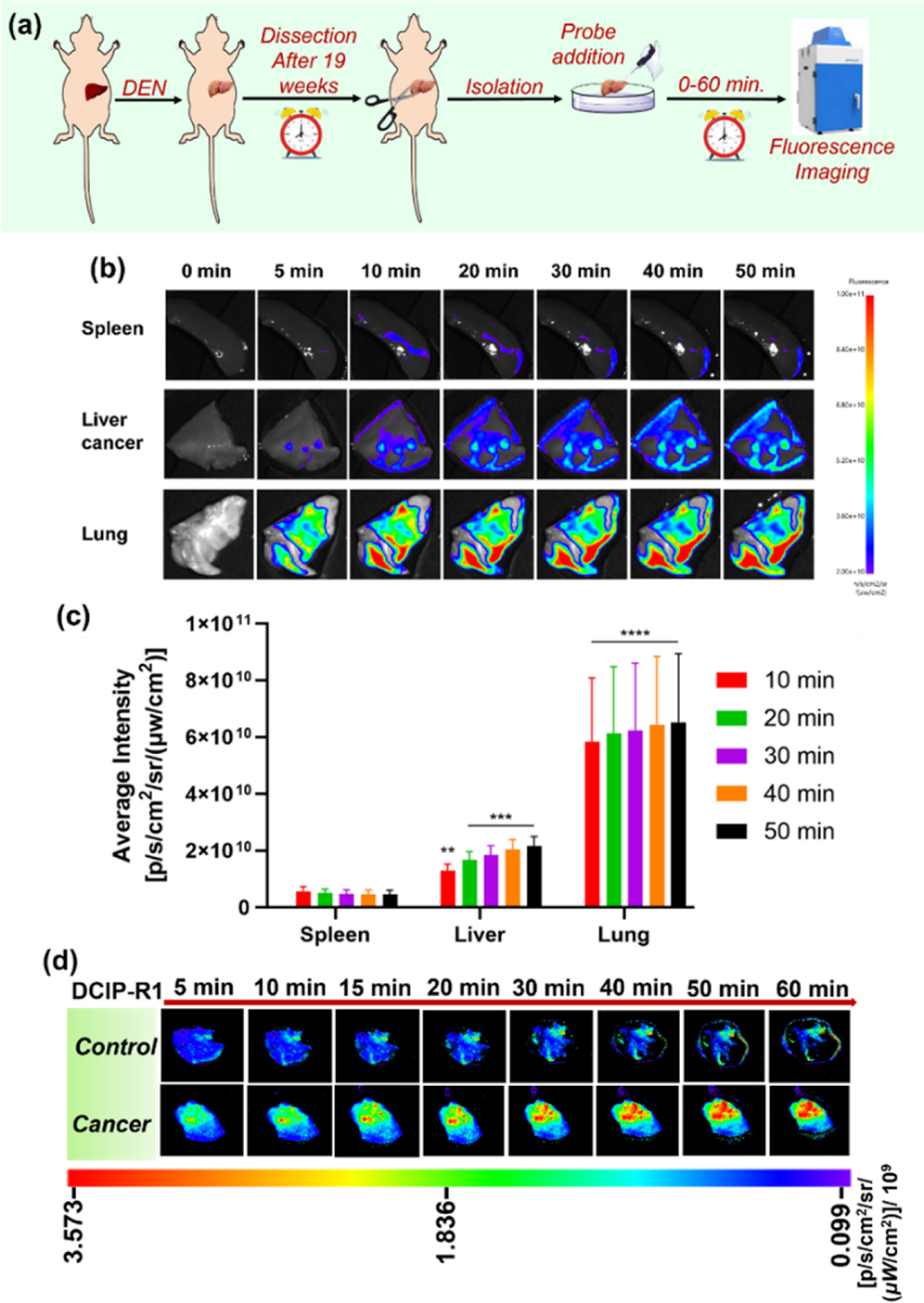
Time-dependent fluorescence imaging of DEN-induced rat liver tumor model dissected after 19 weeks. (a) The procedure for evaluating the functionality of probes in detecting liver cancers; (b) fluorescence imaging of rat liver tumor, lung, and spleen treated with **DCIP-R1**; (c) quantification of fluorescence intensity obtained from (b); and (d) fluorescence imaging of rat liver tumor and healthy liver (control) treated with **DCIP-R1**. Data are presented as mean ± SD. Significant differences were analyzed using two-way ANOVA with the Sidak’s multiple comparison test. **p < 0.01; ***p < 0.001; and ****p < 0.0001.

An increase in the human esterase levels has been found in the development of hepatocellular carcinoma. In our study, we employed the rat model of diethylnitrosamine (DEN)–induced liver cancer, which closely mimics human liver cancer development (Figure 3a).^60−62^ After **DCIP-R1** administration, a stronger fluorescent signal was seen in the liver tumor, compared to the spleen (Figure 3b). There were significant differences in fluorescence intensity between these two tissues starting from 10 min post treatment (**p < 0.01, ****p < 0.0001) (Figure 3c). Furthermore, liver cancer tissues exhibited a stronger fluorescence signal compared with healthy liver tissues when using the **DCIP-R1** probe (Figures 3d and S44). The activity of carboxylesterase in the liver of both the DEN-induced liver tumor group and the control group was also evaluated using a commercial kit. The results showed a significant increase in carboxylesterase levels, specifically within liver tumors (Figure S45). These results confirm the overexpression of esterase in liver cancer. Interestingly, the fluorescence signal in the lung was significantly higher than that in liver tumors and spleen (****p < 0.0001) (Figure 3b,c), suggesting elevated esterase activity in lung tissues. This could be because DEN induced not only primary liver cancer but also lung metastasis, as reported in previous studies.^63−65^ This finding supports the evidence linking increased esterase levels to both primary tumorigenesis and metastasis. In summary, our findings suggest that the **DCIP-R1** probe has the ability to diagnose primary liver tumors and lung metastasis through esterase activity assessment.

### Imaging of Endogenous Esterase Activity in Xenografted HepG2 Liver Tumor

**Figure 4:**
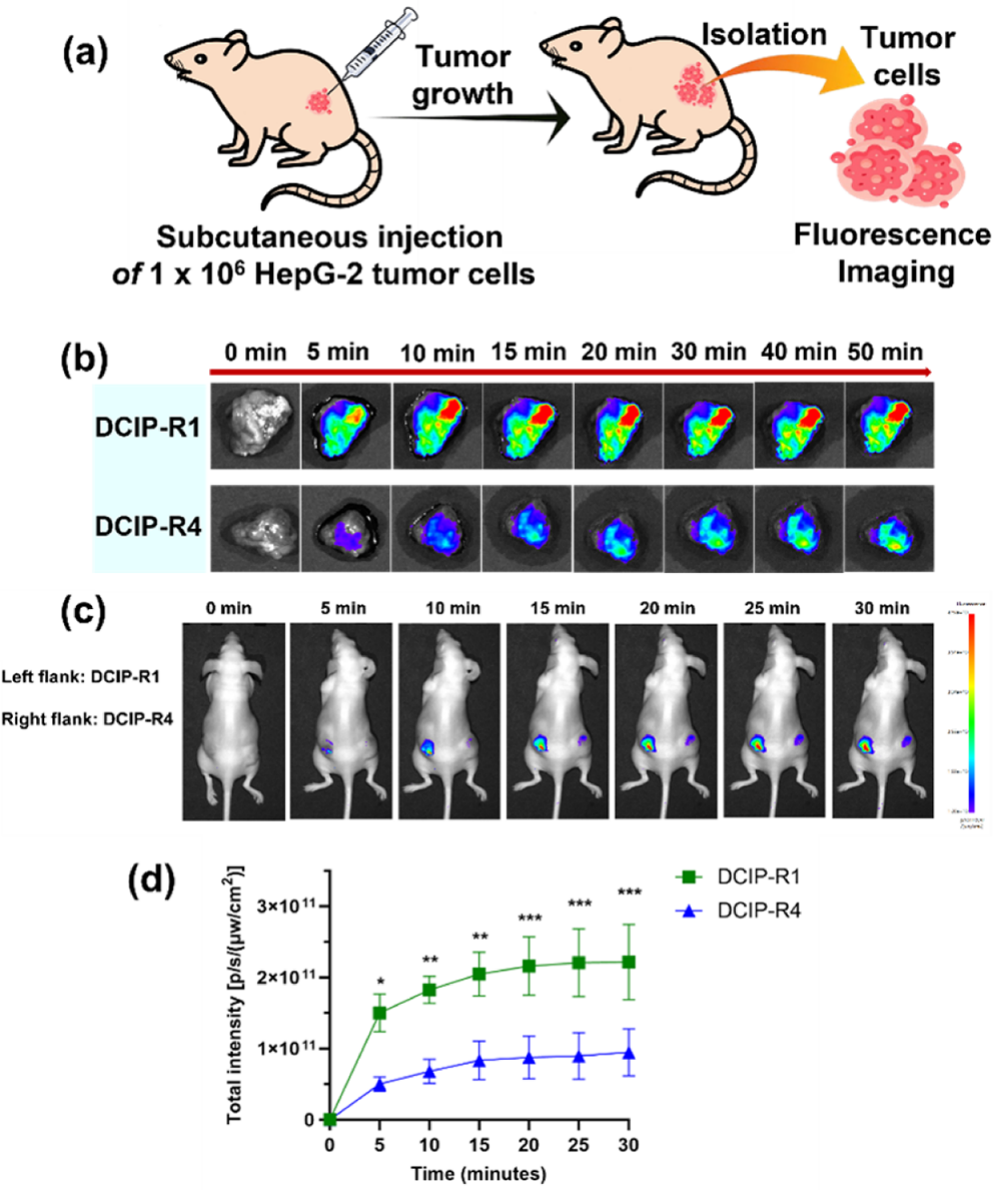
Fluorescence imaging of endogenous esterase activity in xenografted HepG2 liver tumor. (a) Scheme showing the establishment of HepG2 liver tumor model, (b) fluorescence imaging of mouse HepG2 liver tumor slices using **DCIP-R1** (upper panel) and **-R4** (lower panel), (c) *in vivo* fluorescence imaging of esterase activity in tumor-bearing mice was performed by intratumor injection of **DCIP-R1** and **DCIP-R4** (1 mM) into the left and right flanks, respectively; and (d) quantification of fluorescence intensity of tumor region obtained from (c). Data are presented as mean ± SD. Significant differences were analyzed using two-way ANOVA with the Sidak’s multiple comparison test. *p < 0.05; **p < 0.01; and ***p < 0.001.

A xenograft model of liver tumor was also employed to assess human esterase activity, which was established by subcutaneous injection of HepG2 tumor cells into the left and right flanks of nude mice (Figure 4a). **DCIP-R1** showed a stronger fluorescence signal compared to **-R4**, indicating superior performance in detecting endogenous esterase activity within tumors (Figures 4b and S46). The fluorescence intensity of **DCIP-R1** rapidly increased upon reaction with endogenous esterase and reached a maximum value within 20 min (Figure S47). Moreover, *in vivo* imaging of **DCIP-R1** and **-R4** was conducted through intratumoral injection into mice bearing subcutaneous HepG2 tumors. Obvious fluorescence was observed in mice treated with **DCIP-R1** as early as 5 min post injection, whereas the fluorescence in mice treated with **DCIP-R4** was weak over time (Figure 4c,d). These *in vivo* results indicate that **DCIP-R1** possesses the capability to track CE activity within liver tumors *in vivo*. Therefore, dicyanoisophorones exhibit promising potential as discerning indicators for esterase enzymes, applicable under both *in vitro* and *in vivo* conditions compared to some reported fluorogenic probes (Figure S48).

## Conclusions

In this study, we developed dicyanoisophorone-based fluorogenic ester substrates (**DCIP-R**) to efficiently detect esterase activity at the nanomolar range, using a “SHEA” design strategy. Our findings showed that the addition of esterase shifted the absorption maxima of the probes to 420 nm and produced a turn-on emission response at 657 nm. Esterase-mediated hydrolysis led to the formation of the two-photon active luminous product **DCIP–OH**, verified by HRMS and HPLC analyses. *In vitro* and *in vivo* experiments confirmed the effectiveness of our probes, particularly **DCIP-R1**, in tracking esterase activity in real time within HCC. These results suggest that two-photon active dicyanoisophorone derivatives have significant potential for disease diagnosis, drug formulation, and pharmacological applications.

## Supporting Information Available

Details of synthesis, characterization of molecules (^1^H, ^13^C[^1^H], and HRMS), preliminary photophysical properties, spectroscopic investigations with PLE and hCEs2, HPLC analysis, live cell imaging, tumor liver slice imaging, and *in vivo* imaging (PDF)
