# Supplementary material for: Activity-Based Dicyanoisophorone Derivatives: Fluorogenic Toolbox Enables Direct Visualization and Monitoring of Esterase Activity in Tumor Models
Source: Anal Chem. 2024 Nov 1;96(45):18278–86. doi: 10.1021/acs.analchem.4c04721 (PMC11561878; doi:10.1021/acs.analchem.4c04721)
Supplement: Supplementary file 1 — ac4c04721_si_001.pdf [file ac4c04721_si_001.pdf]

Supporting information

Activity-based Dicyanoisophorone Derivatives: Fluorogenic Toolbox Enables Direct Visualization and Monitoring of Esterase Activity in Tumor Models

Kavyashree P.,[1] Atri Bhattacharya,[1,2,#] Lidong Du,[3,#] Akshay Silswal,[1] Moxin Li,[3] Jiayue Cao,[3] Qingqing Zhou,[3] Weiming Zheng,[4,*] Tzu-Ming Liu,[3,*] and Apurba Lal Koner[1,*]

[1] Bionanotechnology Lab, Department of Chemistry, Indian Institute of Science Education and Research Bhopal, Bhopal Bypass Road, Bhauri, Bhopal 462066, Madhya Pradesh, India

[2] Department of Chemistry, University of Texas at Austin, Austin, Texas 78712-1224, United States of America

[3] Institute of Translational Medicine, Faculty of Health Sciences & Ministry of Education Frontiers Science Center for Precision Oncology, University of Macau, Taipa, Macau 999078, China

[4] Translational Medicine R&D Center, Zhuhai UM Science and Technology Research Institute, Zhuhai 519000, China

* Corresponding author

Weiming Zheng, E-mail: jeremy.zhengwm@gmail.com;

Tzu-Ming Liu, E-mail: tmliu@um.edu.mo;

Apurba Lal Koner, E-mail: akoner@iiserb.ac.in

[#] These authors contributed equally to this work

Table of Content

1. Experimental Section

1.1 Materials……………………………………………………………….5

1.2 Methods and Measurements

1.2.1 Nuclear Magnetic Resonance (NMR) and Mass Spectrometry………………………5

1.2.2 Steady-state Spectroscopic Measurements…………………………………………..6

1.2.3 Preparation of Stock Solutions………………………………………………………6

1.2.4 Concentration-dependent Kinetics…………………………………………………..6

1.2.5 Enzyme Kinetics Assays……………………………………………………………..7

1.2.6 LOD Calculations……………………………………………………………………7

1.2.7 HPLC Studies………………………………………………………………………..7-8

1.2.7 Selectivity Assay…………………………………………………………………… 8

1.2.8 Inhibition Experiments………………………………………………………………8

2. Live Cell Imaging and *in vivo* Studies

2.1 Cell Imaging

2.1.1 Cell Culture Method…………………………………………………………………..8-9

2.1.2 Cytotoxicity Assay…………………………………………………………………...9

2.1.3 Fluorescence Imaging of the Endogenous Esterase in Liver Cancer Cells……….9-10

2.2 Animal Imaging

2.2.1 Imaging of **DCIP-R** Probes in Nude Mice…………………………………………10

2.2.2 Establishment of a Rat Model of Diethylnitrosamine (DEN)-induced HCC……….10

2.2.3 Establishment of HepG2 Liver Tumor Model…………………………………..10-11

3. Design and Synthesis of DCIP-OH and DCIP-R Probes

Scheme S1: Synthetic Scheme for **DCIP-OH** and **DCIP-R** Probes……………………...11

3.1 Synthesis of Compound **DCP-OH**……………………………………………………11

3.2 Synthesis of Compound **DCIP-R1**…………………………………………… .11-12

3.3 Synthesis of Compound **DCIP-R2**…………………………………………………..11-12

3.4 Synthesis of Compound **DCIP-R3**…………………………………………………..11-12

3.5 Synthesis of Compound **DCIP-R4**…………………………………………………..11-13

3.6 Synthesis of Compound **DCIP-R5**…………………………………………………..11-13

3.7 Synthesis of Compound **DCIP-R6**……………………………………………………13

4. NMR and Mass Data

4.1 Figure S1, S2, & S3: ^{1}H, ^{13}C[^{1}H] NMR, and HRMS Spectrum of **DCIP-OH**……14-15

4.2 Figure S4, S5, & S6: ^{1}H, ^{13}C[^{1}H] NMR, and HRMS Spectrum of **DCIP-R1**…….15-16

4.3 Figure S7, S8, & S9: ^{1}H, ^{13}C[^{1}H] NMR, and HRMS Spectrum of **DCIP-R2**…….17-18

4.4 Figure S10, S11, & S12: ^{1}H, ^{13}C[^{1}H] NMR, and HRMS Spectrum of **DCIP-R3** 18-19

4.5 Figure S13, S14, & S15: ^{1}H, ^{13}C[^{1}H] NMR, and HRMS Spectrum of **DCIP-R4** 20-21

4.6 Figure S16, S17, & S18: ^{1}H, ^{13}C[^{1}H] NMR, and HRMS Spectrum of **DCIP-R5** 21-22

4.7 Figure S19, S20, & S21: ^{1}H, ^{13}C[^{1}H] NMR, and HRMS Spectrum of **DCIP-R6** 23-24

5. Photophysical Properties of the Probes

5.1 Figure S22: Solvent-dependent Emission for **DCIP-OH**…………………………… 24

5.2 Figure S23: pH-dependent Abs. and Emission for **DCIP-OH**………………………..25

5.3 Figure S24: Abs. and Emission Spectra of **DCIP-R** Probes in HEPES Buffer………25

5.4 Figure S25: Photostability of **DCIP-R** Probes in HEPES Buffer……………………26

6. Interaction of Probes with Esterase

6.1 Figure S26: UV-vis. Absorption, Emission, and LOD for **DCIP-R1** with Esterase….27

6.2 Figure S27: UV-vis. Absorption, Emission, and LOD for **DCIP-R2** with Esterase….28

6.3 Figure S28: UV-vis. Absorption, Emission, and LOD for **DCIP-R3** with Esterase….29

6.4 Figure S29: UV-vis. Absorption, Emission, and LOD for **DCIP-R4** with Esterase….30

6.5 Figure S30: UV-vis. Absorption, Emission, and LOD for **DCIP-R5** with Esterase….31

6.6 Figure S31: UV-vis. Absorption, Emission, and LOD for **DCIP-R6** with Esterase....32

7. Scheme S1: The mechanism for esterase-catalyzed hydrolysis of an ester substrate..33

8. Figure S32: HRMS of **DCIP-R1** with PLE…………………...………………………..34

9. HPLC Analysis of Probes with PLE

9.1 Figure S33: HPLC Profile of **DCIP-R1** Catalyzed by PLE………………………..35

9.2 Figure S34: HPLC Profile of **DCIP-R2** Catalyzed by PLE…………………………35

9.3 Figure S35: HPLC Profile of **DCIP-R3** Catalyzed by PLE…………………………36

9.4 Figure S36: HPLC Profile of **DCIP-R4** Catalyzed by PLE…………………………36

9.5. Figure S37: HPLC Profile of **DCIP-R5** Catalyzed by PLE…………………………37

9.5. Figure S38: HPLC Profile of **DCIP-R6** Catalyzed by PLE…………………………37

10. Live Cell Imaging Studies

10.1 Figure S39: CCK-8 assay in HepG2 cells…………………………………………...38

10.2 Figure S40: Live cell imaging in HepG2 cells………………………………………38

10.3 Figure S41: Colocalization studies in HepG2 cells…………………………………39

11. *In vivo* Imaging Studies

11.2 Figure S42: *In vivo* imaging of **DCIP-R1** & **-R4** in healthy nude mice ……………39

11.3 Figure S43: Fluorescence imaging of mouse organs with **DCIP-R1**………………..40

11.3 Figure S44: Fluorescence imaging of rat liver tumor slices treated with probes……40

11.3 Figure S45: Measurement of CEs levels in healthy liver, spleen & liver tumors, using a commercial kit……………………………………...……………………………...41

11.4 Figure S46: Fluorescence imaging of mouse HepG2 liver tumor slices……………41

11.5 Figure S47: Quantification of fluorescence intensity………………………………..42

12. Comparison of Literature Reports with Our Probe

12.1 Figure S48**:** Some reported fluorogenic probes for sensing esterase activity……42-43

References……………………………………………………………………………...43-44

1. Experimental section:

1.1 Materials

All the chemical reagents and solvents were acquired from commercial sources and used as received without further purification. To prepare the buffer solution, 4-(2-hydroxyethyl)-1-piperazineethanesulfonic acid (HEPES) was purchased from Otto Chemie Pvt. Ltd. (India). Sodium hydroxide (NaOH) and sodium chloride (NaCl) were obtained from SD Fine Chem. Ltd. Potassium chloride (KCl) and potassium dihydrogen phosphate (KH_2PO_4) were purchased from Central Drug House (P) Ltd. Sodium hydrogen phosphate (Na_2HPO_4) was taken from Sisco Research Laboratories. Spectroscopic grade solvents including, acetonitrile (ACN), dichloromethane (DCM), dimethyl sulfoxide (DMSO), dimethyl formamide (DMF), 1,4-Dioxane, methanol (MeOH), and tetrahydrofuran (THF) were purchased from Sisco Research Laboratories. Spectroscopic grade ethylene glycol (EG) was purchased from Sigma-Aldrich. Acetylcholine esterase (AChE) was obtained from MedChemExpress. Both porcine liver esterase (PLE) and human carboxylesterase 2 (hCEs2) enzymes were procured from Sigma-Aldrich. Ferric chloride ($FeCl_3$) was purchased from Sigma-Aldrich. Analytes like glucose and urea were acquired from Vetec and Finar, respectively. Amino acids like L-serine and L-aspartic acid were taken from Sisco Research Laboratories; L-Lysine hydrochloride and glycine were obtained from Otto Chemie Pvt. Ltd. (India). L-glutathione was obtained from Sigma-Aldrich. Enzymes such as trypsin, chymotrypsin, ribonuclease (RNase), deoxyribonuclease (DNase), and catalase (from bovine serum), and the protein human serum albumin (HSA) were purchased from HiMedia (USA). The esterase inhibitor, 4-(2-aminoethyl) benzene sulfonyl fluoride (AEBSF) was purchased from Tokyo Chemical Industry (India) Pvt. Ltd.

1.2 Methods and Measurements

1.2.1. Nuclear Magnetic Resonance (NMR) and Mass Spectrometry

^{1}H NMR spectra were recorded on Bruker's AVANCE-III 500 MHz spectrometer. The data was processed using the MestReNova 14.1.2 software. Chemical shifts (δ) were reported in ppm relative to solvent residual signals (^{1}H: δ = 7.26 for $CDCl_3$). ^{1}H NMR data are reported in the following order: chemical shift (δ) in ppm downfield from tetramethylsilane as the internal reference, multiplicity (s-singlet, d-doublet, dd- doublet of the doublet, t-triplet, m-multiplet), approximate coupling constants (J) in Hertz (Hz) and the number of protons. Electrospray

ionization (ESI) data were recorded on Bruker MicroTOF QII high-resolution mass spectrometer using either chloroform or methanol as solvent.

1.2.2. Steady-state Spectroscopic Measurements

All the steady-state absorption measurements were performed with SPECORD 210 Plus UV Vis. spectrophotometer from Analytik Jena using the software ASpect UV 1.2.0. software. All the steady-state and kinetic emission measurements were carried out with HORIBA Jobin Yvon Fluorolog fluorimeter using FluorEssence V3.9 software. The temperature of the measurements was adjusted to 37 °C using the Quantum Northwest TC 1 temperature controller and T-App program. To simulate the physiological conditions, measurements were carried out at 37 °C with the aid of the JUMO dTRON 308 temperature controller. Steady-state absorption spectra and emission spectra were recorded using 1.7 mL quartz cuvettes of 1 cm path length. Emission was recorded in the region from 500 nm to 750 nm by exciting at 480 nm, keeping both excitation and emission slit widths 5 nm, with an integration time of 1.5 seconds. pH-dependent studies were performed by adjusting the pH of the HEPES buffer between 3.0 and 11.0 with 10 (N) NaOH. All the steady-state spectroscopic measurements were plotted using OriginPro 2018.

1.2.3. Sample Preparations

1 mM stock solutions of **DCIP-OH** and **DCIP-R1** to **R6** were prepared in spectroscopic grade acetonitrile. For all the absorption and emission studies, the probe solutions were diluted to 10 μM in HEPES buffer solution (100 mM, pH = 8.0). 1.65 U/mL stock solutions of PLE were used for all the studies and the stock solution was prepared by dissolving 0.33 mg of PLE in 4 mL of PBS buffer solution (10 mM, pH = 7.46). 200 U/mL stock solution of hCEs2 was used for the spectroscopic studies and this was prepared by dissolving 40 μL of hCEs2 in 460 μL of PBS buffer solution (10 mM, pH = 7.46).

To perform the *in vitro* absorption and emission studies, 10 μL of **DCIP-R** probes (10 μM) from 1 mM stock solution was added to the 1 mL of HEPES buffer solution (100 mM, pH = 8.0).

1.2.4. Concentration-dependent Kinetics

10 μM of the probes (**DCIP-R1** to **R6**) in HEPES buffer (100 mM, pH = 8.0) were used for the kinetic studies. Initially, the probes were excited at 480 nm keeping both the excitation and emission slit widths at 5 nm and the emission kinetics was monitored at 657 nm at 37 °C for

five minutes; this was followed by the addition of the respective concentration of the enzyme (PLE or hCEs2) and the kinetics was allowed to run for 30 minutes.

1.2.5. Enzyme Kinetics Assays

The Michaelis constant, K_m, was obtained from measurements carried out using a specific enzyme concentration (0.008 U/mL of PLE) and varying substrate (**DCIP-R1**) concentrations between 2 and 50 μM in HEPES buffer (100 mM, pH = 8.0). The systems were excited at 480 nm and the emission kinetics were monitored at 37 °C for 30 minutes. The specific amount of the enzymes was chosen to allow an accurate measurement of the initial rate.

Under the above conditions, the substrate concentration, [S], is significantly higher than the enzyme concentration. As long as the substrate consumption is below 20%, the initial enzymatic velocity (v) can be approximated by the Henri-Michaelis-Menten equation:

$$v = (v_{max} \text{ X } [S]) / ([S] + K_m)$$

where v_{max} is the maximal velocity at saturating substrate concentrations.

The above equation can be used to obtain the Michaelis Menten curve by plotting the measured initial enzyme velocities against the corresponding substrate concentration. The values for K_m and v_{max} were obtained by a nonlinear regression fit using OriginPro 2018. The initial velocity was calculated from the slope of the linear portion of each progress curve.

The enzyme catalytic turnover number (k_{cat}) was also obtained from the Michaelis Menten plot. The k_{cat} value is defined as:

$$k_{cat} = v_{max} \text{ X } [E_o]$$

where $[E_o]$ refers to the concentration of enzyme used. The overall catalytic efficiency of the enzymatic reaction was calculated as:

$$\text{Overall catalytic efficiency} = k_{cat} / K_m$$

1.2.6. LOD Calculation

The limit of detection (LOD) was calculated according to the following formula:

$$\text{LOD} = 3\sigma / \text{k}$$

where σ is the standard deviation of the fluorescence intensity measurements for the blank sample and k refers to the slope of the linear curve between fluorescence enhancement versus the concentration of the enzymes (PLE and hCEs2, respectively).

1.2.7. HPLC Studies

High-performance liquid chromatography (HPLC) experiments were performed in the Waters Alliance System with Waters e2695 Separation Module equipped with Waters 2998 PDA Detector. The elution system was water/ acetonitrile in a 15:85 ratio as an isocratic mode with a flow rate of 1 mL/min. The observed peaks were extracted at 390 nm wavelength.

1.2.8. Selectivity Assay

For the selectivity experiments, 10 mM stock solutions NaCl, KCl, $FeCl_3$, glucose, urea, glycine, lysine, serine, aspartic acid, monosodium glutamate, isoleucine, and glutathione were prepared in Milli-Q water. To perform the spectroscopic studies, 100 μM of these analytes were used in 1 mL of HEPES buffer (100 mM, pH = 8.0) with 10 μM of the probe (**DCIP-R1**) and 0.01 U/mL of PLE. Next, 10 mg/mL stock solutions of trypsin, chymotrypsin, HSA, DNase, RNase, and catalase were prepared in Milli-Q water. For spectroscopic studies, the working concentration of the enzymes and HSA was taken to be 10 μg/mL along with 10 μM of the probe (**DCIP-R1**) and 0.01 U/mL of PLE in 1 mL of HEPES buffer.

1.2.9. Inhibition Experiments

10 mM stock solution of 4-(2-aminoethyl) benzene sulfonyl fluoride (AEBSF) was prepared in Milli-Q water. For the spectroscopic studies, 0.5 mM, 1 mM, 1.5 mM, and 2 mM concentrations of AEBSF were used in 1 mL of HEPES buffer (100 mM, pH = 8.0) with 10 μM of the probe (**DCIP-R1**) and 0.01 U/mL of PLE.

2. Live-cell Imaging and *in vivo* Studies

Dulbecco's Modified Eagle Medium (DMEM) and the antibiotic cocktail of penicillin and streptomycin were obtained from HiMedia (USA), whereas Fetal Bovine Serum (FBS) was purchased from Sigma-Aldrich (Brazil). Cell culture imaging dishes and R1610 cells were purchased from Abidi (Germany) and National Centre for Cell Science (NCCS, Pune), respectively.

2.1. Cell Imaging

2.1.1. Cell Culture Method

R1610 cells were cultured in a 25 cm^2 cell culture flask using DMEM media supplemented with 10 % (v/v) FBS and 1 % (v/v) antibiotic cocktail of penicillin and streptomycin inside a humidity-controlled CO_2 incubator at 37 °C up to 80-90% confluency before subculture.

The Hep-G2 tumor cell line (kindly provided by Dr. Qiang Chen, University of Macau, Macau China) was maintained in Dulbecco's Modified Eagle medium (DMEM) (Sigma Aldrich, US) supplemented with 10 % heat-inactivated fetal calf serum (FCS) (Gibco, US) and 2 mM L-glutamine (Sigma Aldrich, US).

2.1.2. Cytotoxicity (CCK-8) Assay

Cytotoxicity assays were carried out using a cell counting kit-8 (CCK-8) assay. Cells were seeded in a 96-well plate at a density of 1×10^4 cells/mL in 200 μL of DMEM complete medium containing 10% FBS and 1% PS. After 24 hours of cell attachment, the cells were incubated with different concentrations (1, 5, 10, 20, 50, 100, 200, or 500 μM) of the **DCIP-R1** dye for 24 hours. Subsequently, the **DCIP-R1** dye was removed, and the cells were washed with PBS three times. 10 μL of CCK-8 dye and 100 μL of DMEM complete medium were added to each well and incubated for 4 hours at 37 °C. The absorbance of the formazan dye was measured using a microplate reader (Victor X3, Perkin-Elmer) at 450 nm, with the reference wavelength at 620 nm. The absorbance values were proportional to the number of live cells. The percent reduction in CCK-8 dye absorbance was compared to mock controls representing 100% reduction. The cell viability rate was calculated as $(A\text{-}A_0)/(A_c - A_0) \times 100\%$, where A is the absorbance of the experimental group, Ac is the absorbance of the control group (HepG-2 cells only), and A0 is the absorbance of the DMEM group (no cells). Results are presented as mean ± standard deviation (SD). The half maximal inhibitory concentration 50% (IC_{50}) values of the DCIP dye were calculated using GraphPad Prism software.

2.1.3. Fluorescence imaging of the endogenous esterase in normal cells and liver cancer cells

R1610 cells were cultured in confocal dishes until they grew to 70-80% confluency. The **DCIP-R** probes were dissolved in DMSO to reach a concentration of 1 mM. The cells were then incubated with the **DCIP-R** probes at a final concentration of 1 μM for 20 minutes at 37 °C

and washed with PBS twice before imaging. Olympus FV 3000 Confocal Laser Scanning Microscope (CLSM) was used to acquire cell images of R1610 cells, and the objective 60x oil immersion was used to visualize cells. The confocal images were processed using Olympus Cell Sens software.

HepG2 cells were cultured in confocal dishes until they grew to 70-80% confluency. The **DCIP-R** probes were dissolved in DMSO to reach a concentration of 1 mM. The cells were then incubated with the **DCIP-R** probes at a final concentration of 1 μM for 20 minutes at 37 °C and washed with PBS twice before imaging. For continuous imaging from 0 to 30 minutes, HepG2 cells were imaged immediately post the addition of **DCIP** probes, without subsequent PBS washing. Cell images were acquired using a Nikon inverted multiphoton microscope (A1MP + Eclipse Ti-2E, Nikon Instrument Inc., Japan) with a water-immersed 40x and 1.15 NA objective.

2.2 Animal Imaging

All animal experiments were conducted using protocols (UMARE0312021) approved by the Animal Ethics Committees, University of Macau. Six- to eight-week-old nude mice and six-week-old Wistar rats were bred in the Animal Facility at the Faculty of Health Sciences.

2.2.1. Imaging of DCIP-R probes in nude mice

150 μL of 1 mM **DCIR** probes dissolved in DMSO were injected into nude mice (BALB/c-nu, 6-8 weeks) *via* intraperitoneal injection. Fluorescence imaging was acquired at various time points using the AniView animal imaging system (BLT Photon Technology, China) with an excitation laser at 465 nm and an emission filter of 650-680 nm.

2.2.2. Establishment of a rat model of diethylnitrosamine (DEN)-induced hepatocellular carcinoma.

The rat DEN-induced hepatocellular carcinoma model was established as described previously.[1] Briefly, six-week-old Wistar rats with 200 g weights were administered a daily aqueous solution of 50 ppm (v/v) DEN (Sigma-Aldrich, USA) as drinking water for 42 days, while one control rat received normal water. Following the DEN feeding period, all rats were given normal water until they reached 19 weeks of age, at which point they were sacrificed for liver tissue/cancer collection. Fresh liver tissue/cancer samples were sectioned into 100 μm slices for the imaging of **DCIP-R** probes. 150 μL of 1 mM **DCIP-R** probes dissolved in DMSO

were added to rat liver tissue/ tumor slices. Fluorescence imaging was acquired at various time points using the AniView animal imaging system (BLT Photon Technology, China) with an excitation laser at 465 nm and an emission filter of 650-680 nm.

2.2.3. Establishment of HepG-2 liver tumor model.

1 x 10^6 HepG-2 tumor cells were subcutaneously injected into the left flank of nude mice. When tumors reached about 1,000 mm^3, tumors were isolated and sectioned. Subsequently, 150 μL of 1 mM **DCIP-R** probes were added to mouse liver tumor slices. Fluorescence imaging was performed at various time points using the AniView animal imaging system with an excitation laser at 465 nm and an emission filter of 650-680 nm.

3. Design and synthesis of DCIP-OH and DCIP-R probes

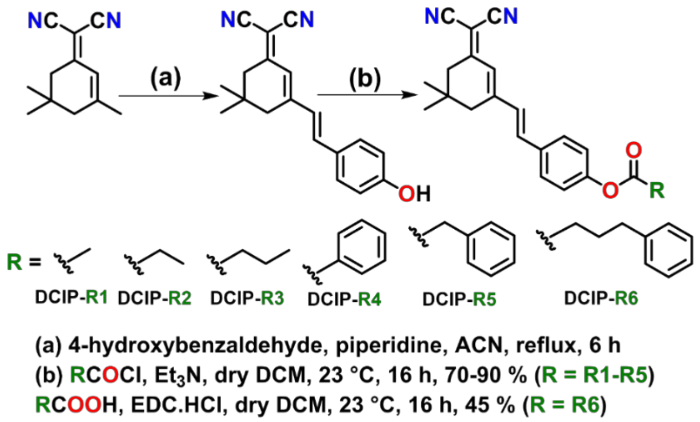

Scheme S1: Synthesis of the dicyanoisophorone-based (**DCIP-R**) probes.

3.1. Synthesis of compound DCIP-OH:[2] Compound **1** (1 g, 5.4 mmol, 1 equiv.), 4-hydroxybenzaldehyde (886 mg, 5.9 mmol, 1.1 equiv.), and piperidine (0.5 mL) were dissolved in acetonitrile and refluxed the reaction mixture at 82 ºC for 6 h. After the completion of the reaction, the solvent was removed under reduced pressure to obtain a crude reaction mixture. The obtained residue was extracted using DCM, and the organic layer was dried over anhydrous Na_2SO_4 and concentrated to dryness to get the crude product. The required product **DCIP-OH** was obtained as an orange solid by purifying the reaction mixture using silica gel column chromatography with DCM: methanol (50:1) as eluent (675 mg, 45 %). ^{1}H NMR (500 MHz, $CDCl_3$) δ 7.42 (d, J = 8.6 Hz, 1H), 7.01 (d, J = 16.0 Hz, 1H), 6.87-6.83 (m, 3H), 6.80 (s, 1H), 2.59 (s, 2H), 2.45 (s, 2H), 1.07 (s, 6H). ^{13}C[^{1}H] NMR (500 MHz, $CDCl_3$) δ 169.48, 157.33, 154.44, 136.91, 129.54, 128.83, 127.24, 122.94, 116.23, 113.82, 113.06, 78.01, 43.07, 32.29,

32.19, 28.18. HRMS (ESI), Calculated mass = 290.1419 Da, Obtained mass = 313.1311 Da [M+Na$^+$].

Synthesis of compound DCIP-R1-R5: Compound **DCIP-OH** (30 mg, 0.1 mmol, 1 equiv.) was dissolved in dry DCM, added a few drops of triethylamine, and stirred for 15 minutes at 0 ºC. To the resulting reaction mixture, corresponding acid chloride (10.64 μL, 0.15 mmol, 1.5 equiv.) was added dropwise and stirred at 23 ºC till TLC revealed the complete conversion of starting material into a product (~16 h). The solvent was removed under reduced pressure and the crude reaction mixture was washed using distilled hexane. The required product **DCIP-R1-R5** was obtained as yellow solid by purifying the reaction mixture using silica gel column chromatography with DCM: hexane (1:1) as eluent.

3.2. DCIP-R1: Yield = (29 mg, 85.2 %.).^{1}H NMR (700 MHz, $CDCl_3$) δ 7.52 (d, J = 8.6 Hz, 2H), 7.13 (d, J = 8.6 Hz, 2H), 7.03 (d, J = 16.1 Hz, 1H), 6.94 (d, J = 16.1 Hz, 1H), 6.84 (s, 1H), 2.60 (s, 2H), 2.46 (s, 2H), 2.32 (s, 3H), 1.08 (s, 6H). ^{13}C[^{1}H] NMR (500 MHz, $CDCl_3$) δ 169.33, 153.69, 151.77, 135.96, 133.52, 129.44, 128.72, 123.86, 122.41, 113.56, 112.79, 79.07, 43.13, 39.35, 32.17, 28.16, 21.28. HRMS (ESI), Calculated mass = 332.1525 Da, Obtained mass = 355.1417 Da [M+Na$^+$].

3.3. DCIP-R2: (25 mg, 70 %).^{1}H NMR (500 MHz, $CDCl_3$) δ 7.52 (d, J = 8.5 Hz, 2H), 7.13 (d, J = 8.5 Hz, 2H), 7.03 (d, J = 16.0 Hz, 1H), 6.94 (d, J = 16.3 Hz, 1H), 6.84 (s, 1H), 2.61 (p, J = 7.6 Hz, 4H), 2.47 (s, 2H), 1.28 (t, J = 7.5 Hz, 3H), 1.08 (s, 6H)). ^{13}C[^{1}H] NMR (500 MHz, $CDCl_3$) δ 172.86, 169.35, 153.73, 151.93, 136.04, 133.41, 129.38, 128.72, 123.85, 122.42, 113.59, 112.81, 79.07, 43.16, 39.37, 32.19, 28.18, 27.92, 9.17. HRMS (ESI), Calculated mass = 346.1681 Da, Obtained mass = 369.1573 Da [M+Na$^+$].

3.4. DCIP-R3: (22 mg, 59.3 %).^{1}H NMR (500 MHz, $CDCl_3$) δ 7.52 (d, J = 8.6 Hz, 2H), 7.13 (d, J = 8.6 Hz, 2H), 7.03 (d, J = 16.1 Hz, 1H), 6.94 (d, J = 16.1 Hz, 1H), 6.84 (s, 1H), 2.60 (s, 2H), 2.55 (t, J = 7.4 Hz, 2H), 2.46 (s, 2H), 1.79 (dt, J = 14.8, 7.4 Hz, 2H), 1.08 (s, 8H), 1.05 (t, J = 7.4 Hz, 3H). ^{13}C[^{1}H] NMR (500 MHz, $CDCl_3$) δ 172.02, 169.34, 153.73, 151.89, 136.03, 133.39, 129.35, 128.70, 123.82, 122.43, 113.58, 112.80, 79.02, 43.13, 39.35, 36.35, 32.17, 28.16, 18.54, 13.76. HRMS (ESI), Calculated mass = 360.1838 Da, Obtained mass = 361.1911 Da [M+H$^+$].

3.5. DCIP-R4: (25 mg, 62 %).^{1}H NMR (500 MHz, $CDCl_3$) δ 8.21 (d, J = 7.2 Hz, 2H), 7.66 (t, J = 6.9 Hz, 1H), 7.58 (d, J = 8.6 Hz, 2H), 7.53 (t, J = 7.8 Hz, 3H), 7.28 (s, 2H), 7.07 (d, J = 16.1 Hz, 1H), 6.98 (d, J = 16.1 Hz, 1H), 6.86 (s, 1H), 2.62 (s, 2H), 2.49 (s, 2H), 1.10 (s, 6H). ^{13}C[^{1}H] NMR (500 MHz, $CDCl_3$) δ 169.34, 165.06, 153.72, 152.10, 136.03, 133.98, 133.59, 130.36, 129.47, 129.35, 128.80, 123.88, 122.57, 113.58, 112.80, 79.08, 43.15, 39.37, 32.19, 28.17. HRMS (ESI), Calculated mass = 394.1681 Da, Obtained mass = 395.1754 Da [M+H^{+}].

3.6. DCIP-R5: (23.5 mg, 55.8 %).^{1}H NMR (500 MHz, $CDCl_3$) δ 7.50 (d, J = 8.6 Hz, 2H), 7.40 – 7.37 (m, 4H), 7.35 – 7.29 (m, 1H), 7.11 (d, J = 8.6 Hz, 2H), 7.02 (d, J = 16.1 Hz, 1H), 6.93 (d, J = 16.1 Hz, 1H), 6.84 (s, 1H), 3.87 (s, 2H), 2.60 (s, 2H), 2.46 (s, 2H), 1.08 (s, 6H). ^{13}C[^{1}H] NMR (500 MHz, $CDCl_3$) δ 169.14, 164.86, 153.52, 151.90, 135.83, 133.77, 133.38, 130.15, 129.27, 129.15, 128.60, 123.68, 122.37, 113.38, 112.60, 78.88, 42.94, 39.16, 31.98, 27.97. HRMS (ESI), Calculated mass = 408.1838 Da, Obtained mass = 409.1911 Da [M+H^{+}].

3.7. Synthesis of compound DCIP-R6: To the solution of 4-phenylbutanoic acid (15 mg, 0.091 mmol, 1 equiv.) in dry DCM, EDC.HCl (35 mg, 0.18 mmol, 2 equiv.) was added and stirred for 20 minutes. Compound **DCIP-OH** was added dropwise to the resulting reaction mixture and stirred at 23 ºC for till TLC revealed the completion of the reaction (~16 h). The solvent was removed under reduced pressure and the crude reaction mixture was extracted using DCM. The required product **DCIP-R6** was obtained as a yellow solid by purifying the reaction mixture using silica gel column chromatography with DCM: hexane (2:3) as eluent (7 mg, 60.0 %).^{1}H NMR (500 MHz, $CDCl_3$) δ 7.51 (d, J = 8.6 Hz, 2H), 7.34 – 7.29 (m, 2H), 7.22 (dd, J = 7.3, 4.0 Hz, 3H), 7.11 (d, J = 8.6 Hz, 2H), 7.03 (d, J = 16.2 Hz, 1H), 6.94 (d, J = 16.1 Hz, 1H), 6.84 (s, 1H), 2.75 (t, J = 7.5 Hz, 2H), 2.64 – 2.54 (m, 4H), 2.46 (s, 1H), 2.14 – 2.02 (m, 2H), 1.08 (s, 6H). ^{13}C[^{1}H] NMR (500 MHz, $CDCl_3$) δ 171.59, 169.12, 153.67, 151.85, 141.21, 136.00, 133.47, 129.42, 128.73, 126.30, 123.88, 122.41, 113.58, 112.81, 79.09, 43.16, 39.37, 35.19, 33.79, 32.19, 29.85, 28.18, 26.54. HRMS (ESI), Calculated mass = 436.2151 Da, Obtained mass = 459.2043 Da [M+Na^{+}].

4. ¹H-NMR, ¹³C-NMR, and HRMS spectra

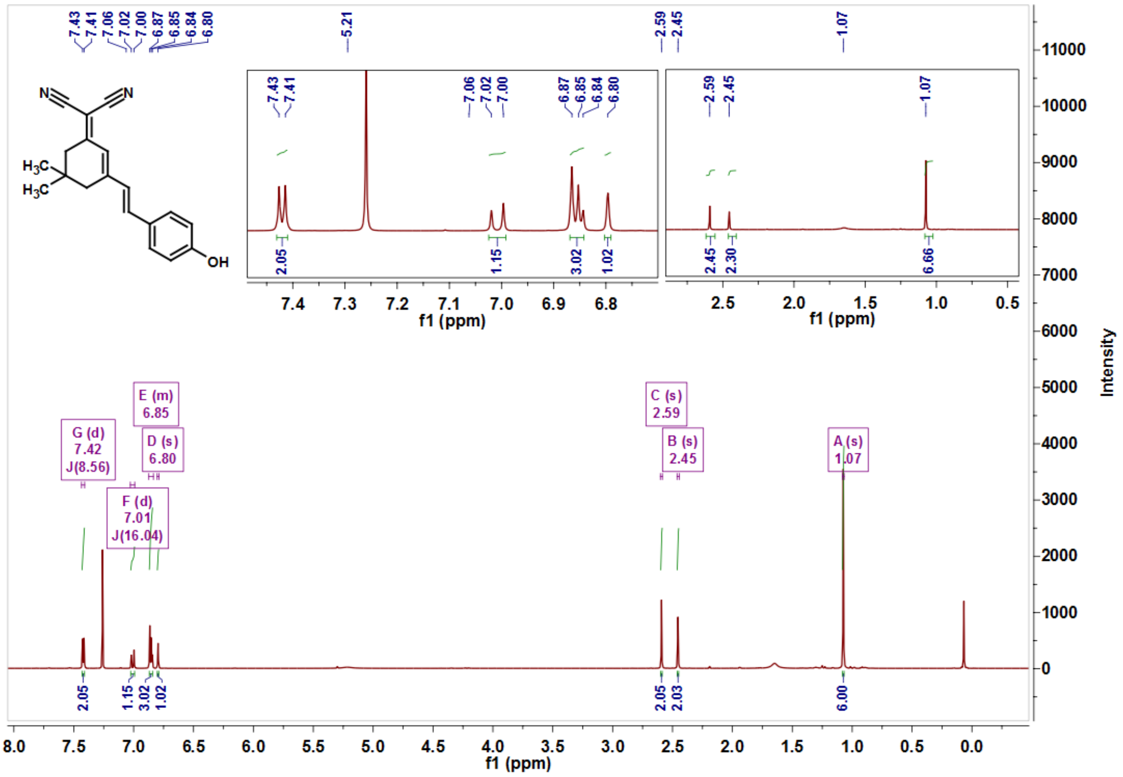

Figure S1: ¹H NMR for compound **DCIP-OH** recorded in $CDCl_3$ at 500 MHz

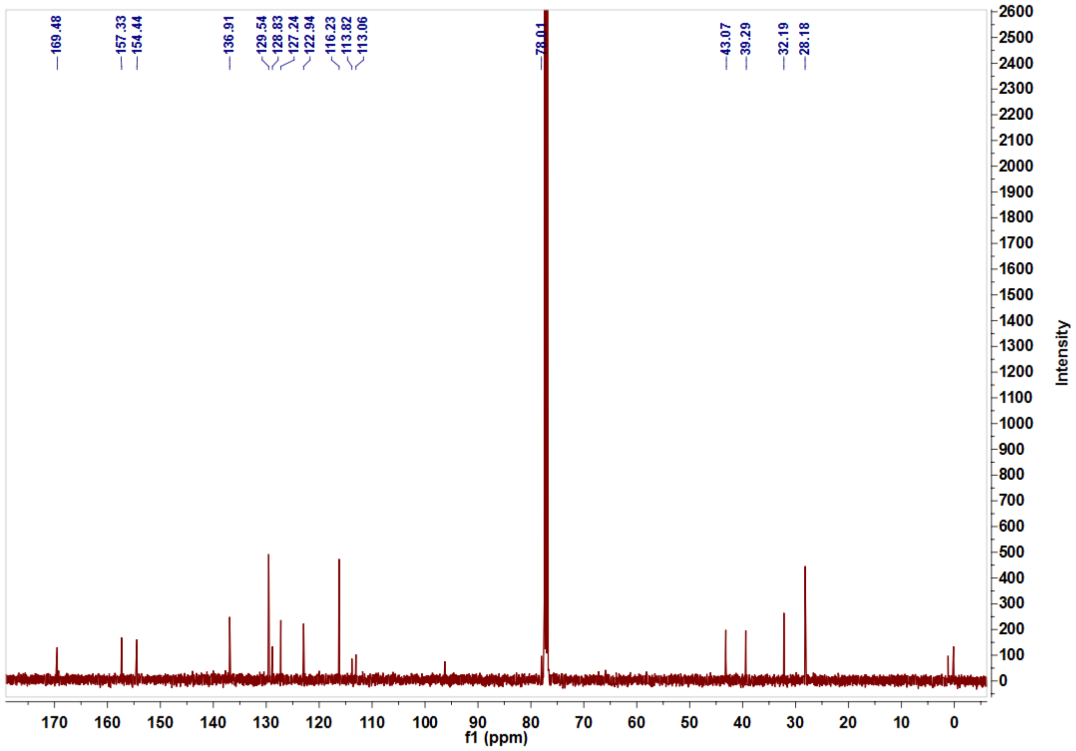

Figure S2: $^{13}C[^{1}H]$ NMR for compound **DCIP-OH** recorded in $CDCl_3$.

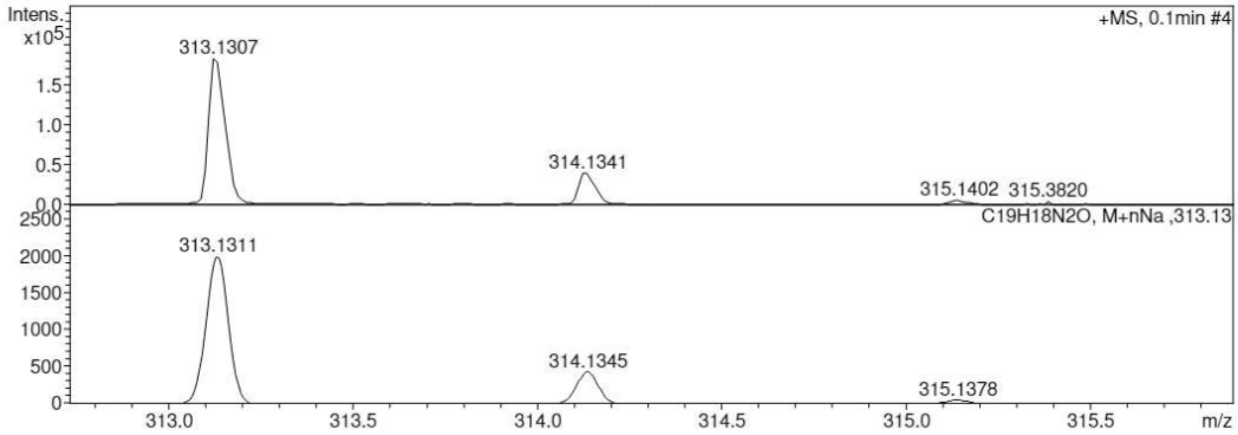

Figure S3: HRMS for compound **DCIP-OH**; **Calculated mass** = 290.1419 Da, **Obtained mass** = 313.1311 Da **[M+Na⁺]**.

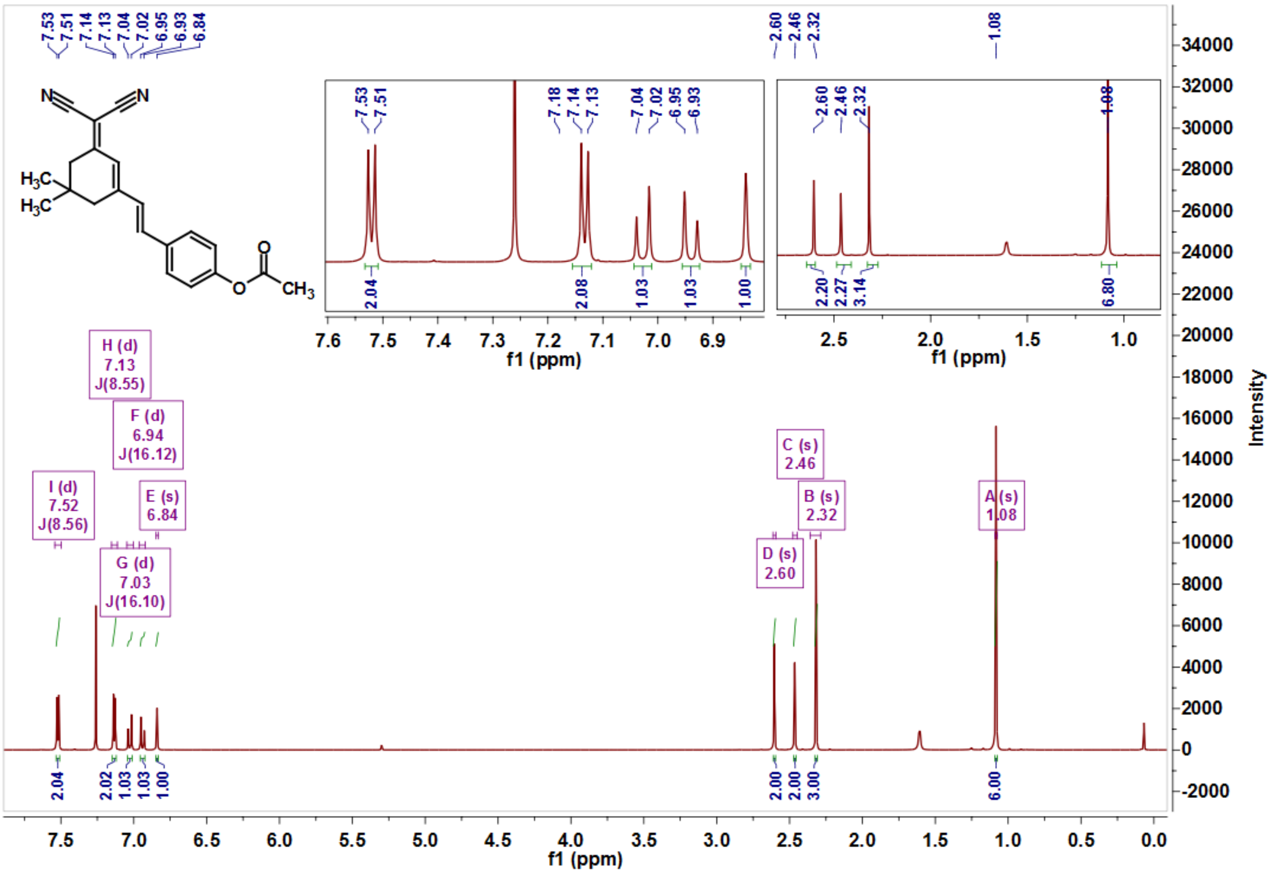

Figure S4: ^{1}H NMR for compound **DCIP-R1** recorded in $CDCl_3$ at 500 MHz.

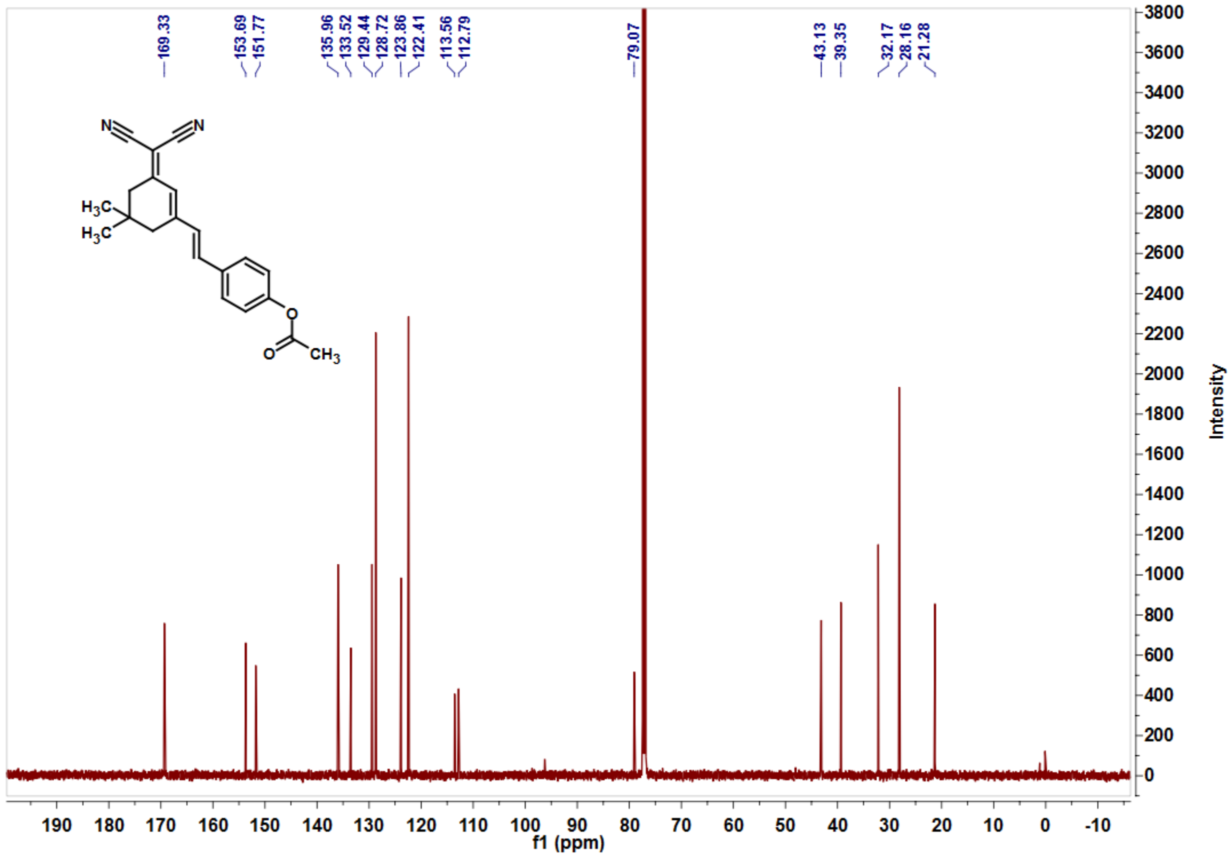

Figure S5: $^{13}C[^{1}H]$ NMR for compound **DCIP-R1** recorded in $CDCl_3$.

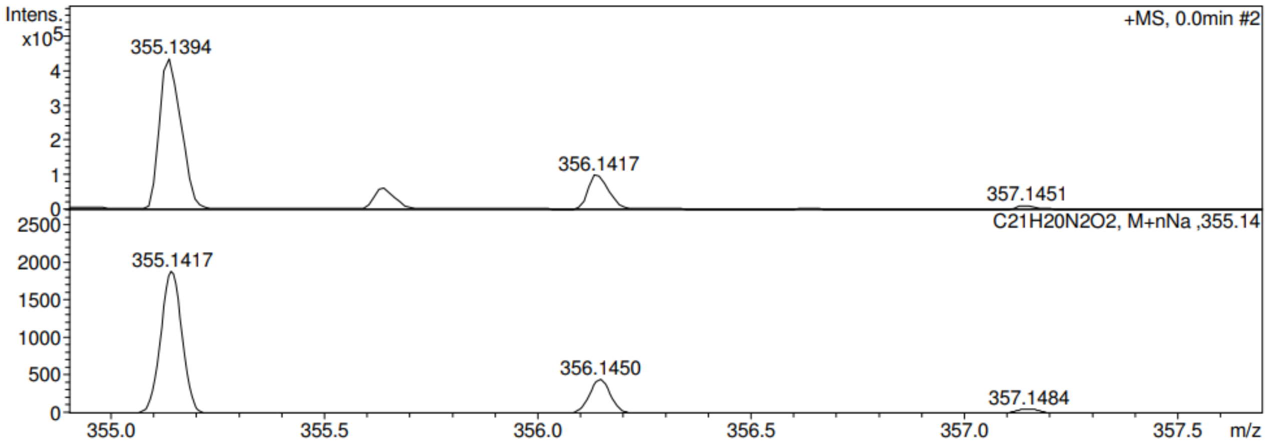

Figure S6: HRMS for compound **DCIP-R1**; **Calculated mass** = 332.1525 Da, **Obtained mass** = 355.1417 Da **[M+Na^{+}]**.

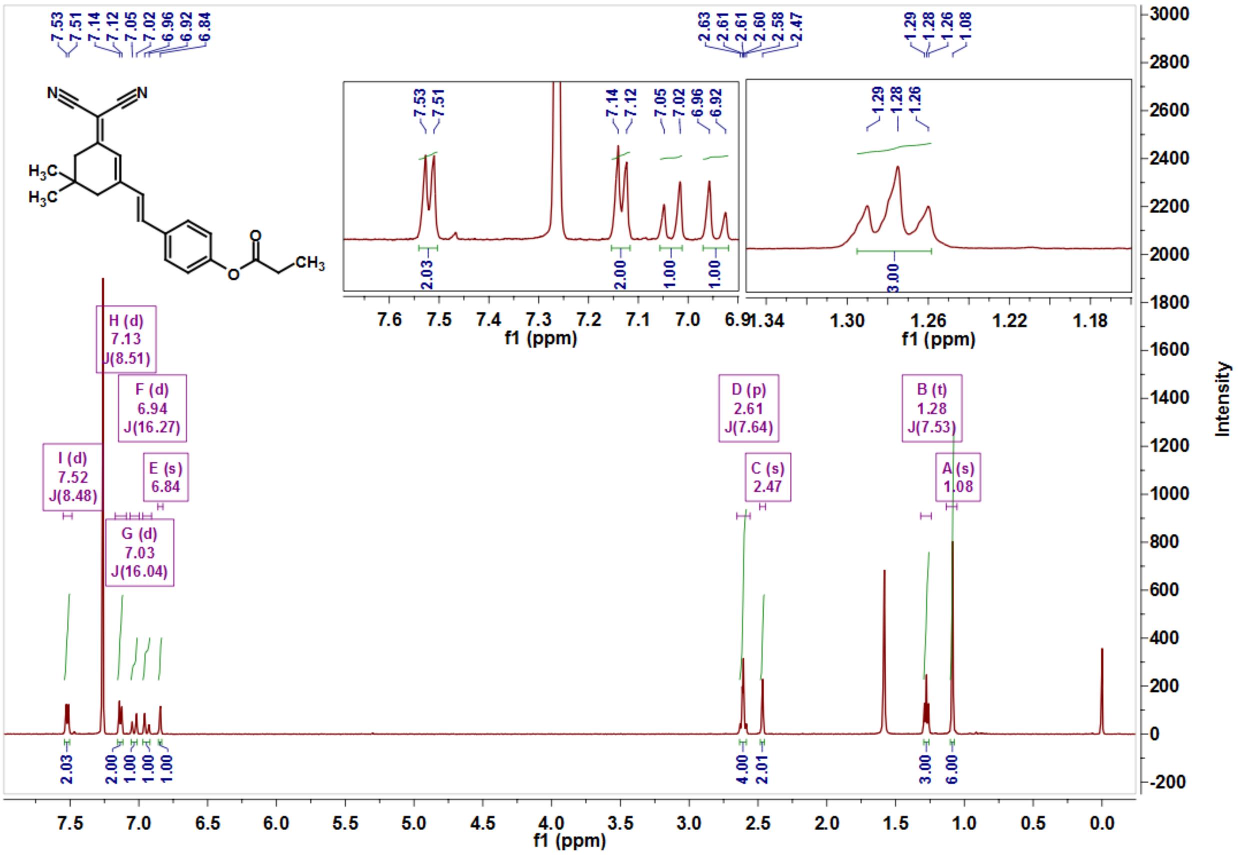

Figure S7: ^{1}H NMR for compound **DCIP-R2** recorded in $CDCl_3$ at 500 MHz.

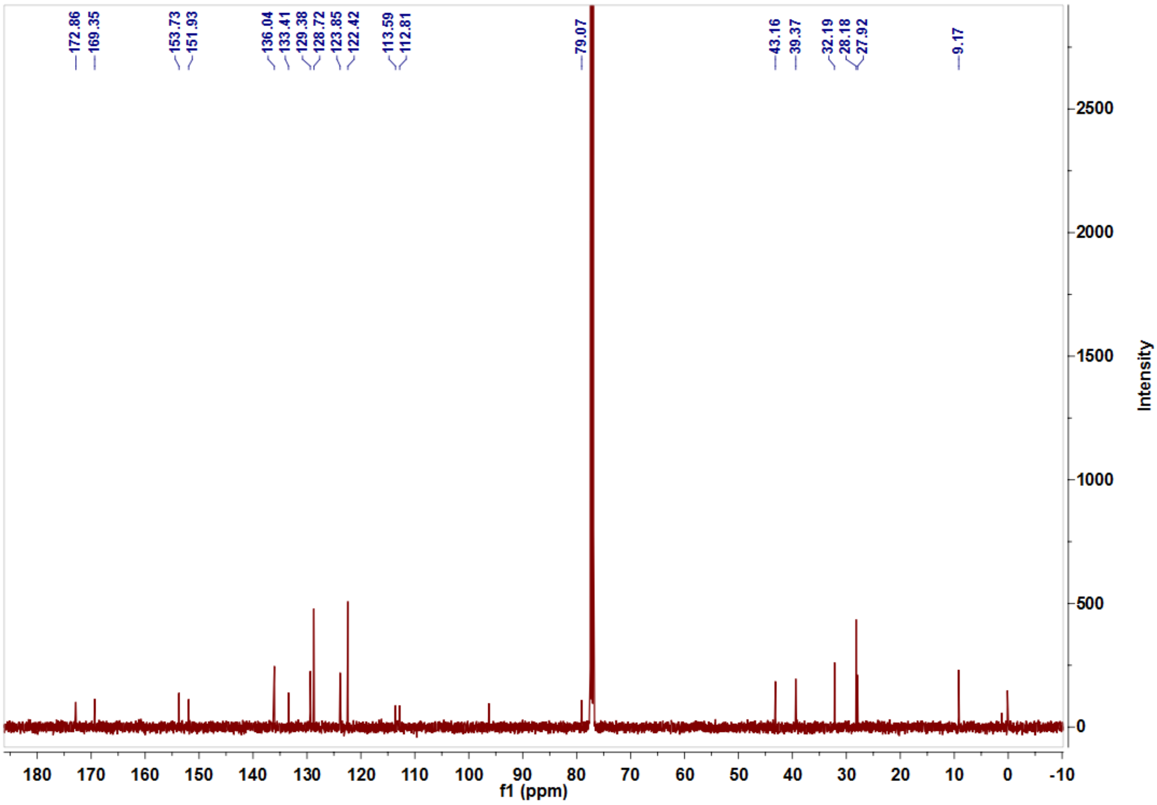

Figure S8: $^{13}C[^{1}H]$ NMR for compound **DCIP-R2** recorded in $CDCl_3$.

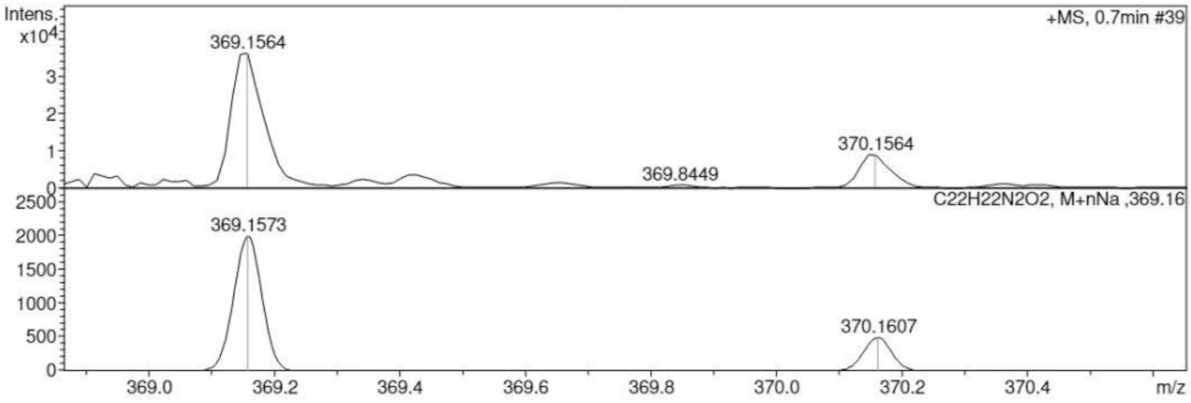

Figure S9: HRMS for compound **DCIP-R2**; **Calculated mass** = 346.1681 Da, **Obtained mass** = 369.1573 Da **[M+Na$^+$]**.

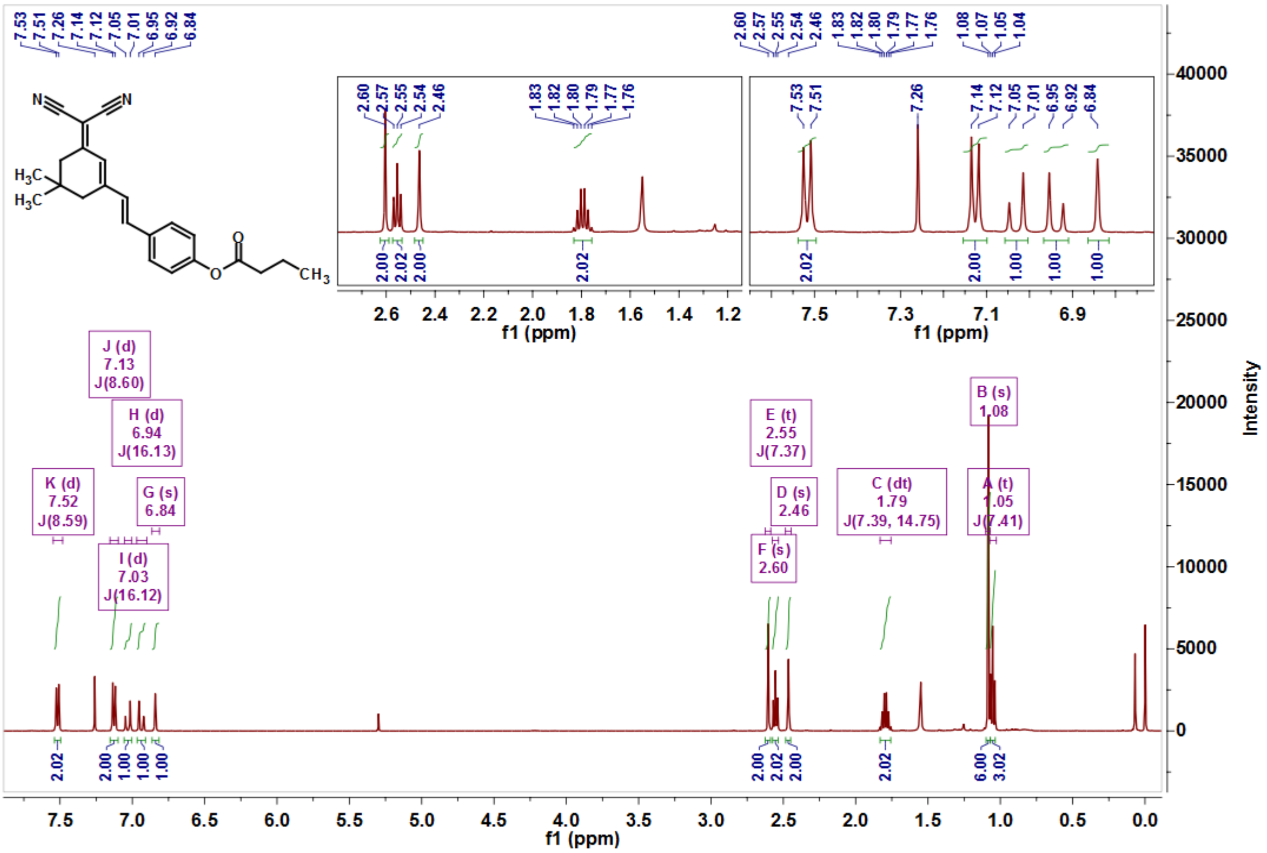

Figure S10: ^{1}H NMR for compound **DCIP-R3** recorded in $CDCl_3$ at 500 MHz.

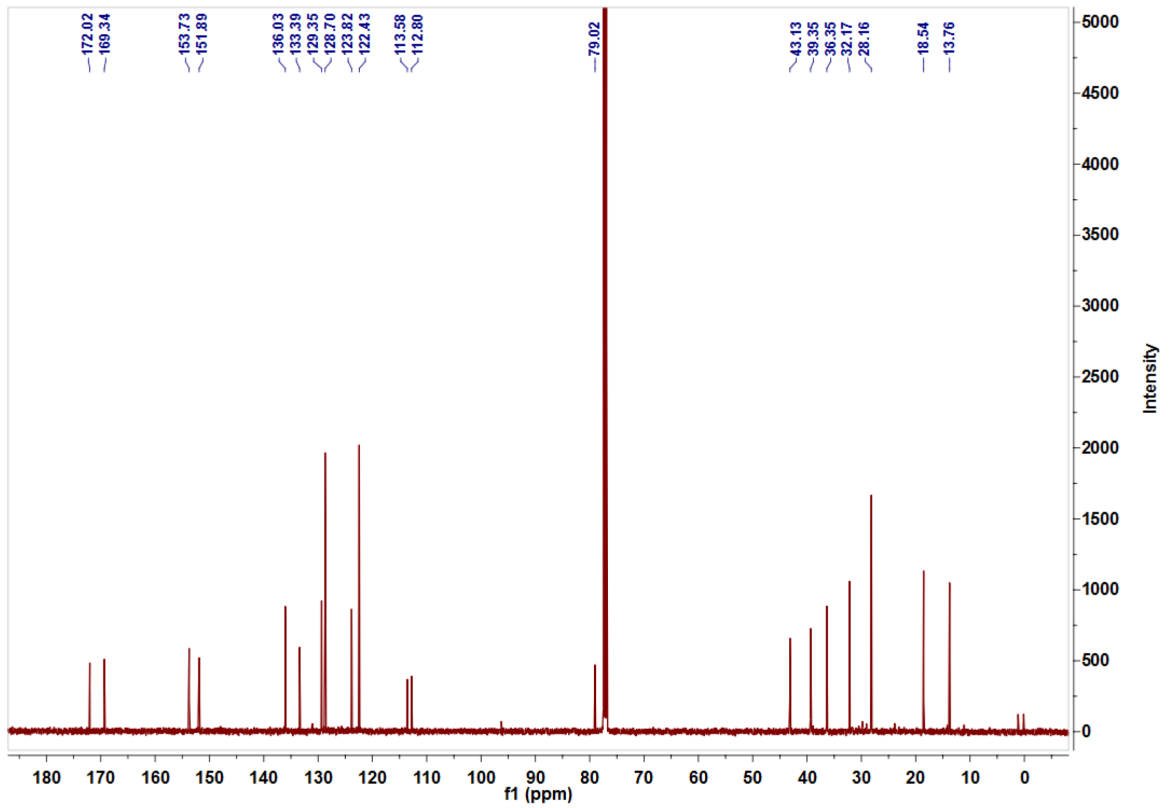

Figure S11: $^{13}C[^{1}H]$ NMR for compound **DCIP-R3** recorded in $CDCl_3$.

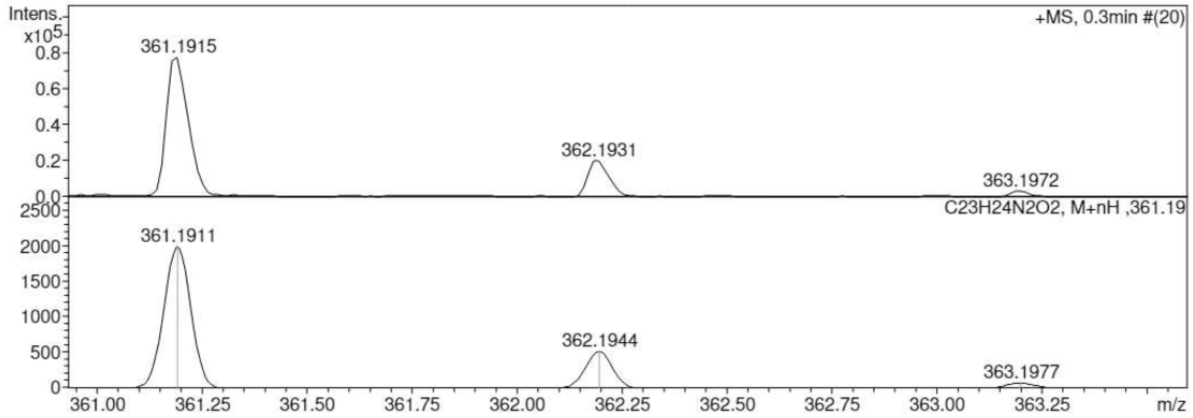

Figure S12: HRMS for compound **DCIP-R3**; **Calculated mass** = 360.1838 Da, **Obtained mass** = 361.1911 Da **[M+H$^+$]**.

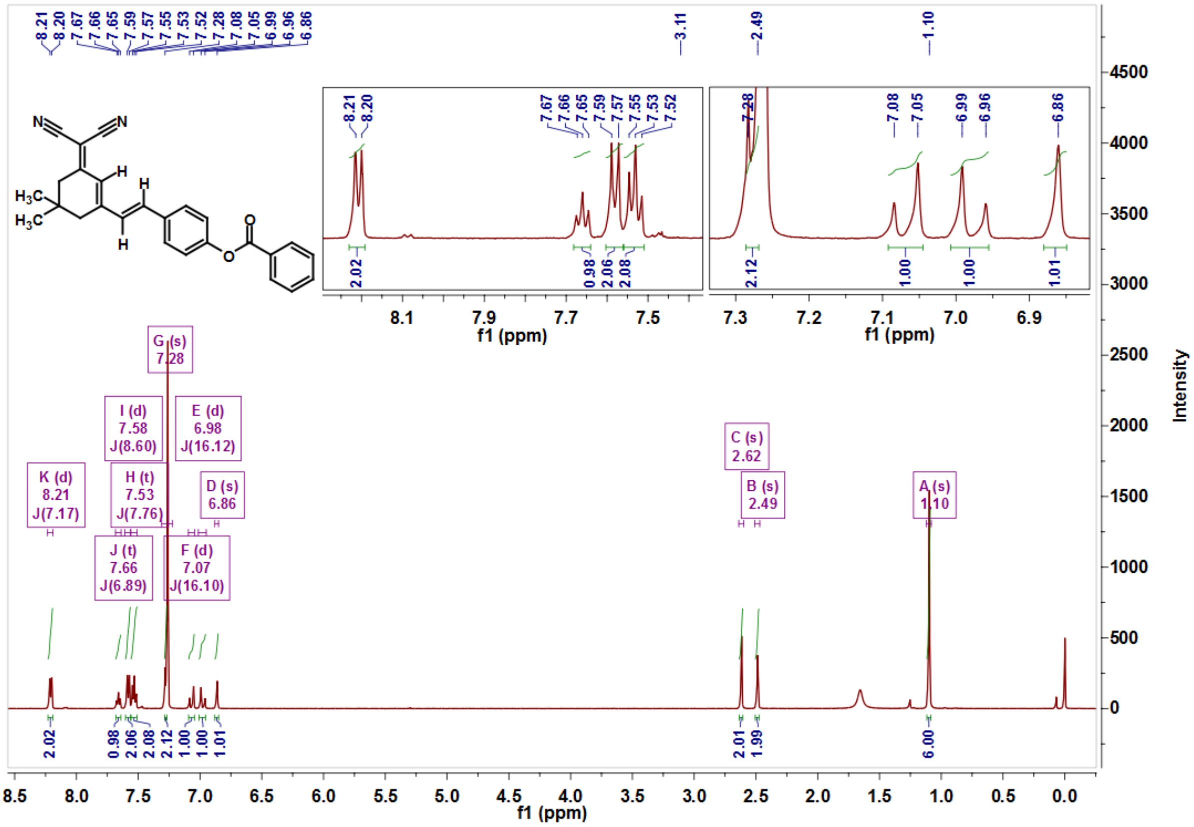

Figure S13: ^{1}H NMR for compound **DCIP-R4** recorded in $CDCl_3$ at 500 MHz.

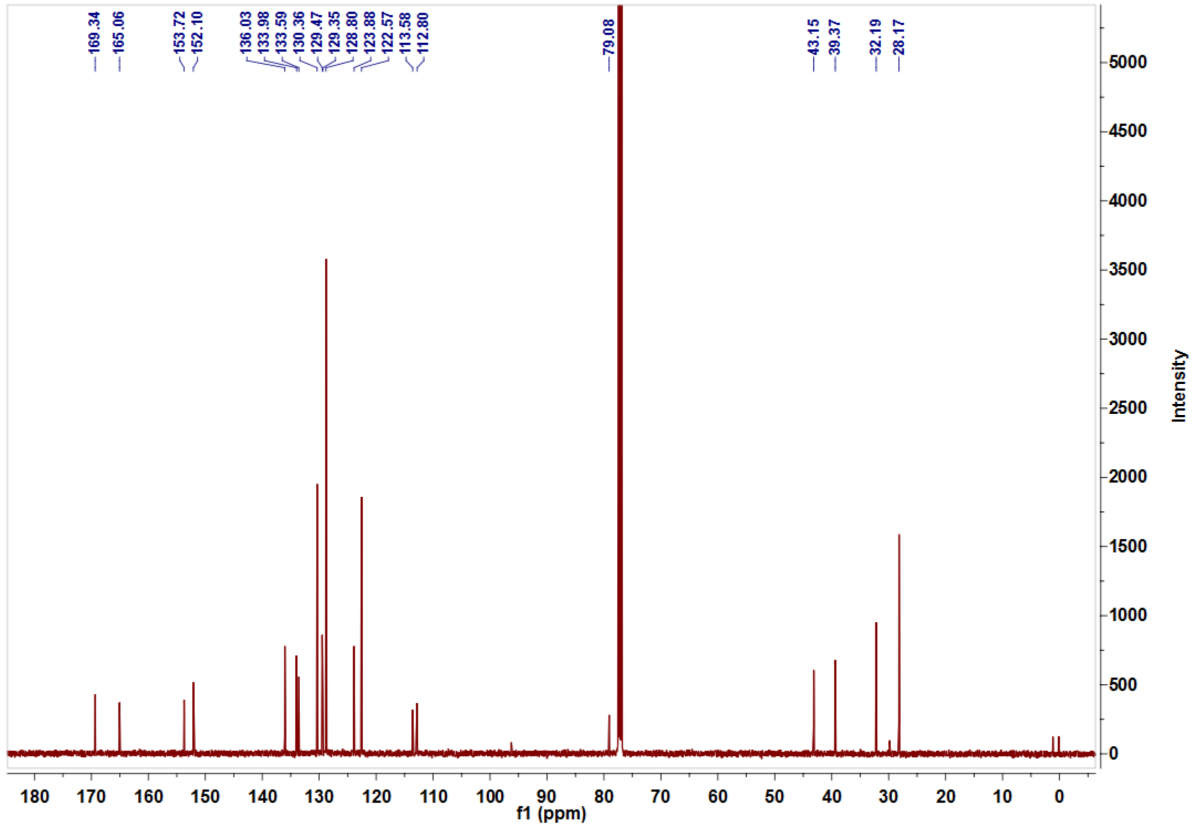

Figure S14: $^{13}C[^{1}H]$ NMR for compound **DCIP-R4** recorded in $CDCl_3$.

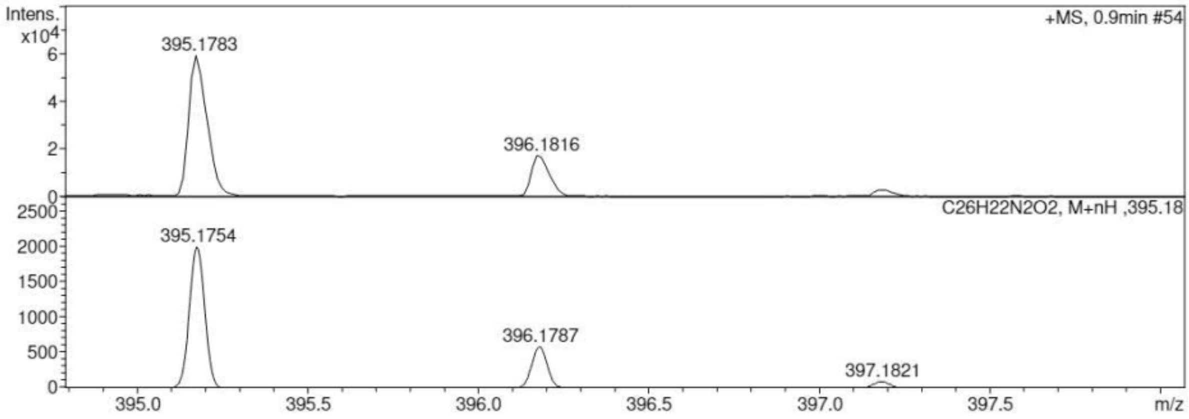

Figure S15: HRMS for compound **DCIP-R4**; **Calculated mass** = 394.1681 Da, **Obtained mass** = 395.1754 Da **[M+H$^+$]**.

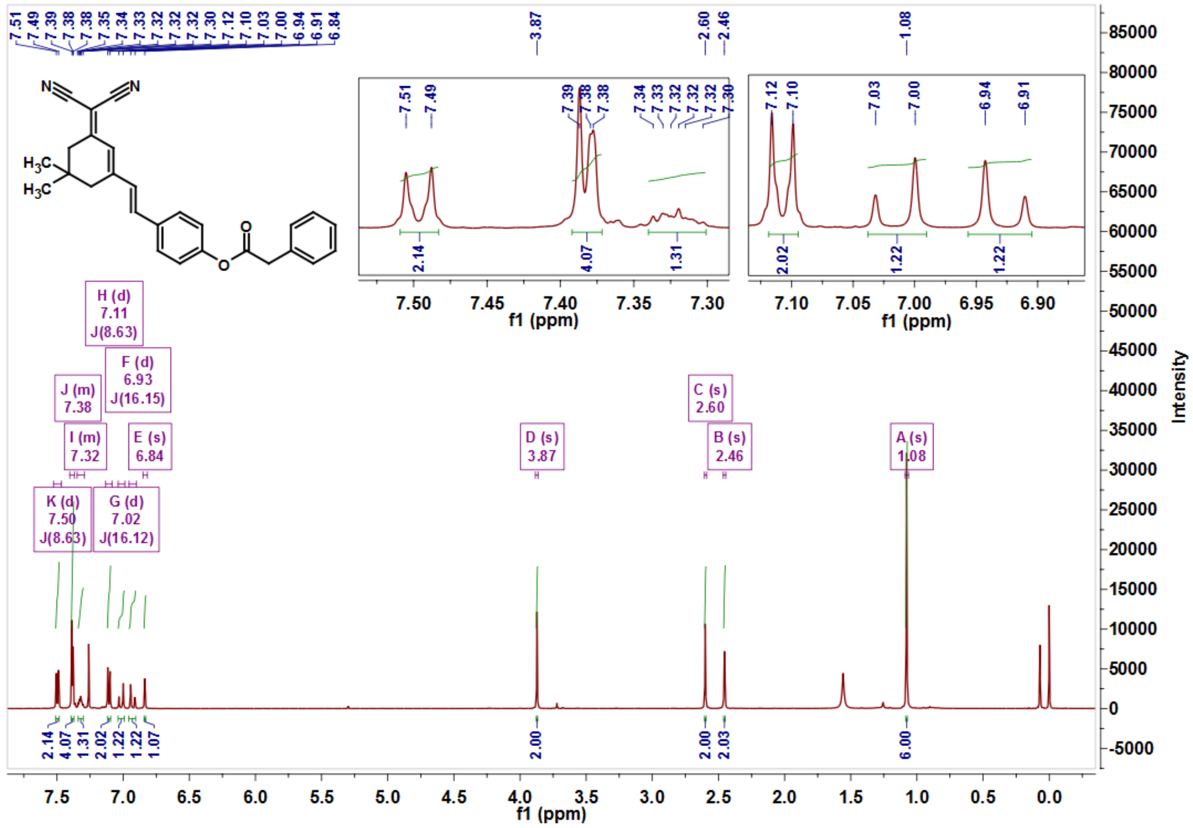

Figure S16: ^{1}H NMR for compound **DCIP-R5** recorded in $CDCl_3$ at 500 MHz.

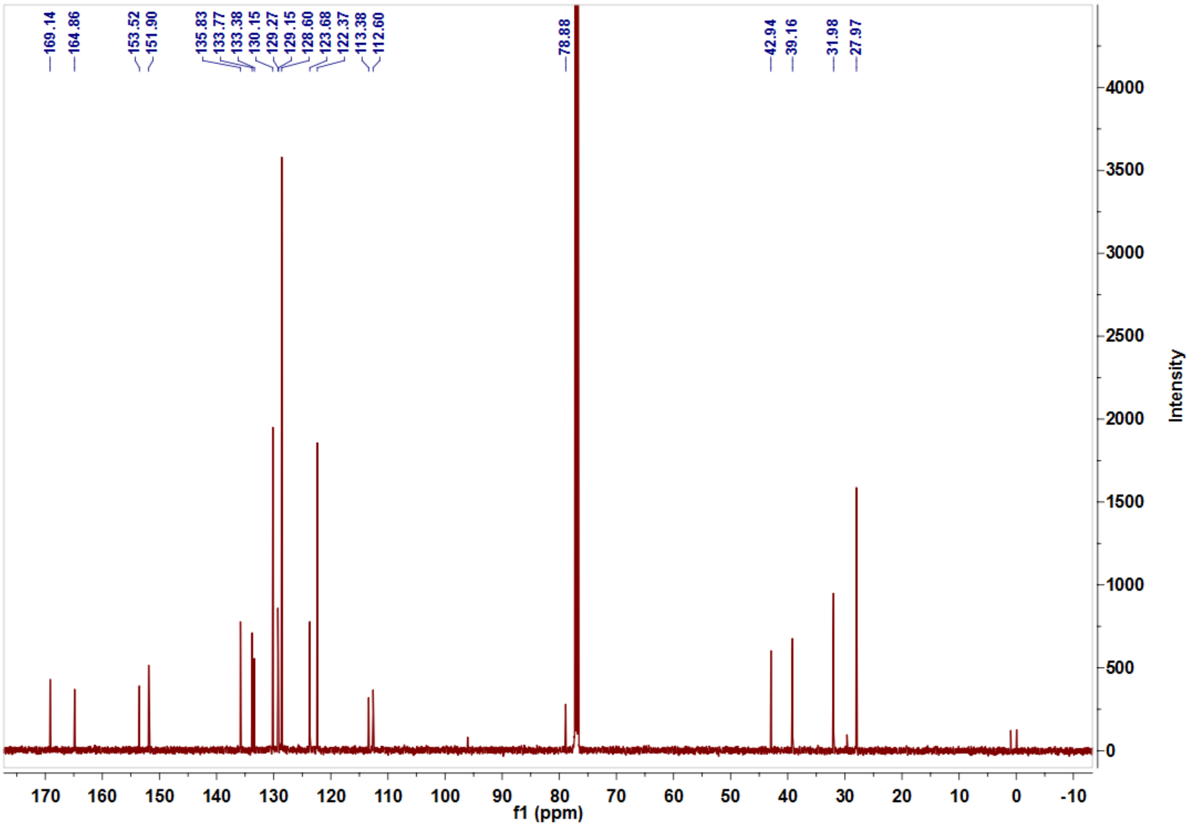

Figure S17: $^{13}C[^{1}H]$ NMR for compound **DCIP-R5** recorded in $CDCl_3$.

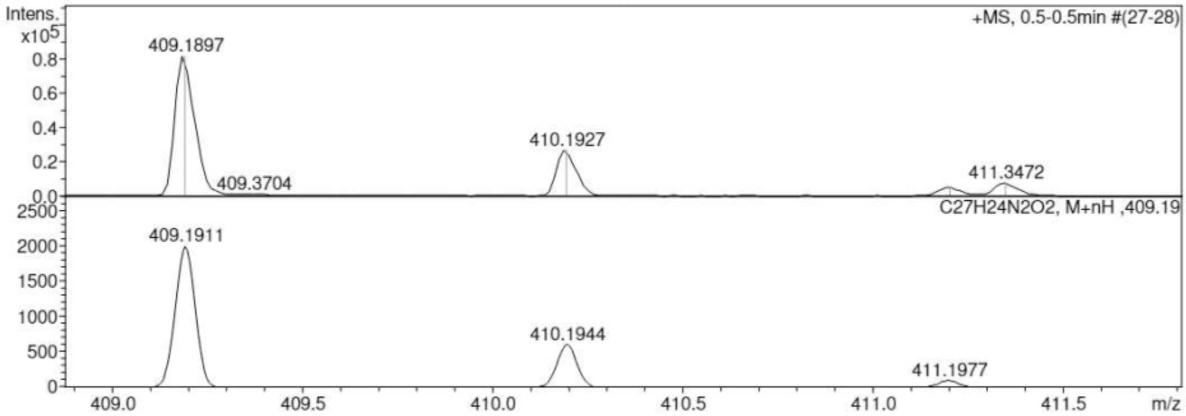

Figure S18: HRMS for compound **DCIP-R5**; **Calculated mass** = 408.1838 Da, **Obtained mass** = 409.1911 Da **[M+H$^+$]**.

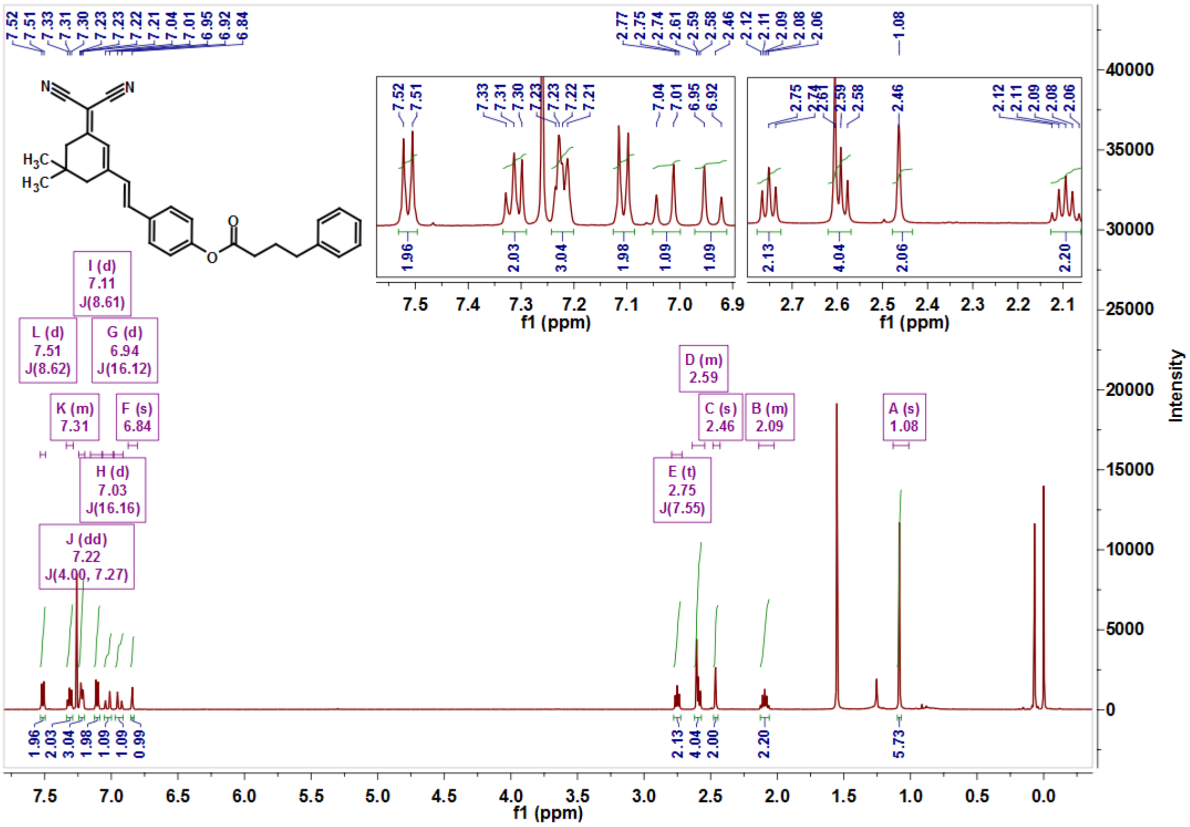

Figure S19: ^{1}H NMR for compound **DCIP-R6** recorded in $CDCl_3$ at 500 MHz.

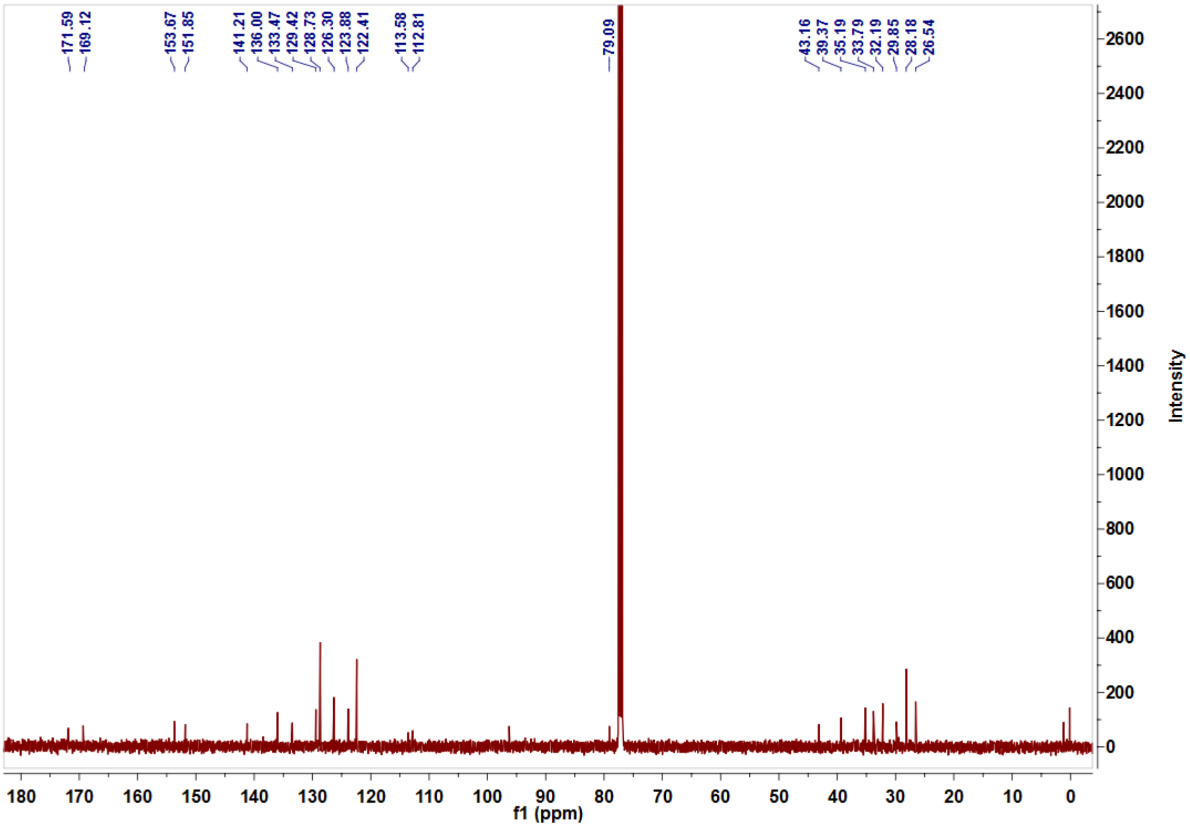

Figure S20: $^{13}C[^{1}H]$ NMR for compound **DCIP-R6** recorded in $CDCl_3$.

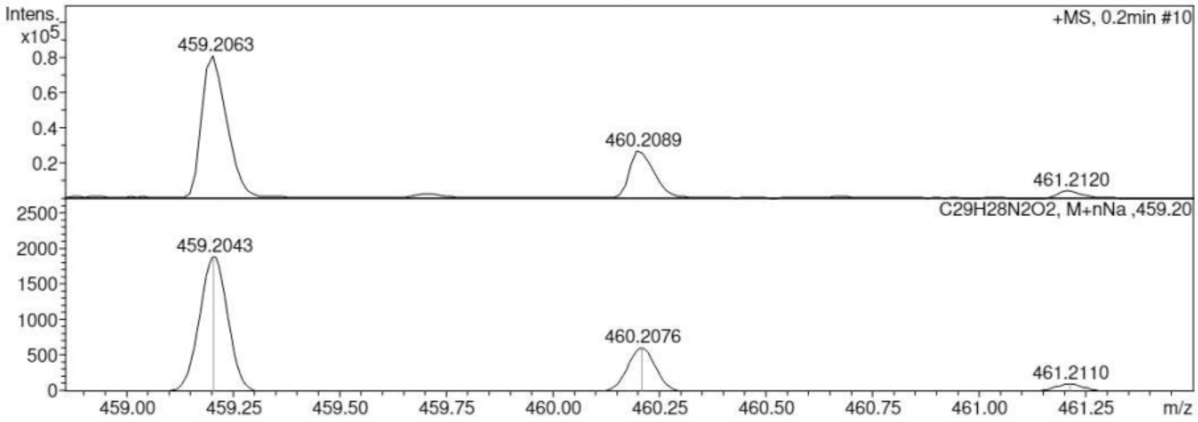

Figure S21: HRMS for compound **DCIP-R6**; **Calculated mass** = 436.2151 Da, **Obtained mass** = 459.2043 Da **[M+Na$^+$]**.

5. Photophysical properties of the probes

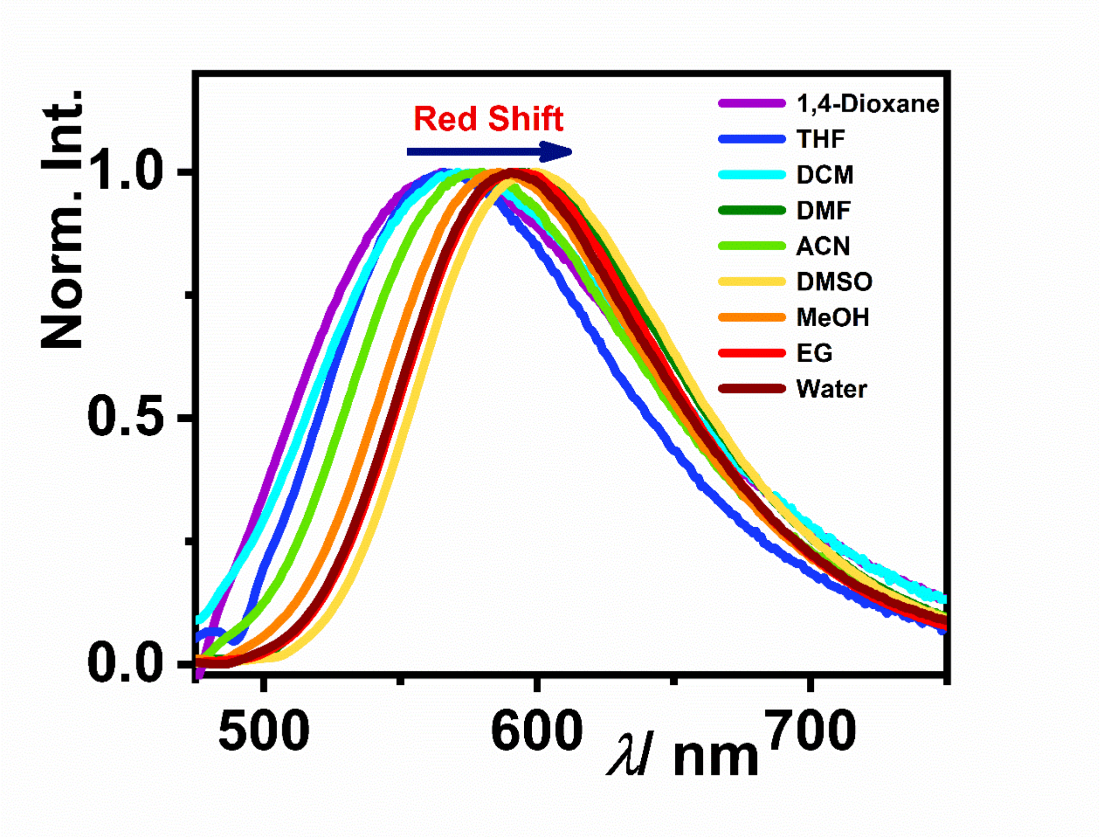

Figure S22: Normalized emission spectra of **DCIP-OH** with varying solvent polarity; [**DCIP-OH**] = 10 μM.

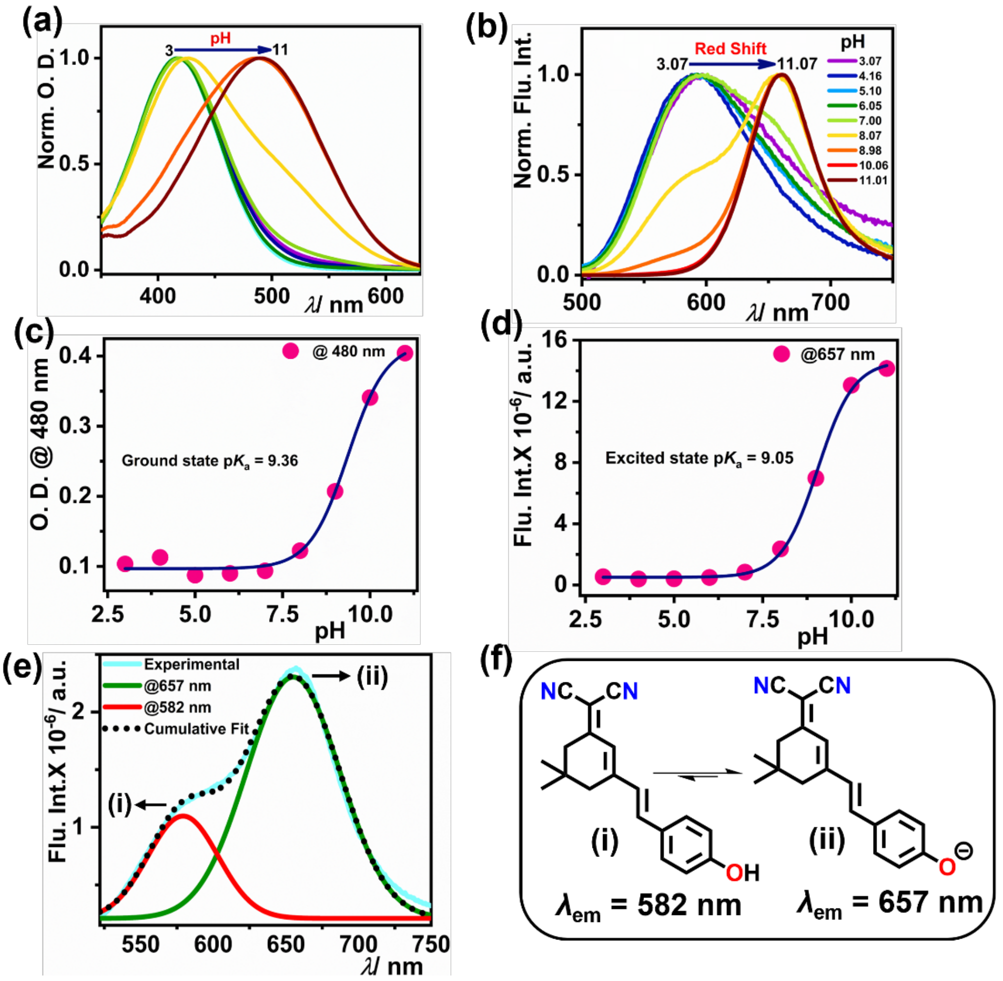

Figure S23: pH-dependent **(a)** UV-Vis. absorption and **(b)** emission spectra for **DCIP-OH,** λ_{ex} = 480 **(c)** and **(d)** are sigmoidal curves of pH-dependent absorption and emission for **DCIP-OH** (10 μM), (e) Experimental and de-convoluted spectra for **DCIP-OH** show the emission at 582 nm and 657 nm corresponding to phenol and phenoxide form respectively as given in (f).

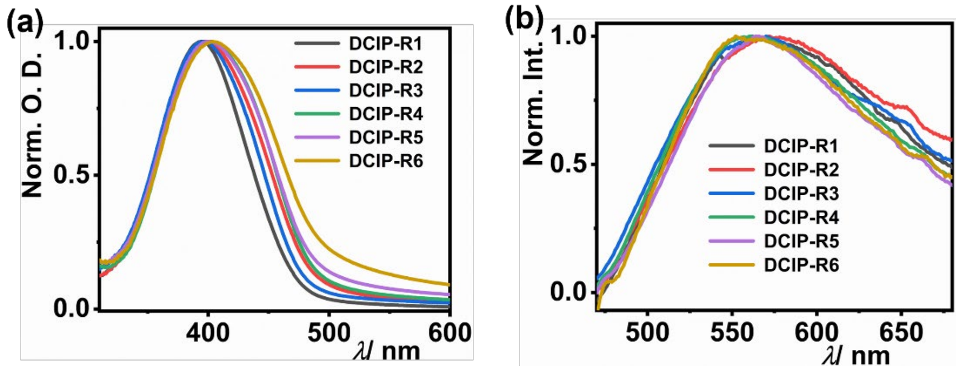

Figure S24: Normalized **(a)** UV-Vis. absorption and **(b)** emission spectra of **DCIP-R** probes in HEPES buffer (100 mM; pH = 8.0).

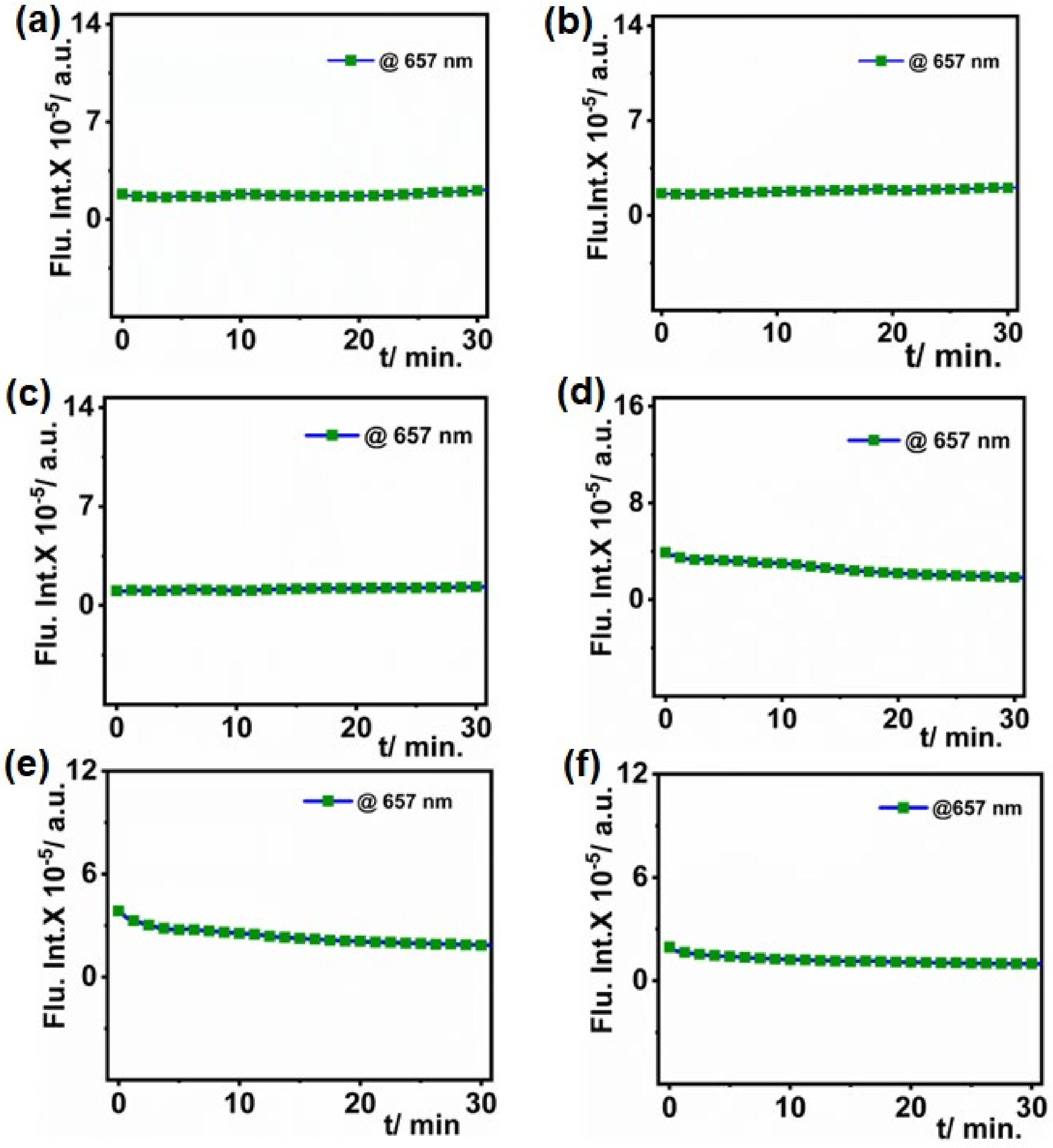

Figure S25: Photostability of DCIP-R probes **(a) DCIP-R1**, **(b) DCIP-R2**, **(c) DCIP-R3**, **(d) DCIP-R4**, **(e) DCIP-R5**, **(f) DCIP-R6**, performed in HEPES buffer (pH = 8.0, 100 mM) at 37 ºC; λ_{ex} = 480, monitored at 657 nm, [**DCIP-R**] = 10 μM.

6. UV-Vis. absorption and emission studies of DCIP-R with esterase

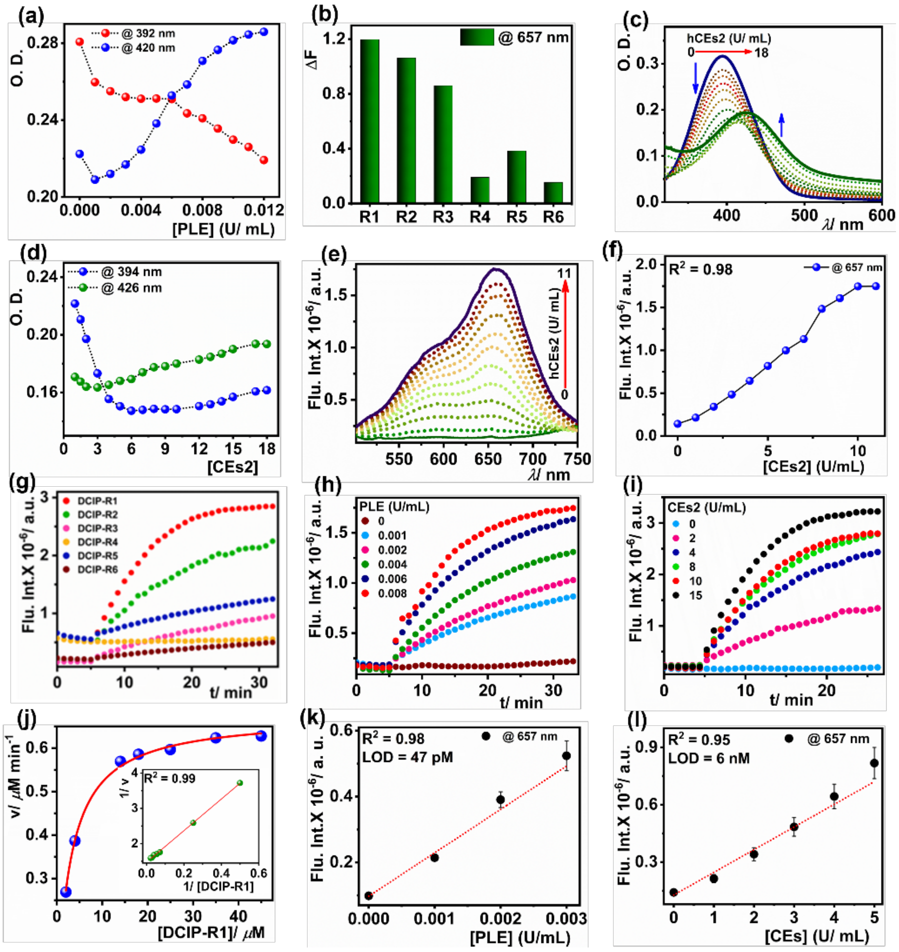

Figure S26: (a) Change in the absorption of **DCIP-R1** at 392 nm and 420 nm upon gradual addition of PLE**, (b)** The fluorogenic response of **DCIP-R** probes with PLE (0.01 U/mL) at 657 nm after 30 min incubation, **(c)** Absorption spectra of **DCIP-R1** with increasing concentration of hCEs2. **(d)** Change in the absorption of **DCIP-R1** upon hCEs2 addition at 394 nm and 426 nm, **(e)** change in emission of DCIP-R1 upon hCEs2 addition, **(f)** enhancement in the emission intensity of **DCIP-R1** with hhCEs2 at 657 nm, **(g)** kinetic studies of all the **DCIP-R** probes with hCEs2 (10 U/ mL) monitored at 657 nm, **(h)** and **(i)** Kinetic studies of **DCIP-R1** with different concentrations of PLE and hCEs2 respectively, All the emission kinetics were monitored at 657 nm (λ_{ex} = 480 nm), **(j)** Michaelis Menten plot for **DCIP-R1** catalysis by PLE (0.008 U/mL) (inset: Double reciprocal Lineweaver-Burk plot) **(k)** and **(l)** LOD for detection of hCEs2 and PLE respectively using **DCIP-R1**. All the experiments are performed in HEPES buffer (100 mM; pH = 8.0) at 37 ºC; λ_{ex} = 480 nm and the concentration of PLE is expressed in terms of enzyme units, [**DCIP-R6**] = 10 μM.

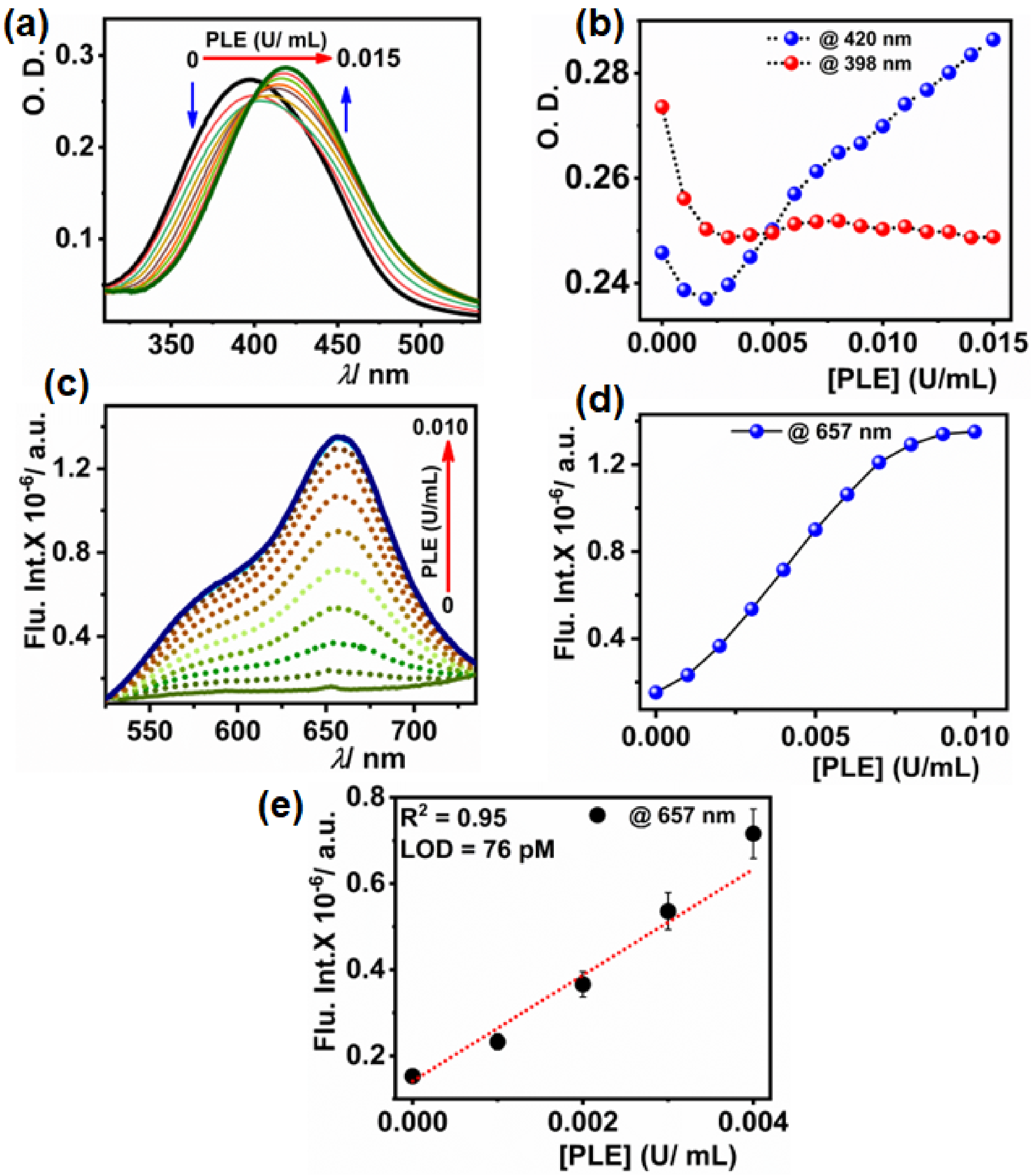

Figure S27: (a) UV-Vis. absorption spectra and **(c)** emission spectra of **DCIP-R2** as a function of PLE concentration, **(b)** change in the absorption of the probes at 420 nm and 398 nm upon PLE addition, **(d)** enhancement in the emission intensity at 657 nm upon PLE addition, **(e)** LOD for detection of PLE using **DCIP-R2**. All the experiments are performed in HEPES buffer (100 mM; pH = 8.0) at 37 °C; λ_{ex} = 480 nm and the concentration of PLE is expressed in terms of enzyme units, [**DCIP-R2**] = 10 μM.

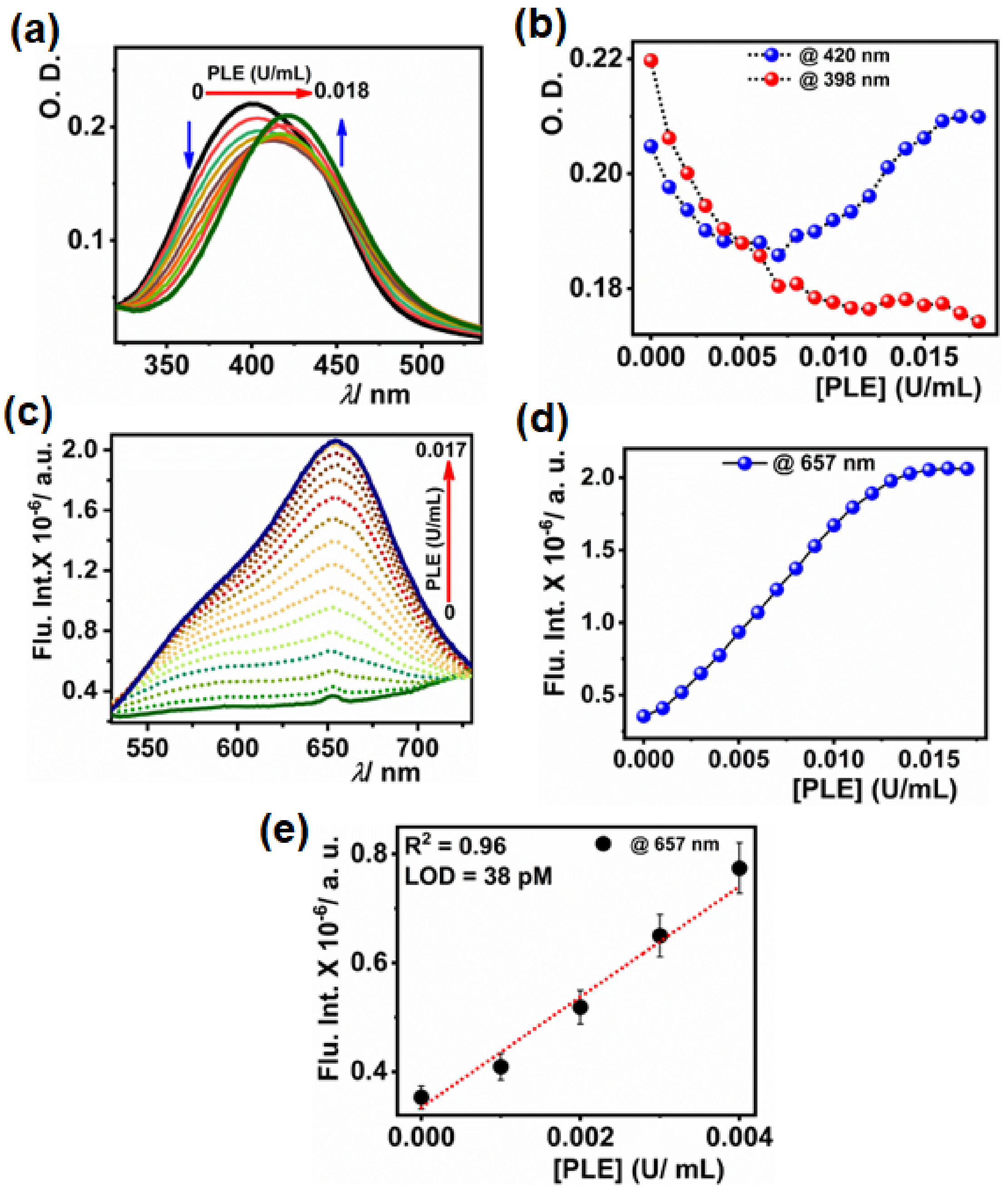

Figure S28: (a) UV-Vis. absorption spectra and **(c)** emission spectra of **DCIP-R3** as a function of PLE concentration, **(b)** change in the absorption of the probes at 420 nm and 398 nm upon PLE addition, **(d)** enhancement in the emission intensity at 657 nm upon PLE addition, **(e)** LOD for detection of PLE using **DCIP-R3**. All the experiments are performed in HEPES buffer (100 mM; pH = 8.0) at 37 °C; λ_{ex} = 480 nm and the concentration of PLE is expressed in terms of enzyme units, [**DCIP-R3**] = 10 μM.

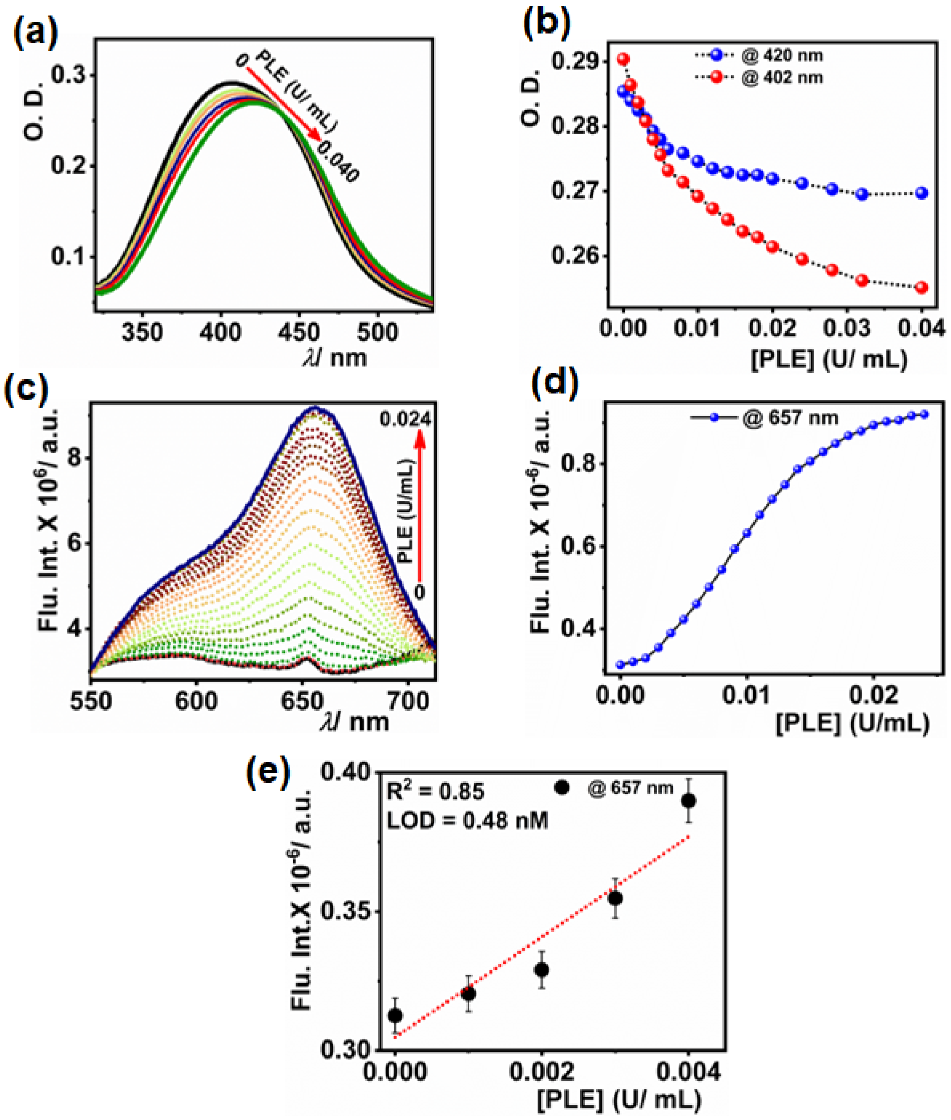

Figure S29: (a) UV-Vis. absorption spectra and **(c)** emission spectra of **DCIP-R4** as a function of PLE concentration, **(b)** change in the absorption of the probes at 420 nm and 398 nm upon PLE addition, **(d)** enhancement in the emission intensity at 657 nm upon PLE addition, **(e)** LOD for detection of PLE using **DCIP-R4**. All the experiments are performed in HEPES buffer (100 mM; pH = 8.0) at 37 ºC; λ_{ex} = 480 nm and the concentration of PLE is expressed in terms of enzyme units, [**DCIP-R4**] = 10 μM.

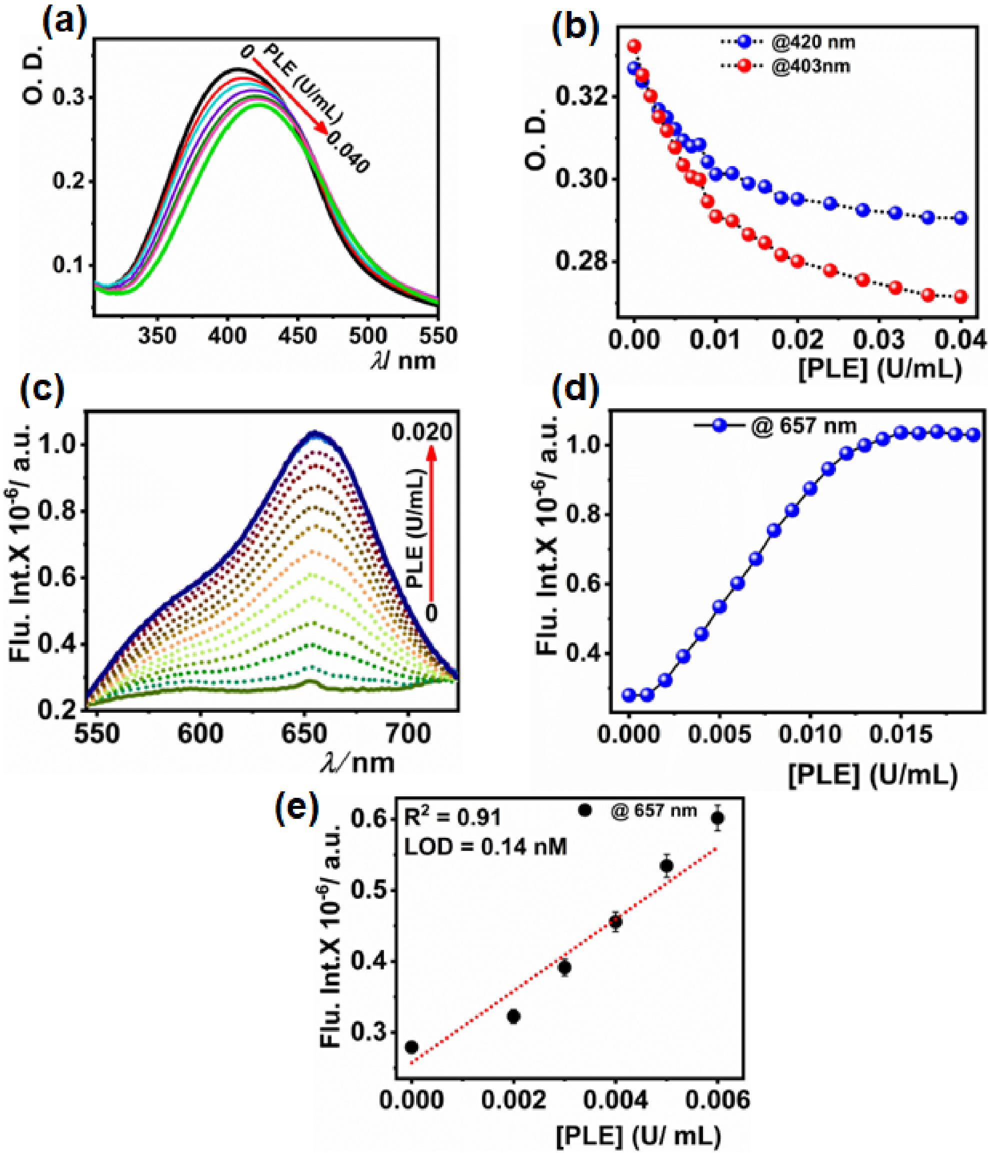

Figure S30: (a) UV-Vis. absorption spectra and **(c)** emission spectra of **DCIP-R5** as a function of PLE concentration, **(b)** change in the absorption of the probes at 420 nm and 398 nm upon PLE addition, **(d)** enhancement in the emission intensity at 657 nm upon PLE addition, **(e)** LOD for detection of PLE using **DCIP-R5**. All the experiments are performed in HEPES buffer (100 mM; pH = 8.0) at 37 ºC; λ_{ex} = 480 nm and the concentration of PLE is expressed in terms of enzyme units, [**DCIP-R5**] = 10 μM.

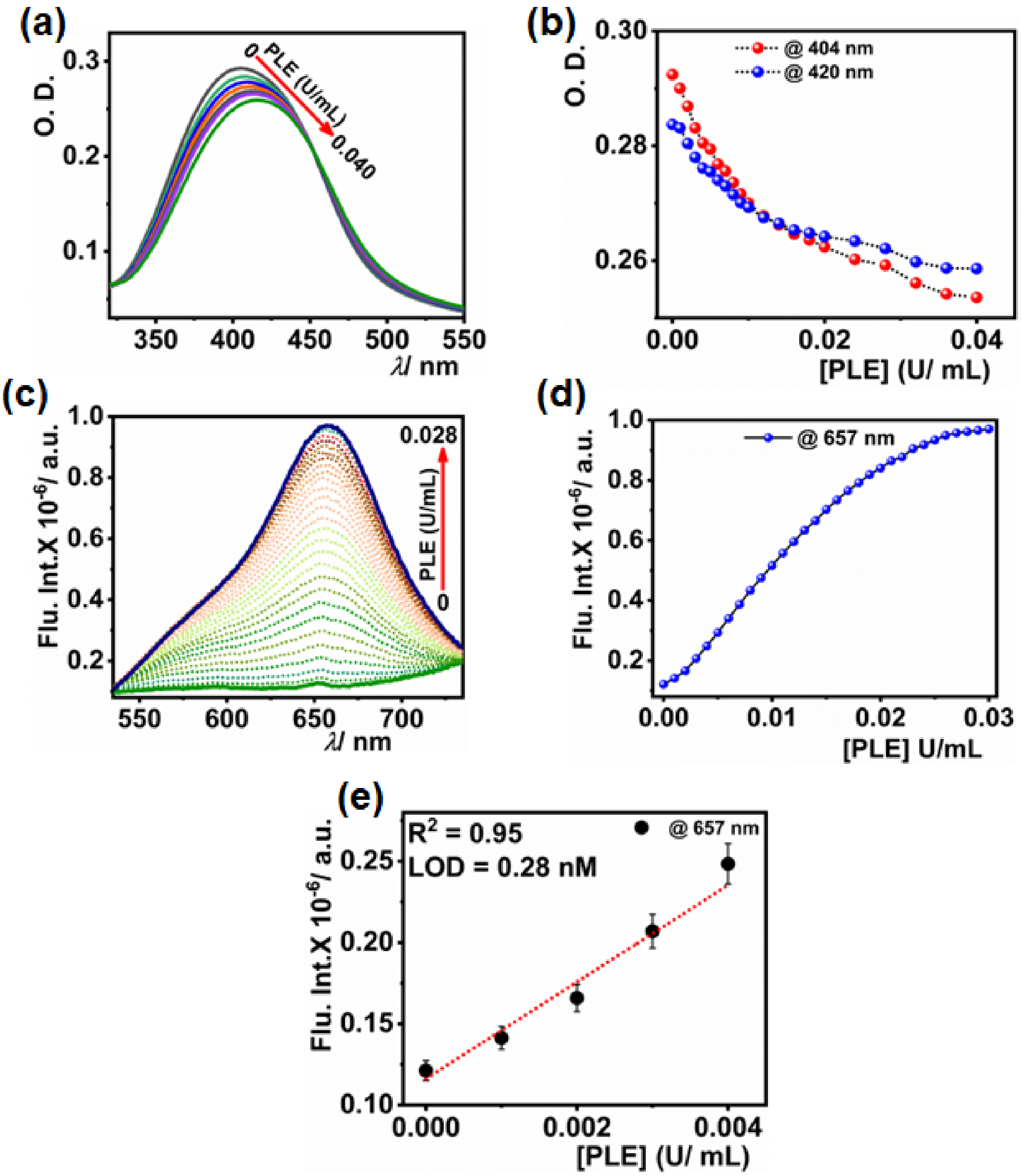

Figure S31: (a) UV-Vis. absorption spectra and **(c)** emission spectra of **DCIP-R6** as a function of PLE concentration, **(b)** change in the absorption of the probes at 420 nm and 398 nm upon PLE addition, **(d)** enhancement in the emission intensity at 657 nm upon PLE addition, **(e)** LOD for detection of PLE using **DCIP-R6**. All the experiments are performed in HEPES buffer (100 mM; pH = 8.0) at 37 ºC; λ_{ex} = 480 nm and the concentration of PLE is expressed in terms of enzyme units, [**DCIP-R6**] = 10 μM.

7. Mechanism for esterase-catalyzed hydrolysis of an ester substrate

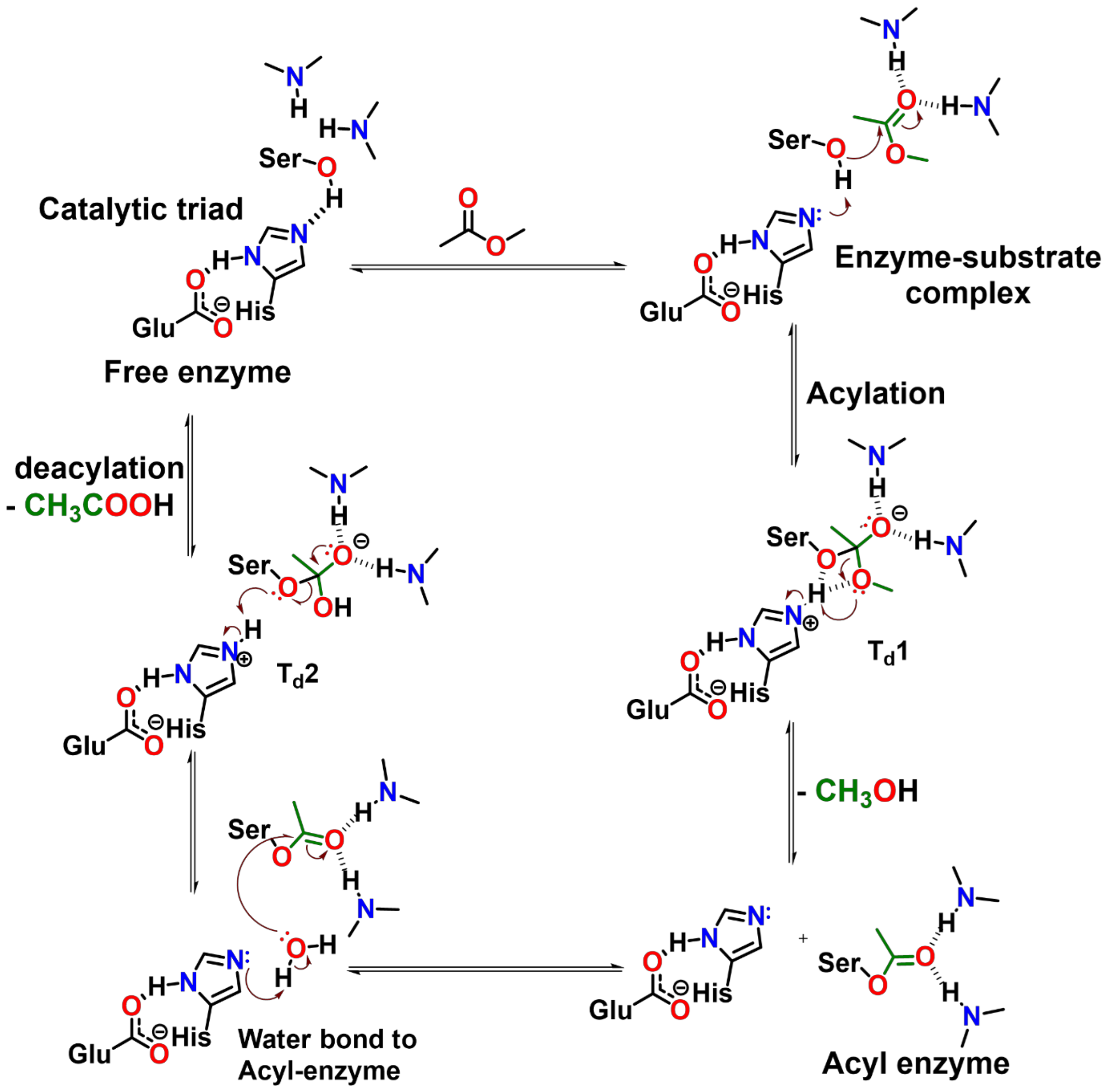

Scheme S2: Scheme depicting the hCEs-catalysed hydrolysis of an ester substrate in an active pocket of the enzyme.

8. HRMS of compound DCIP-R1 with PLE

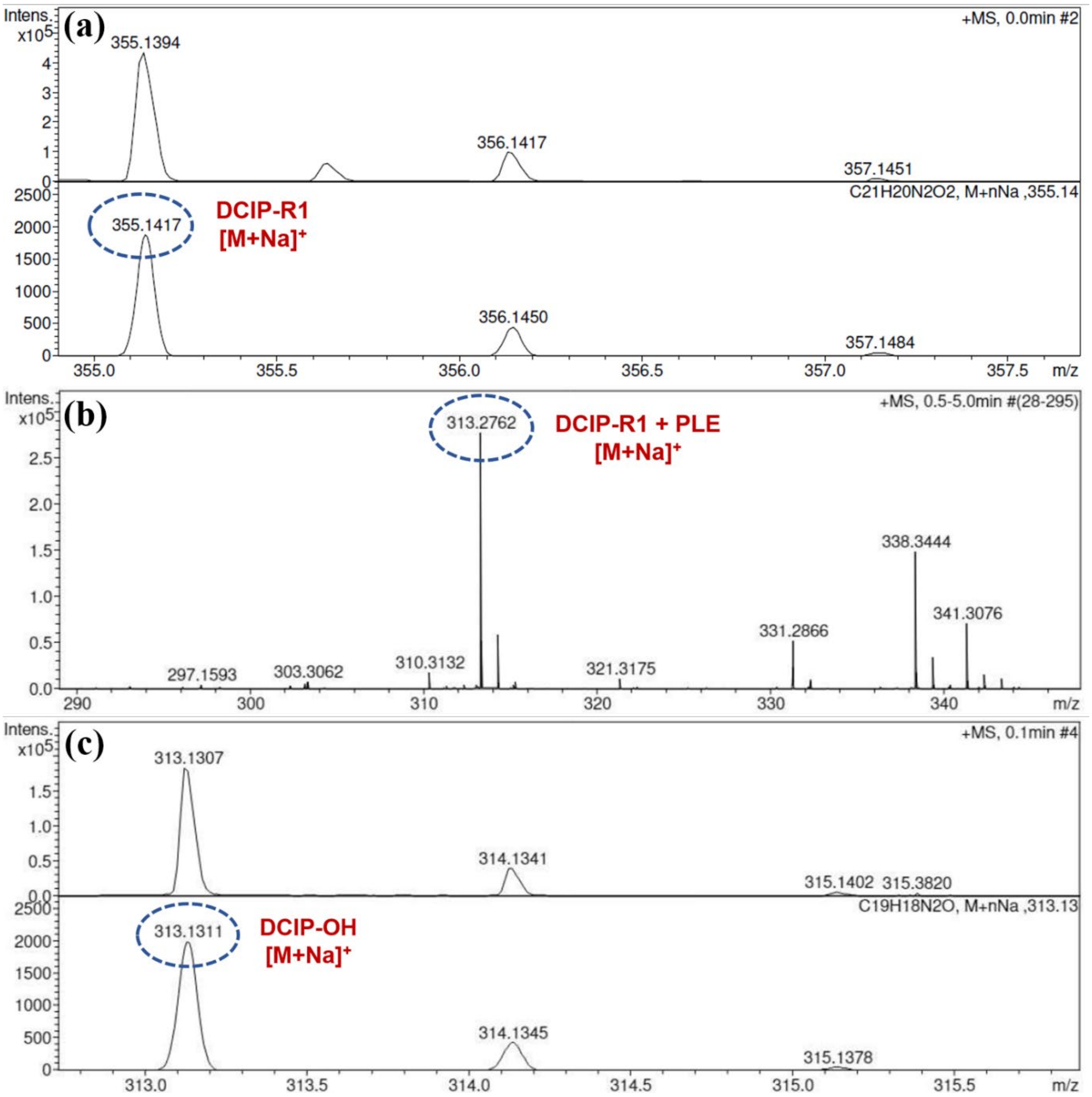

Figure S32: HRMS of **(a)** compound **DCIP-R1**, **(b)** the product obtained after the reaction of **DCIP-R1** with PLE, and **(c)** compound **DCIP-OH**.

9. HPLC profile of DCIP-R probes catalyzed by PLE.

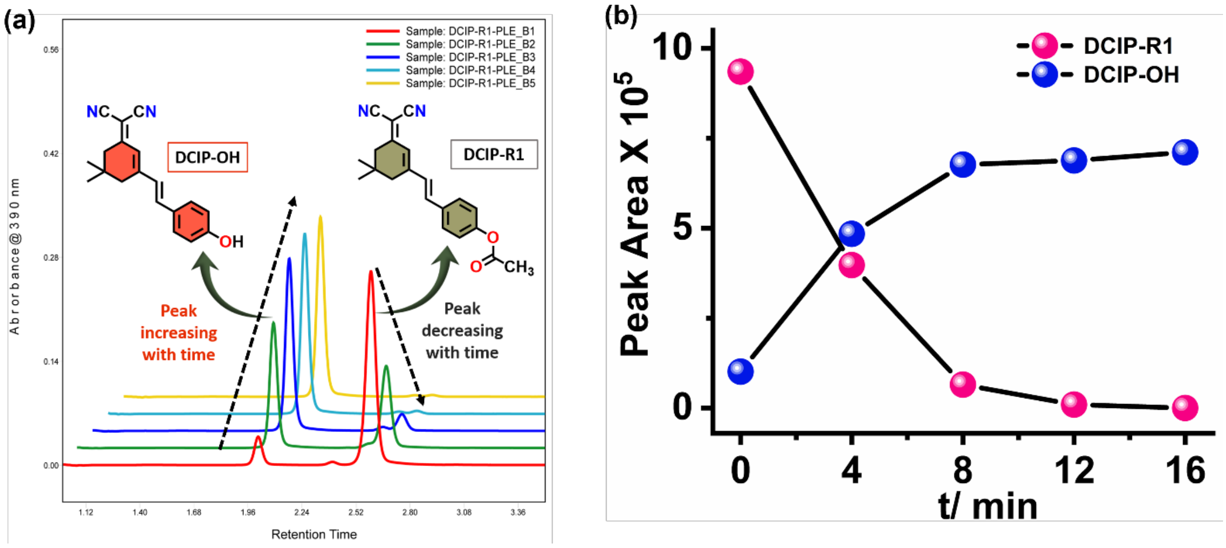

Figure S33: HPLC analysis of **(a) DCIP-R1** in the presence of the enzyme PLE, **(b)** plots depicting the change in peak area against the progress of time for the chromatographic peaks with retention time.

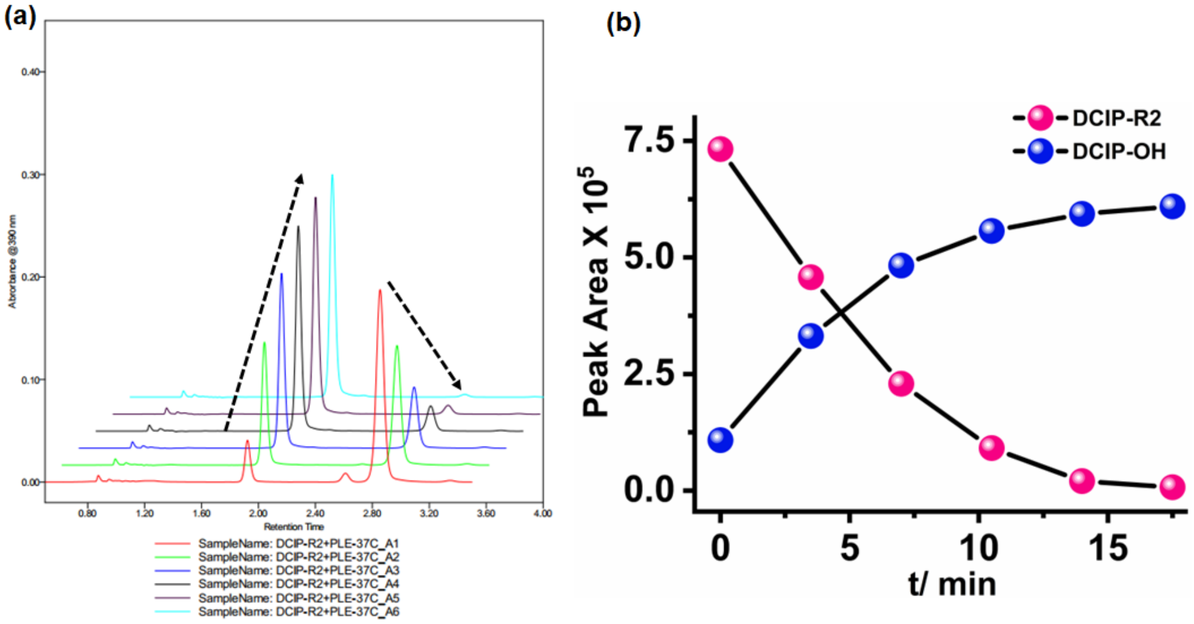

Figure S34: HPLC analysis of **(a) DCIP-R2** in the presence of the enzyme PLE, **(b)** plots depicting the change in peak area against the progress of time for the chromatographic peaks with retention time.

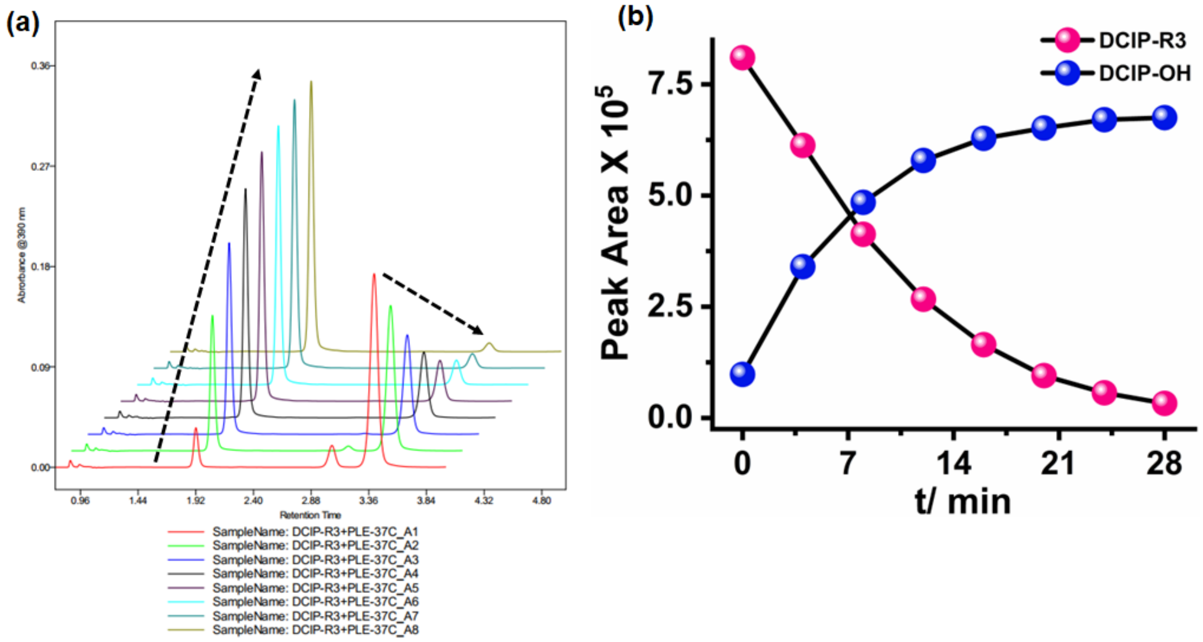

Figure S35: HPLC analysis of **(a) DCIP-R3** in the presence of the enzyme PLE, **(b)** plots depicting the change in peak area against the progress of time for the chromatographic peaks with retention time.

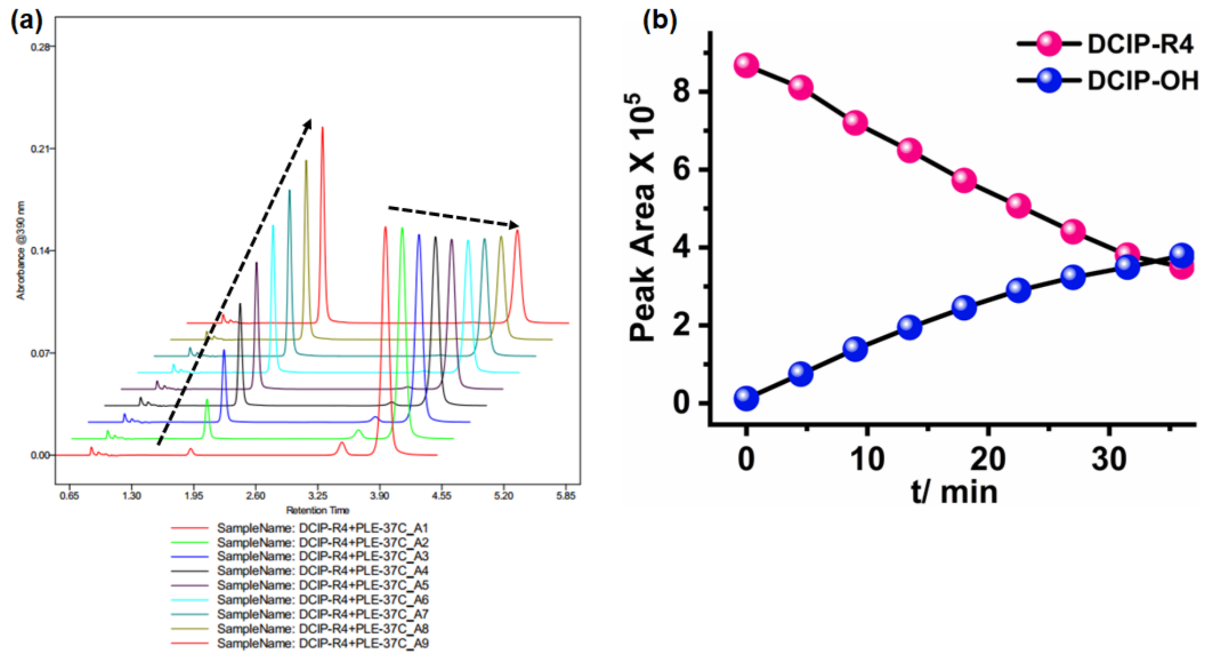

Figure S36: HPLC analysis of **(a) DCIP-R4** in the presence of the enzyme PLE, **(b)** plots depicting the change in peak area against the progress of time for the chromatographic peaks with retention time.

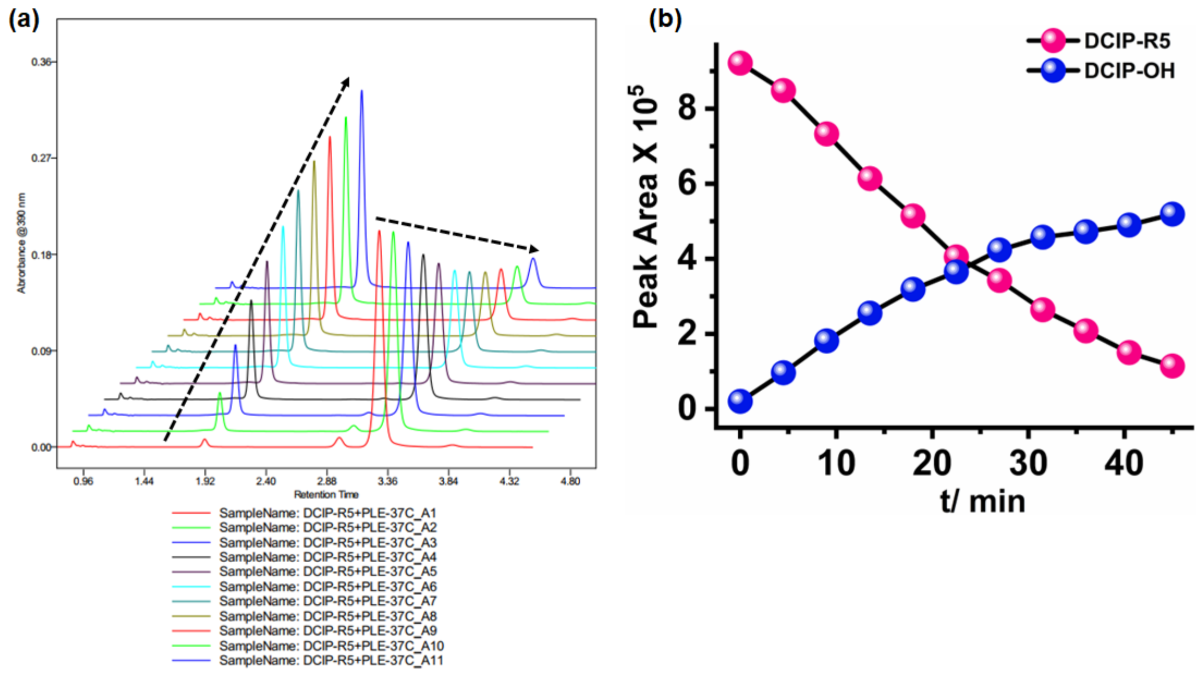

Figure S37: HPLC analysis of **(a) DCIP-R5** in the presence of the enzyme PLE, **(b)** plots depicting the change in peak area against the progress of time for the chromatographic peaks with retention time.

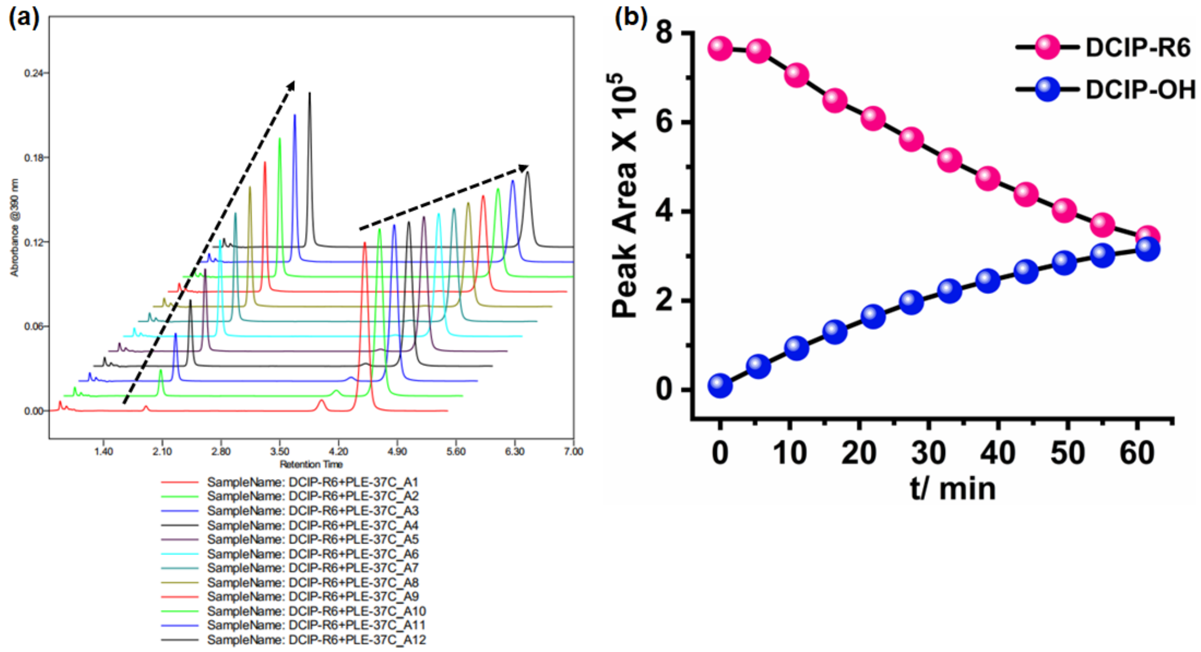

Figure S38: HPLC analysis of **(a) DCIP-R6** in the presence of the enzyme PLE, **(b)** plots depicting the change in peak area against the progress of time for the chromatographic peaks with retention time.

10. Live cell imaging Studies

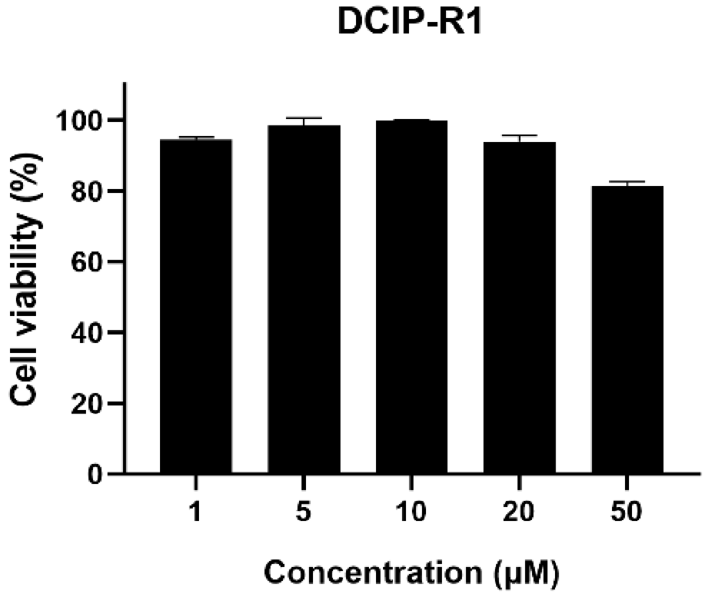

Figure S39: CCK-8 assay of HepG2-cell viability after incubation with different concentrations of **DCIP-R1** for 24 h.

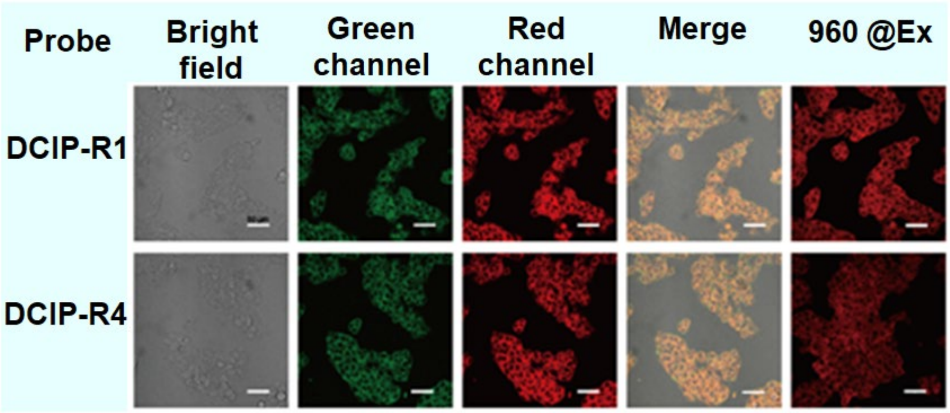

Figure S40: Fluorescence images of HepG2 cells stained with 1 μM of **DCIP-R1** (upper panel), and **-R4** (lower panel) respectively for 20 min at 37 °C. Images were obtained by single-photon microscopy: λ_{ex} = 480 nm, λ_{em} = 500-550 nm for the green channel, λ_{em} = 570-620 nm for the red channel; and two-photon microscopy: λ_{ex} = 960 nm, λ_{em} = 604-678 nm for the red channel. Scale bars = 50 μm.

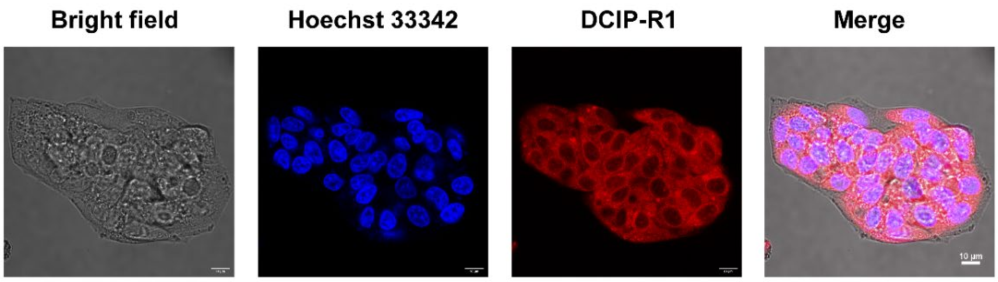

Figure S41: Single-photon Fluorescence images of HepG2 cells co-labeled with 1 μM of **DCIP-R1** and 10 μM of Hoechst 33342 for 10 min at 37 °C.

11. *In vivo* studies

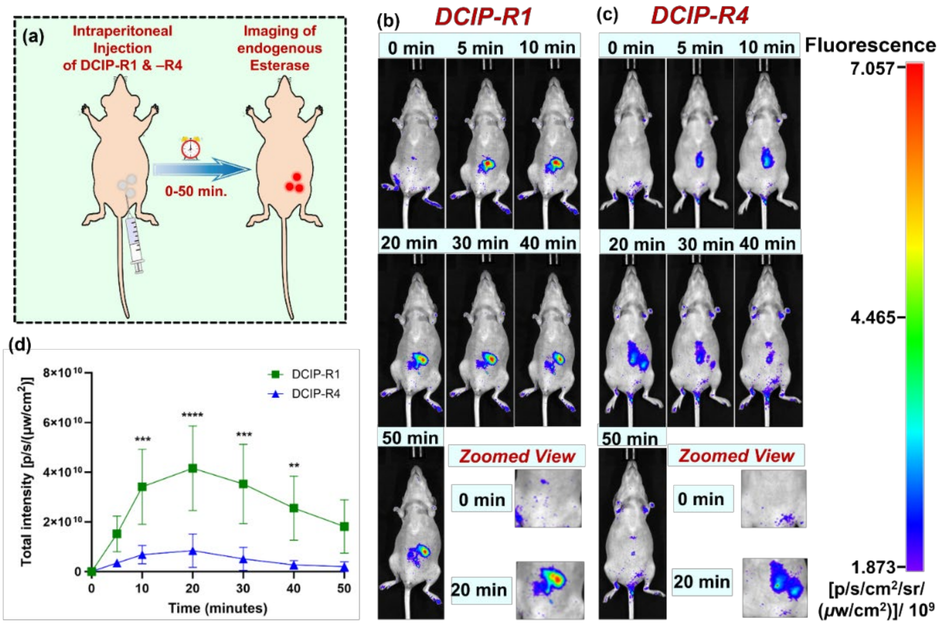

Figure S42. (a) Scheme showing the intraperitoneal injection of probes to healthy nude mice, Fluorescence imaging of mice treated with 1 mM of (b) **DCIP-R1**, (c) **-R4** (n=6) at 0-50 minutes post injection, (d) Quantification of fluorescence intensity obtained from (b) and (c). Data are presented as mean ± SD. Significant differences were analyzed using two-way ANOVA with Sidak's multiple comparisons test. **$p < 0.01$; ***$p < 0.001$; ****$p < 0.001$.

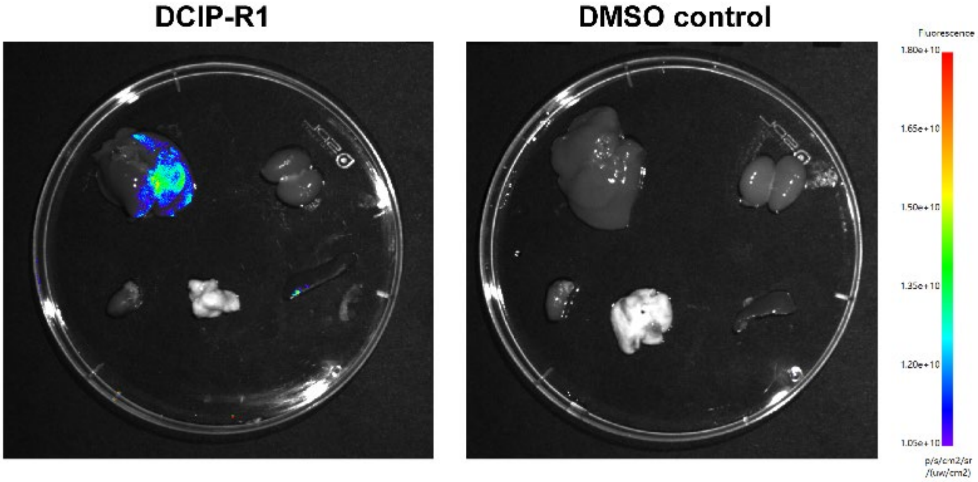

Figure S43: Fluorescence imaging of mouse organs, including liver (top left), kidney (top right), heart (bottom left), lung (bottom middle) and spleen (bottom right), 50 minutes post IP injection of the **DCIP-R1** probe.

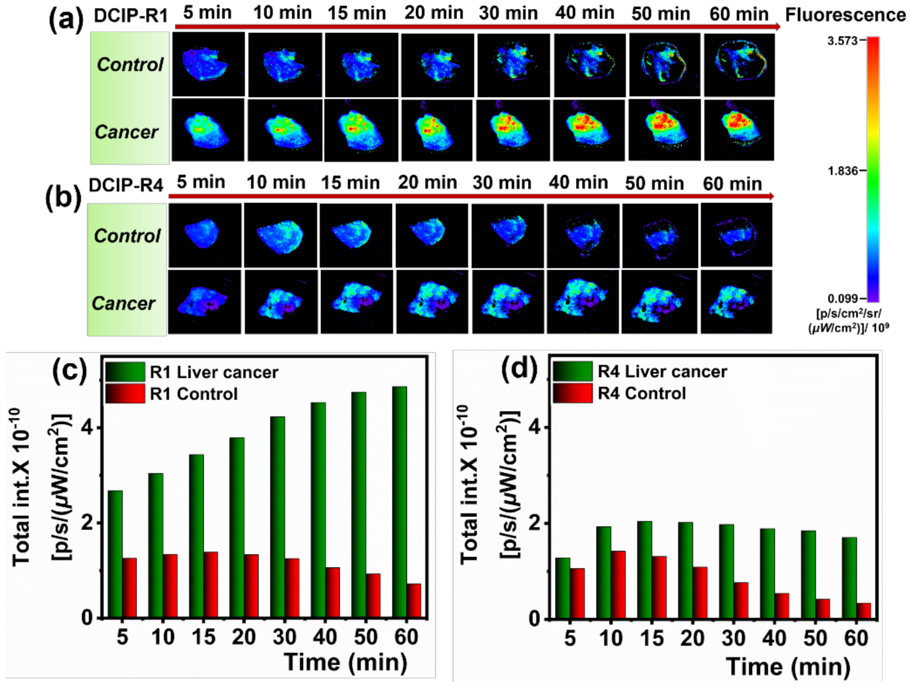

Figure S44: Fluorescence imaging of rat liver tumor slices treated with (a) **DCIP-R1**, (b) **DCIP-R4**, (c) and (d) Quantification of fluorescence intensity obtained from (a) and (b).

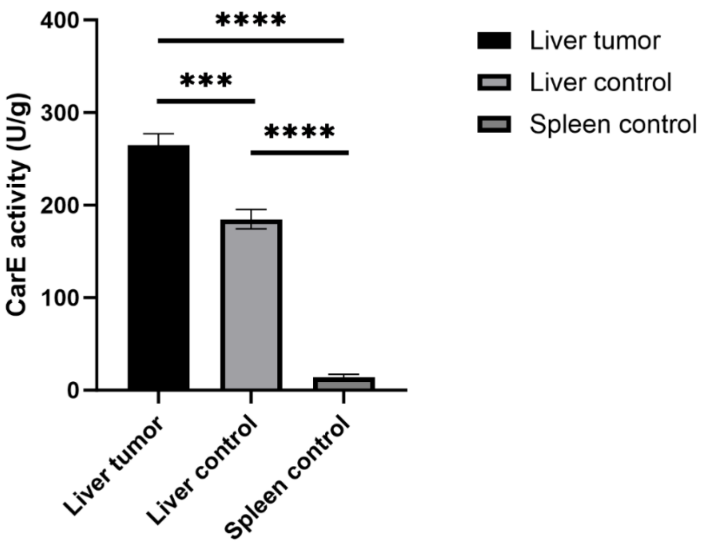

Figure S45: Measurement of carboxylesterase (CEs) levels in liver tumors, healthy liver and healthy spleen *via* a commercial kit. Data are presented as mean ± SD. Significant differences were analyzed using one-way ANOVA with Tukey's multiple comparisons test. ***p <0.001, ****p <0.0001.

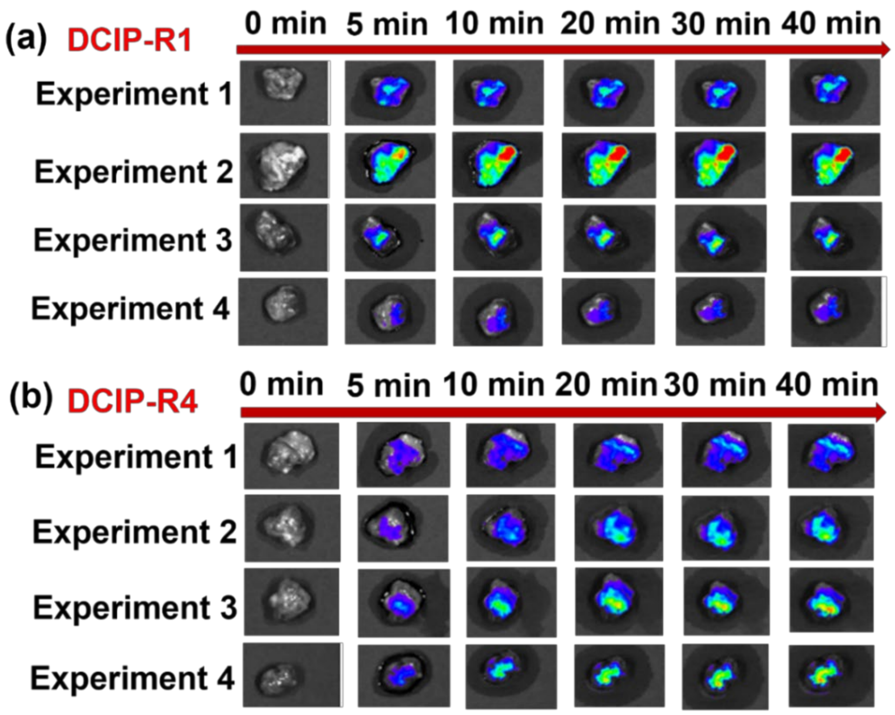

Figure S46: Fluorescence imaging of mouse HepG2 liver tumor slices using **(a) DCIP-R 1** and **(b) -R4**.

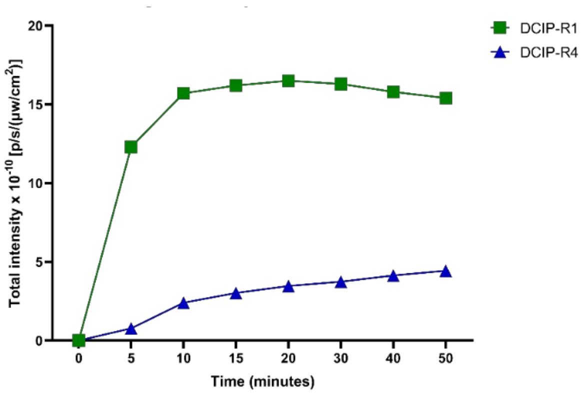

Figure S47: Quantification of fluorescence intensity obtained from experiment 2 in **Figure S46**.

12. Table 1: Comparison of literature reports with our probe

Probe	λ_{abs}^{max} / nm	λ_{em}^{max} / nm	Stokes Shift/ nm	Enzyme Detected	LOD	Reference
**EP 1**	548	605	57	CEs2	-	Kailass. *et al.*[3]
**EP 2**	556	600	44	CEs	-	Halabi. *et al.*[4]
**EP 3**	373	455	82	CEs2	7.7 nM	Park. *et al.*[5]
**EP 4**	530	595	65	CEs1	-	Tian. *et al.*[6]
**EP 5**	-	-	-	CEs1	0.01 μg/mL	Wang. *et al.*[7]
**EP 6**	600	662	62	CEs2	0.07 μg/mL	Jin. *et al.*[8]
**EP 7**	342 or 452	564/ 416	-	CEs2	1 μg/mL	Yin. *et al.*[9]
**EP 8**	354	542/ 452	188	CEs2	12 ng/mL	Jin. *et al.*[10]
**EP 9**	473	589	116	CEs1	27.8 ng/mL	Dai. *et al.*[11]
**DCIP-R1**	480	657	177	PLE	7.9 ng/mL (47 pM)	This work
				CEs2	0.36 μg/mL (6 nM)	

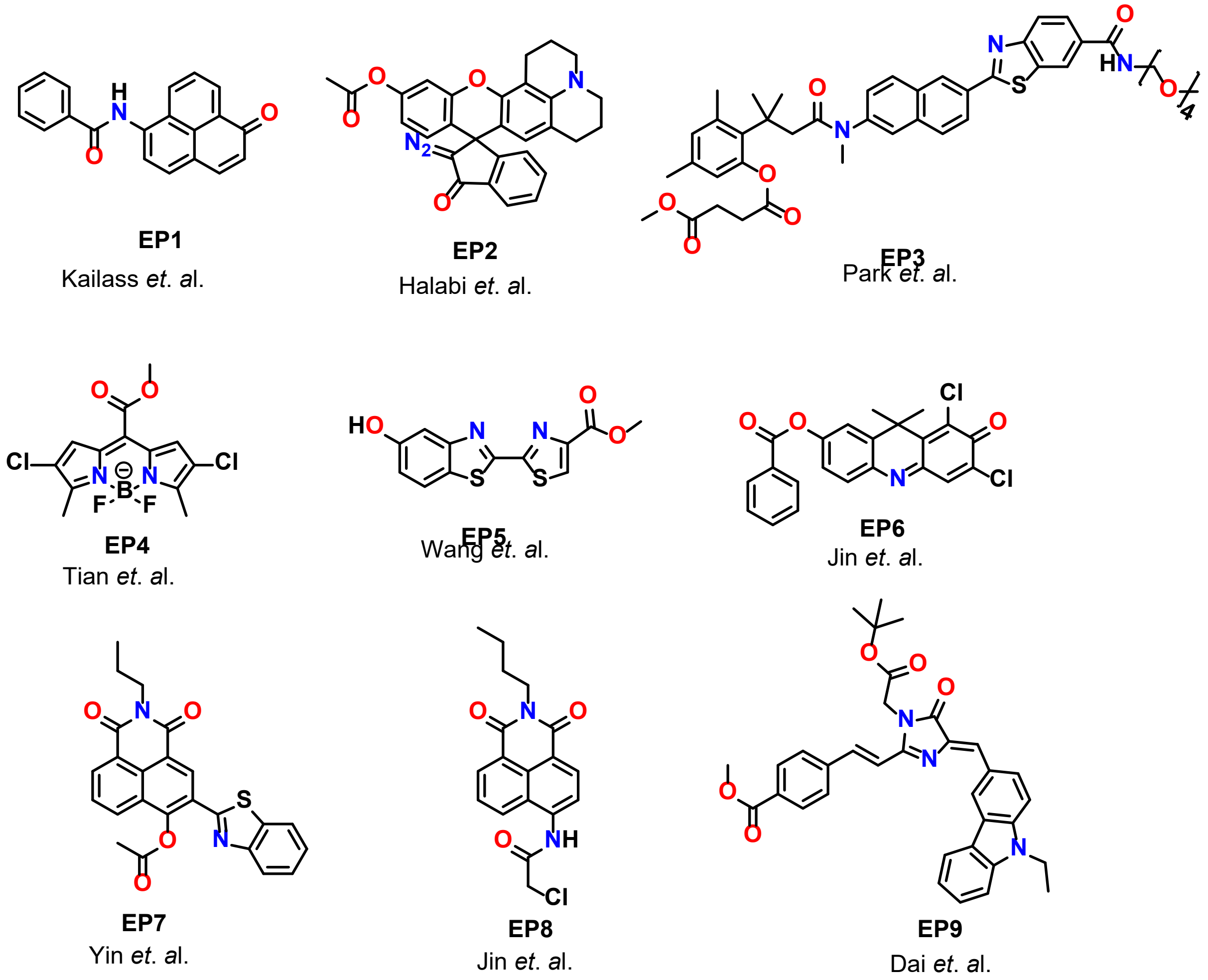

Figure S48: Some reported fluorogenic probes for sensing esterase activity

References

1. P. C. Wu *et al.*, Noninvasive assessment of liver function reserve with fluorescent dosimetry of indocyanine green. *Biomed. Opt. Express* **2022**, *13*, 1995-2005.
2. H. Li et al., Lighting-Up Tumor for Assisting Resection via Spraying NIR Fluorescent Probe of γ-Glutamyltranspeptidas. *Front. Chem.* **2018**, *6*, 485-500.
3. K. Kailass *et al.*, Measuring human carboxylesterase 2 activity in pancreatic cancer patient-derived xenografts using a ratiometric fluorescent chemosensor. *Chem. Sci.* **2019**, *10*, 8428-8437.
4. E. A. Halabi, Z. Thiel, N. Trapp, D. Pinotsi, P. Rivera-Fuentes, A Photoactivatable Probe for Super-Resolution Imaging of Enzymatic Activity in Live Cells. *J. Am. Chem. Soc.* **2017**, *139*, 13200-13207.
5. N. H. Park *et al.*, Addressing Drug Resistance in Cancer with Macromolecular Chemotherapeutic Agents. *J. Am. Chem. Soc.* **2018**, *140*, 4244-4252.
6. Z. Tian *et al.*, Rational Design of a Long-Wavelength Fluorescent Probe for Highly Selective Sensing of Carboxylesterase 1 in Living Systems. *Anal. chem.* **2019**, *91*, 5638-5645.

7. D.-D. Wang *et al.*, A bioluminescent sensor for highly selective and sensitive detection of human carboxylesterase 1 in complex biological samples. *Chem. Comm.* **2016,** *52*, 3183-3186.
8. Q. Jin *et al.*, A highly selective near-infrared fluorescent probe for carboxylesterase 2 and its bioimaging applications in living cells and animals. *Biosens. and Bioelectron.* **2016**, *83*, 193-199.
9. Y. Yin *et al.*, Development of an esterase fluorescent probe based on naphthalimide-benzothiazole conjugation and its applications for qualitative detection of esterase in orlistat-treated biosamples. *Anal. Chim. Acta* **2022**, *1190*, 339248.
10. Q. Jin *et al.*, A Two-Photon Ratiometric Fluorescent Probe for Imaging Carboxylesterase 2 in Living Cells and Tissues. *ACS Appl. Mater. Interfaces* **2015**, *7*, 28474-28481.
11. J. Dai *et al.*, Detection of carboxylesterase 1 and carbamates with a novel fluorescent protein chromophore based probe. *Dyes Pigm.* **2021**, *192*, 109444.
